# Cost-effectiveness analysis of pharmaceutical care for hypertensive patients from the perspective of the public health system in Brazil

**DOI:** 10.1371/journal.pone.0193567

**Published:** 2018-03-06

**Authors:** Maurílio de Souza Cazarim, Leonardo Régis Leira Pereira

**Affiliations:** Department of Pharmaceutical Sciences, School of Pharmaceutical Sciences of Ribeirão Preto, University of São Paulo, Ribeirão Preto, state of São Paulo, Brazil; Karolinska Institutet, SWEDEN

## Abstract

**Introduction:**

Only 20% of patients with systemic arterial hypertension (SAH) have blood pressure within recommended parameters. SAH has been the main risk factor for morbidity and mortality of cardiovascular diseases, which affects the burden of the Public Health System (PHS). Some studies have shown the effectiveness of Pharmaceutical Care (PC) in the care of hypertensive patients.

**Objective:**

To perform a cost-effectiveness analysis to compare SAH treatment with PC management and conventional treatment for hypertensive patients offered by the PHS.

**Methods:**

A cost-effectiveness study nested to a quasi-experimental study was conducted, in which 104 hypertensive patients were followed up in a PC program. Blood pressure control was considered as the outcome for the economic analysis and the costs were direct and non-direct medical costs.

**Results:**

PC was dominant for two years in the post-PC period compared with the pre-PC year. The mean cost effectiveness ratio (CER) for the CER_Pre-PC_, CER_PC_, and CER_Post-PC_ periods were: US$ 364.65, US$ 415.39, and US$ 231.14 respectively. The incremental cost effectiveness ratio (ICER) analysis presented ICER of US$ 478.41 in the PC period and US$ 42.95 in the post PC period. Monte Carlo sensitivity analysis presented mean ICER_PC_ and ICER_Post-PC_ equal to US$ 605.09 and US$ 128.03, reaching US$ 1,725.00 and US$ 740.00 respectively.

**Conclusion:**

Even for the highest ICER, the values were below the cost effectiveness threshold, which means that PC was a cost effective strategy for the care of hypertensive patients in the PHS.

## Introduction

Non-communicable chronic diseases cause 38 million deaths worldwide annually and cardiovascular diseases (CVDs) represent 28% of these deaths [1, 2, 3, 4]. In this context, systemic arterial hypertension (SAH) is relevant because it is one of the main CVDs associated with cardiovascular deaths [5, 6, 7]. In Brazil, CVDs represent 72% of all causes of death annually [2] and SAH prevalence is estimated at 21.4%. Considering the undiagnosed individuals this rate can reach 33% of Brazilians [5, 6, 7, 8].

According to the World Health Organization—WHO (2011), uncontrolled blood pressure is the main problem in the management of SAH as it can increase the risk for CVD exponentially [9, 10]. It is estimated that the percentage of hypertensive patients who have blood pressure within the parameters recommended by the 7^th^ guideline of the Brazilian Society of Cardiology (BSC) is 20–30%, which is considered a low rate [7, 11]. In addition, studies have shown satisfactory blood pressure control in less than 25% of hypertensive patients in Brazil [12, 13].

Poor blood pressure control has resulted in increased spending for the Brazilian public health system (PHS). An estimated 1.2 million hospitalizations in Brazil are linked to CVDs associated with SAH. This represents an annual cost of 780 million dollars for the health systems, 462 million dollars for the Brazilian PHS. Considering the total health expenditure in the country was approximately 7% of GDP in proportion to the year 2015, this commitment represents 0.2% of GDP spent on SAH [7, 14].New strategies and health policies to promote preventive care such as empowerment in patients with the disease, and health education have been proposed to the PHS to improve the CVD scenario in Brazil [7, 15, 16]. In this context Pharmaceutical Care (PC) has been highlighted as the professional practice model that applies medication therapy management, which aims to promote better patient care through systematized consultations [15].

Many studies have shown that PC is effective for blood pressure control and, consequently, for reducing cardiovascular risk associated with SAH [16, 17, 18]. PC grounded in patient education care can improve patient compliance up to 100% and also promote preventive care that is cheaper than spending on treatment complications for SAH [7, 19]. Furthermore, PC was able to improve from 54.0% to 98.0% patients with satisfactory blood pressure [19].

Therefore, PC has been a health technology (HT) capable of providing improvement in the control of chronic diseases, especially SAH [15, 16, 17, 19]. Thus, the PC impact in outcomes such as blood pressure and cardiovascular risk can reduce morbidity and mortality caused by SAH [19, 20, 21, 22]. However, there is the need to assess outcomes in face of costs for PC in SAH care to provide evidence whether PC is an effective choice for the PHS. Additionally, pharmacoeconomics is a rationale for decision making to provide the incorporation of new HTs such as PC in the PHS [23, 24, 25]. The objective of this study was to perform a cost-effectiveness analysis to compare SAH treatment with PC management and conventional treatment for hypertensive patients offered by the PHS.

## Methods

### Methodological design and study delineation

This is a cost-effectiveness study nested to a quasi-experimental study of a pharmaceutical care program’s impact on patients’ success to reach their blood pressure goal, which was conducted from the Brazilian PHS perspective. Data for this study were collected from March to November 2014. In 2009 a clinical trial of non-pharmacological intervention was performed with 104 hypertensive patients, of which the PC program for hypertensive patients characterized the intervention [19, 26]. This clinical trial provided a quasi-experimental study which analyzed data of 104 hypertensive patients during the years 2006 to 2012 [19]. Both studies were included in the study of this pharmacoeconomic data base and were developed in the Pharmaceutical Care Research Center and Clinical Pharmacy (*Centro de Pesquisa em Assistência Farmacêutica e Farmácia Clinica*) (CPAFF), Faculty of Pharmaceutical Sciences of Ribeirão Preto, University of São Paulo (USP-FCFRP), “S1 Appendix”.

### Pharmaceutical care program for hypertensive patients

According to Cazarim et al. (2016), individuals diagnosed with SAH living in Ribeirão Preto, in the state of São Paulo (SP), aged >20 years, with ongoing monitoring of their SAH, who had PHS coverage, and were using antihypertensive medication were included in the program. Patients who were unable to be cared for in the study health unit, pregnant women, and patients with cognitive problems were excluded [19].

The PC program was implemented in two PHS primary health care units in the city of Ribeirão Preto-SP. Twelve pharmaceutical consultations were planned based on the pharmacotherapeutic follow-up strategies of the North American model, Pharmacists' Workup [19]. One pharmacist was responsible for conducting the consultations, which occurred monthly for each patient. The initial consultation consisted of the collection of socio-demographic data, clinical history and life habits, followed by 11 consultations relevant to pharmacotherapeutic follow-up: blood pressure measurements and cardiovascular risk measures, analysis of medications and test results, education in health matters with guidelines on patient behavior regarding life habits, adherence to treatment and, when necessary, interventions in pharmacotherapy.

### Outcome assessed

Blood pressure control of the attended hypertensive patients was used as the outcome for the analysis of cost effectiveness. This outcome was measured in the percentage of patients with a mean of satisfactory blood pressure measurements. The parameters used for the definition of satisfactory blood pressure control rating were set by the policy regarding the VIII Joint National Committee 2014—VIII JNC. Blood pressure values lower than 150 x 90 mmHg for hypertensive individuals over 60 years of age without diabetes and without chronic kidney disease, and lower than 140 x 90 mmHg for individuals over 18 years with hypertension, associated or not with diabetes and chronic kidney disease, were understood as having satisfactory blood pressure control [27].

### Costing conducted in the study

Details of the costs were collected in the system at the Municipal Department of Health of Ribeirão Preto (SMS-RP) and the PHS unified table, using as a basis the year 2013 [28]. For cost adjustment for the year 2015 the National Consumer Price Index (NCPI) was considered, available in economic indicators consolidated by the Central Bank of Brazil [29]. The conversion into US dollars was made to scale the costs to the current economy. The value was calculated based on the value of the currency by the Central Bank of Brazil, with US$ 1.00 equal to R$ 3.87 in the consolidation of 2015 [24, 30, 31]. The same was undertaken to make comparable costs in the discussion of results of other studies [32, 33, 34].

The costs considered are non-direct medical costs, being absenteeism in not attending scheduled appointments, and public transport to arrive for and return from the appointment (all being related to charges for public health); and direct medical costs, being laboratory tests recommended by the 7^th^ guideline of the Brazilian Society of Cardiology (2016) [7] for routine monitoring of hypertensive patients (cholesterol and fractions and triglycerides), antihypertensive medications provided by the PHS belonging to the Municipal Register of Essential Drugs of Ribeirão Preto (REMUME), and consultations in primary care (visits to general practitioners and family health doctors), emergency care (consultations for hypertensive crises in emergency care), and specialized care (specialty consultations on cardiology) were considered (Table 1).

**Table 1:** Costing and description of direct costs.

Cost type	Cost center	Description of cost	Cost sources and calculations
DIRECT MEDICAL COST	**Laboratory tests**	Tests used for diagnosis of dyslipidemia, recommended for the monitoring of systemic arterial pressure by the VII guideline of the Brazilian Society of Cardiology (2016) [7] to be regarded a morbidity associated as a risk factor for cardiovascular disease were considered. Thus, it included the tests for triglycerides, total cholesterol and LDL and HDL fractions.	Costs for these tests were obtained from the unified table of the Brazilian Public Health System [28]. The calculation was performed for each patient by multiplying the total number of annual examinations by their unit costs.
	**Consultation (primary care, specialized care and emergency care)**	Emergency care and specialized care were considered not to be generalizable in terms of costs for different morbidities. The hypertensive patient has a different cost in each of these segments and these costs consider the logistics of standardized care for the public health services. Therefore, the baseline cost of the emergency units and the baseline cost of the basic health unit for outpatient care were calculated. This cost refers to expenditures that are general to any type of patient who enters the unit to be attended to, the base cost of the health unit.	Costs of municipal data for each health unit in the city were obtained from the Finance Division and Operational Cost of Municipal Health Department, and the number of consultations broken down by sector/specialty of each health facility in the city by the IT Statistics, Control and Audit department of Municipal Health Department were obtained [28]. Base cost from utilities: the calculation was performed by dividing between the annual cost of the health unit weighted by the mean number of annual appointments for each health unit. This was performed separately for primary, specialized and emergency care units.
	Primary care	It was considered that our study was a general service for the patient's health condition in which there are few discrepancies between the mean cost of a hypertensive patient with the general mean of patients with other diseases.	Total expenditure in this study was calculated considering a primary health unit and family health strategy, as well as the total consultations in the health units. For the calculation, the base cost from primary health units was considered for each consultation, then multiplied by the number of primary consultations per patient per year.
	Emergency care	Consultation, the cost of medical care, nursing care, the examinations recommended by the Brazilian Society of Cardiology (2016) such as electrocardiogram (ECG), chest X-ray and creatine phosphokinase (CPK) were considered. Hydralazine hydrochloride 20 mg/ml, sodium nitroprusside 25 mg/ml, furosemide 10 mg/ml, captopril 25 mg, and clonidine 0.15 mg, all available on the Municipal Essentials Medicines List. For intravenous medication administration, the cost of 0.9% saline solution and nurse’s materials were considered [7].	Consultation and examination costs were obtained from the unified table of the Brazilian public health system. The cost of urgent drugs and nurse’s materials considered for the hypertensive emergency were obtained from the Pharmacy Division of Municipal Health Department, and the drug administering cost was obtained from the unified table of the Brazilian public health system [28]. The cost of urgent drugs was calculated by averaging the unit cost of the medication [28]. The urgent drugs, exams and consultation costs of emergency care were added to the base cost of the emergency care unit. The total was then multiplied by the number of emergency consultations per patient per year.
	Specialized Care	Refers only to cardiologist consultation. The electrocardiogram (ECG) examination recommended by the Brazilian Society of Cardiology (2016) [7] as a routine evaluation, nursing care, the consultation with the cardiologist, and medical monitoring were considered, as **cost category 1**. The monitoring of the hypertensive patient was interpreted in agreement with the VII guideline of the Brazilian Society of Cardiology (2016) [7]. Thus, routine exams (urinalysis, serum potassium, serum creatinine, uric acid and fasting glucose) for each year, **cost category 2,** and color Doppler ultrasound, transthoracic echocardiography and aortic arch angiography examinations every two years were considered, which comprise **cost category 3**. Categories 1 and 2 are recommended for further evaluation of clinical and subclinical lesions in the target organ [7]. It is noteworthy that ECG must be solicited in each specialized consultation routine examination every three consultations, and complex examinations of medical monitoring every six consultations. Three annual consultations with the cardiologist are recommended [7].	Cost categories 1, 2 and 3 were obtained from the unified table of the Brazilian public health system in 2013 [28]. Cost category 1 was calculated using its unit cost in each consultation. Cost category 2 was calculated taking its annual examination cost divided by three (refers to three annual consultations of the recommendations of VII guideline of the Brazilian Society of Cardiology (2016) [7]). The calculation of cost for category 3 was the same, but it was considered for two years, so every six consultations. Thus, cost categories 1, 2, 3 were summarized in the cost for consultation. The cost of specialized care consultation was calculated as the sum of the three category costs multiplied by the number of specialized consultations per patient per year.
	**Anti-hypertensive medications**	All anti-hypertensive medicines that the patients were taking comprised this cost. The annual consumption in milligrams of each antihypertensive medication per patient was considered.	The medicine cost was acquired from the acquisition report of the Pharmacy Division of the Municipal Health Department. For the calculation, the medicine unit cost was divided by the amount in milligrams corresponding to each drug to obtain the cost/milligram. Consumption was multiplied by the value in milligrams for each medication used by the patient to determine the cost of medication per patient year.
DIRECT NON-MEDICAL COST	**Transportation**	We considered that the patient would use public transportation to travel to and return from the consultations.	For the calculation the value of the flat rate charged in the municipality was used [35]. It was assigned for self-employed patients, the elderly not working, and retired, who use public transport and did not pay the public transport fees or for the ticket because they do not contribute to health taxes.
	**Absenteeism**	The cost of absenteeism was estimated according to the mean salary among those who worked as non-self employed, considering the percentage of them among the 104 patients from this study, to calculate the total cost and the patient in general.	The calculation was performed considering the Brazilian labor law to charge 8 hours of work/day, a month of vacation, 20 days of work in the month (excluding weekends and holidays). The mean annual salary amount, considering 13^th^ salary was divided by the total hours worked in the year, obtaining the value of hours worked. This value was multiplied by half a working day period i.e. four hours (considered as missing work to attend the consultation) [17, 20, 35].

The cost of PC was calculated for the structuring of this service in the health unit. Thus, the materials used for clinical care (scales, tape measure, sphygmomanometer), furniture (desk, chair, closet), general materials (computer and printer), office supplies (clipboards, pens, paper, folders, stapler, staples, hole punch, trash, ruler, text highlighter) were considered. For the calculation of expenditure on human resources with the pharmacist, the wage description with the rates and fees in accordance with SMS-RP was considered. The value set by the Regional Pharmacy Council (RPC) as pharmacists’ minimum salary for the state of São Paulo, “S2 Appendix” was envisioned as salary [24]. The PC cost was attributed for patient care in 2009 and was added to the cost of hypertensive patients to the health system for that year.

There was a cost data set missing for 53 patients at the end of the quasi-experimental study, subsequently the total annual cost was calculated for each year of the study (2006–2012) using the mean cost of each variable among 51 patients, which was multiplied by 104, that is the patient number attended by PC. Thus, costs were estimated as total and mean cost/patient/year.

### Analysis of the results

The costs were tabulated according to their type by year and period. The data were analyzed year by year and by period: from 2006 to 2008 being without PC that represented conventional health care offered by the PHS; 2009 represented the PC period; and the subsequent three years from 2010 to 2012 represented the post PC period. The mean cost per period and costs per patient were obtained by averaging each of the years of the period. It is emphasized that to calculate the mean outcome (blood pressure control per year) those patients who had measurements for all years of the study were considered. As for outcomes by period, patients who had measurements for the three periods were considered.

For the analysis, the cost-effectiveness ratio (CER) was calculated: CER = cost of health care ÷ percentage of blood pressure control achieved, representing the cost per blood pressure control achieved. Also, the incremental cost effectiveness ratio (ICER) was calculated: ICER = (cost of health care for PC—cost of conventional health care) ÷ (reach of pressure control in health care with PC—reach of pressure control in conventional health care), that represented the committed cost to have one more patient with blood pressure controlled in the year. Analyses by the cost-effectiveness plan were structure reasoned to the ratio of the calculated costs per patient in face of the effectiveness achieved in a comparison between the years of study [24, 31, 32, 33]. For the cost-effectiveness threshold, the value of GDP per capita related to the consolidation of 2015, US$ 10,240.43 [34] was considered, this value was tripled for the conformation of the cost effectiveness threshold as recommended by the Brazilian Evaluation of Technology in Health Network [31, 32].

### Sensitivity analysis

The incremental net benefit (INB), interpreted as the monetary benefit for each additional blood pressure control achieved, was used to detect the sensitivity of the results of the ratio of incremental cost effectiveness. It was calculated by the expression:

INB = (willingness to pay for PC x Δ of pressure control percentage) / Δ cost of the hypertensive patient care.

Variation in INB results refer to the possible costs that PC may have to the PHS, interpreted as the willingness to pay. Thus, when the variation in the cost refers to negative INB it represents a non-compensatory valuation of PC for investment, and when referring to positive INB values it is a valuation that compensates investment. To determine the valuation of the PC limit, the minimum value considered was one dollar and the maximum value was the cost-effectiveness threshold as recommended, three times GDP per capita of Brazil. Spending with pharmaceutical care was also considered, calculated in the same way as PC cost, this was represented as the willingness to pay for pharmaceutical care after patient discharge [24, 32, 33]. For the CER and ICER sensitivity analysis calculated in this study, Monte Carlo simulation for uncertainties as to the variability of costs and effectiveness was performed. The Monte Carlo analysis used @RISK software version 7, from the Palisade Corporation® 2015 [31, 32]. The cost and effectiveness were calculated for each year and differentiated in baseline, being years of conventional health care of hypertensive patients; year of the pharmaceutical care program; and year after pharmaceutical care, being years in which the results of PC affected the health of hypertensive patients and there was no investment in PC. With the use of *MINITAB* version 17 statistical software, descriptive statistics of the calculated costs and outcomes were performed, summarized in the mean representation, standard deviation, minimum and maximum values and interquartile ranges, and also in histogram and boxplot. In addition to the definition of probability distributions, the data distribution identification analysis by the Anderson-Darling statistical test was performed, which measures how well the data follow a particular distribution; the better the distribution fits the data, the lower this statistic. For this the significance level of 1% was considered.

Descriptive statistics assisted in defining the truncation limits for the curve of cost probability distribution of the mean and standard deviation, considered for both cost and for effectiveness. The box plot and histogram supported the decision to choose the possible results of the distribution identification. The limits of truncation for the minimum percentage of blood pressure control have been established for the years in which the patients were treated by the conventional system in the pre-PC period, being 25%, according to Rosario et al. [12]. For the maximum value the highest percentage of variation on the mean from the years of the period was obtained, and this was added to the value of each year for both the PC and post-PC periods. For the post-PC period, the highest percentage of variation was also used for the calculation of minimum values for each year of the period, and decreased the pressure control percentage each year. The maximum percentage change calculated for the post-PC period was used to calculate the minimum and maximum values during the PC period. For all these values a limit of 1 as a maximum value was established, because it is a percentage. Costs varied according to the characteristic of the distribution of the data analyzed by descriptive statistics, to obtain an annual mean per patient, truncation limits (minimum and maximum cost), and standard deviation [31].

### Ethics

This study has the approval of the Ethics Committee of FCFRP-USP on 10 February 2014, with the release of approval No 004/2014 and protocol CEP/FCFRP No.324, CAAE 21162713.8.0000.5403 (http://plataformabrasil.saude.gov.br). The PC program developed and conducted by CPAFF, had its approval by the Ethics in Research Committee of the Health Center School of Ribeirão Preto Medical Faculty, document 664/07/COORD, CEP/CSE-FMRP-USP-12/12/2007, protocol No. 256/CEP-CSE-FMRP-USP and release 054/2007 and the clinical trial of which this study is part of was recorded in the Brazilian Network for Clinical Trials, registration: RBR-8pchqf; Identification number: UTN: U1111-1172-9577.

## Results

Patients who participated in the PC program were aged between 38–83 years, mean 62.6 ± 10.2 and median of 63.0 years; 95% CI (61.0, 64.4), female, white skin, lower middle class, and moderate cardiovascular risk profile [19]. The annual mean cost of conventional care for the treatment of 104 patients in the PHS was US$ 20,630.63. In the PC period the cost was US$ 20,499.89 and post-PC was US$ 22,350.38. The cost per hypertensive patient was higher in the post-PC period, US$ 214.96 ± 139.70 (Table 2).

**Table 2:** Cost of the public health system with the assistance to hypertensive patients in the follow-up years of the study.

Cost US$		Pre-PC				PC			Post-PC	
	2006		2007	2008		2009	2010	2011		2012
Overall cost	19,644.73	19,505.39			22,741.78	20,499.89	21,676.18		23,350.38	22,039.80
**mean**		**20,630.63**				**20,499.89**			**22,355.45**	
Cost/Patient	188.89 (±122.60)	R$ 187.55 (±118.00)			218.67 (±141.10)	197.11 (±130.20)	208.42 (±134.60)		224.52 (±145.30)	211.92 (±139.30)
**mean**	** **	**198.37 (±127.23)**				**197.11 (±130.20)**			**214.96 (±139.70)**	

PC = Pharmaceutical Care. Values in bold refer to the mean; (Standard Deviation). It emphasizes 2006, 2007 and 2008 were the years in which the patients were not assisted by pharmaceutical care, 2009 was the year that this intervention occurred. The cost-mean per patient calculated for 51 patients (patients with complete data) was multiplied to 104 patients to compound the overall cost. It highlights that the cost of the PC period, 2009, shown in this table does not consider the cost of intervention.

**Fig 1:**
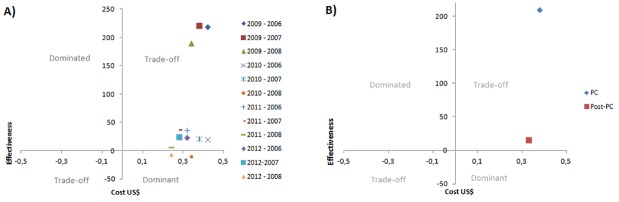
Cost-effectiveness plan. A) Cost-effectiveness plan of pharmaceutical care for years; B) Cost-effectiveness plan of pharmaceutical care for period. By period the mean cost and pressure control percentage of their years was used to compare periods of pharmaceutical care and post pharmaceutical care with the pre-pharmaceutical care period.

The care in the PC and post-PC years was more effective than conventional treatment and also presented a higher cost, except in 2010 and 2012, years that PC was a dominant strategy. When observed per period, can be noted that the impact on blood pressure control during the post-PC period was lower than in the PC period, but also there was lower cost. It is noteworthy that both periods are located in the trade-off quadrant, which highlights the need for incremental cost-effectiveness analysis to aid in decision-making for PC (Fig 1).

All the years after PC presented a CER lower than the years of conventional care for hypertensive patients. In 2010 and 2012, the years in which PC was the dominant strategy when compared to 2008, the ICER was -US$ 30.14 and -US$ 28.12, respectively. The mean CER for the CER_pre-PC_ = US$ 364.65, CER_PC_ = US$ 415.39, and CER_Post-PC_ = US$ 231.14. The incremental cost analysis demonstrated that in the PC period, the ICER was US$ 478.41 and in the post PC period was US$ 42.95 (Table 3).

**Table 3:** Cost effectiveness ratio and incremental cost effectiveness ratio analysis per year and period considering the cost of embedded pharmaceutical care in the cost of the public health system with assistance to hypertensive patients.

COST US$		Pre PC		PC		Post PC	
	2006	2007	2008	2009	2010	2011	2012
	$188.89	$187.55	$218.67	$407.91	$208.42	$224.52	$211.92
Outcome	56% Satisfactory	60% Satisfactory	64% Satisfactory	98%* Satisfactory	98%* Satisfactory	88%* Satisfactory	88%* Satisfactory
**CER**	$337.31	$312.59	$341.67	$416.23	$212.68	$255.14	$240.82
**ICER (2006)**	-	-	-	$521.48	$46.51	$111.35	$71.97
**ICER (2007)**	-	-	-	$579.90	$54.93	$132.04	$87.03
**ICER (2008)**	-	-	-	$556.60	-$30.14	$24.38	-$28.12
Mean cost per period		$198.37		$407.91		$214.96	
Outcomes per period		54.4% Satisfactory		98.2%* Satisfactory		93.0%* Satisfactory	
**CER**		**$364.65**		**$415.39**		**$231.14**	
**ICER**		**-**		**$478.41**		**$42.95**	

CER = Cost Effectiveness Ratio (CER = cost/outcome); ICER = Incremental Cost Effectiveness Ratio (ICER = Δcosts/ Δoutcomes); outcomes = percentage of patients with blood pressure control. The years 2006, 2007, 2008 were considerate as baseline. For analysis of changes of outcomes (percentage of blood pressure control) the Cochran Q test was performed to compare categorical variables. This analysis has tested the hypothesis that modification of this outcome is associated with PC. For this analysis the chi-square distribution for 2 degrees of freedom was considered

* = significant Q Statistics for the Chi-Square > 5.99 (threshold to reject the null hypothesis).

**Fig 2:**
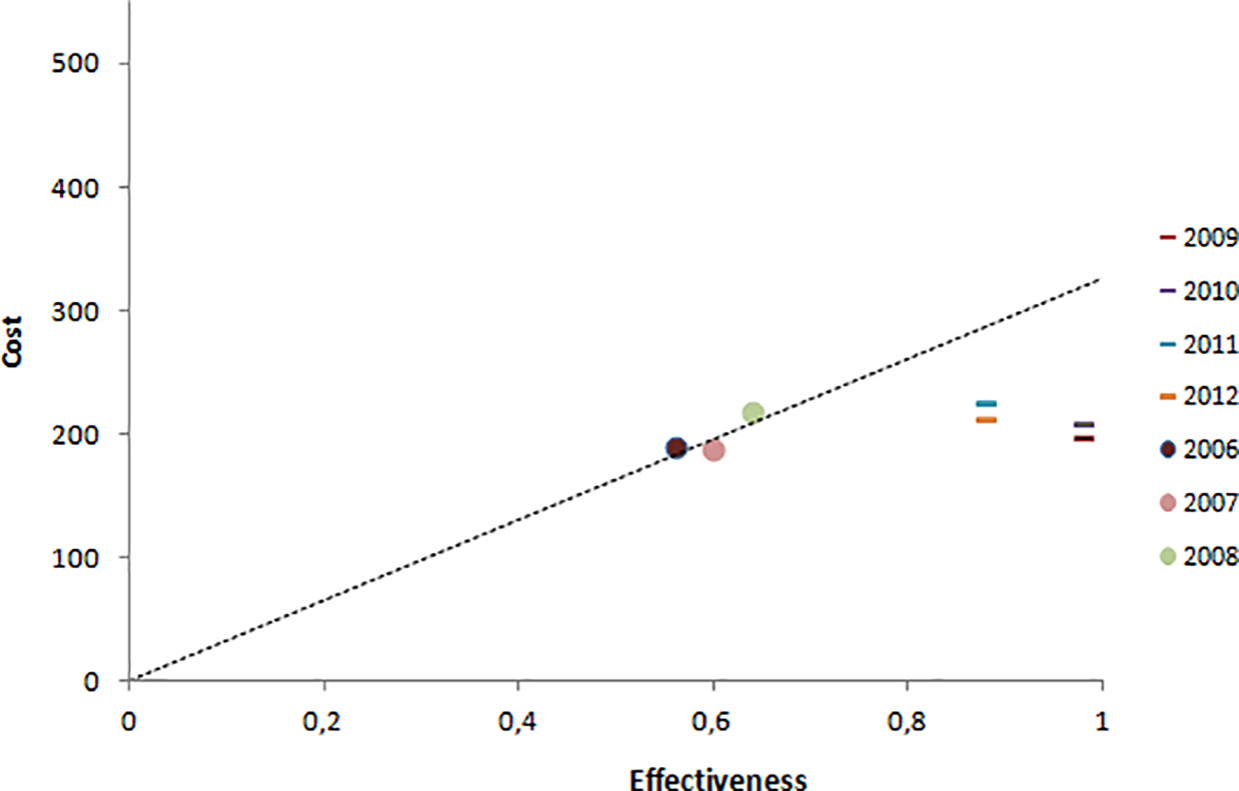
Cost-effectiveness threshold based on the pre-PC cost-effectiveness trend line.

When this study’s baseline data was interpreted as a cost-effectiveness trend line (2006, 2007 and 2008), which represented the cost-effectiveness threshold, it can be seen that the post-PC years (2009, 2010, 2011 and 2012) were below this threshold. It is noteworthy that the cost of PC was not included in 2009, only its impact on the PHS spending and scope of blood pressure control (Fig 2).

**Fig 3:**
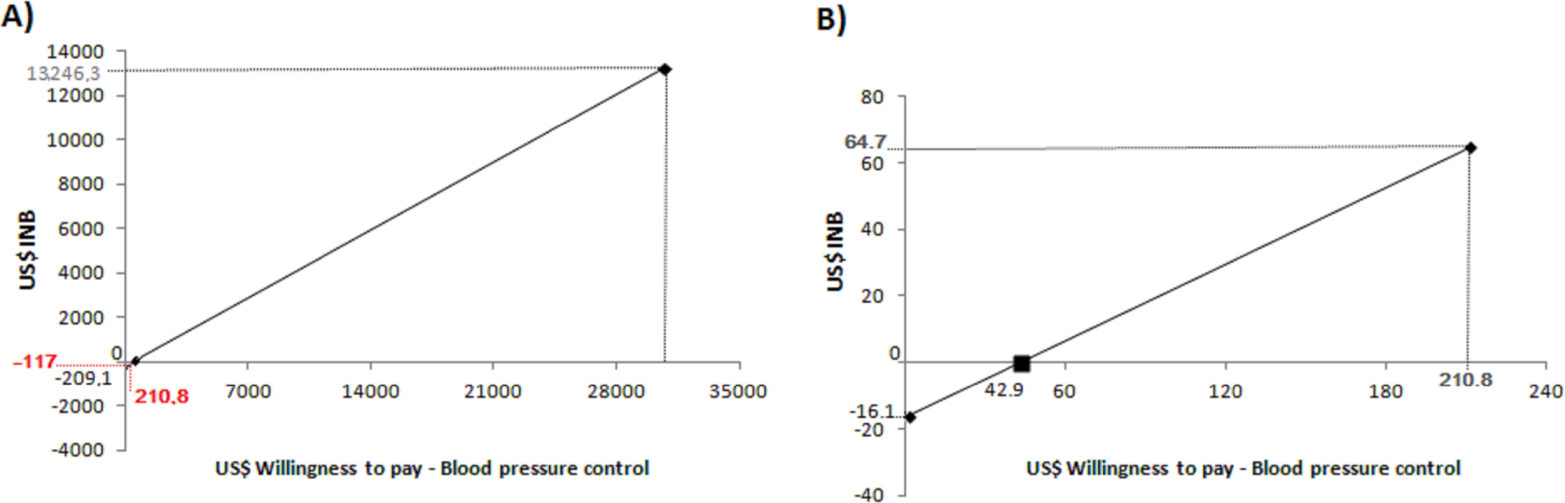
Analysis of the one-way sensitivity performed by the incremental net benefit to pharmaceutical care. INB = Incremental net-benefit. A) Structuring the sensitivity analysis of the incremental net benefit to pharmaceutical care, considering the minimum value of 1 dollar and maximum of 3 x GDP per capita of Brazil used as threshold for the willingness to pay for pharmaceutical care; B) Sensitivity analysis for the incremental net benefit to pharmaceutical care, considering the spending with pharmaceutical care as the willingness to pay for pharmaceutical care after discharge of patients. The maximum value used as a threshold for willingness to pay was the cost of pharmaceutical care calculated in this study. Negative INB values represent a non-compensatory valuation of PC for investment, and positive INB values represent valuation that is compensatory as investment.

Assigning the value of one dollar as a minimum value for the PC willingness to pay to control the patient’s blood pressure in the year, an incremental negative net benefit (INB), equivalent to -US$ 209.01 was obtained. The cost-effectiveness threshold, interpreted as three times the GDP per capita was US$ 30,721.28, considered as the maximum cost value for the willingness to pay for PC to control blood pressure of a patient in the year. Using this value as the willingness to pay for patient’s blood pressure control in the year, an INB of US$ 13,246.38 was obtained. Therefore, the values of -US$ 209.01 to US$ 13,246.38 have represented the range that come out in sensitive values to assign willingness to pay. Thus, the willingness to pay of US$ 478.41, which represented the cost for one more patient with blood pressure controlled in the year, defined the point that the INB value began to be positive. The ICER value of 478.41 has calibrated the threshold and when it was US$ 210.80 (the PC cost per patient) the INB was US$ -117.00 (Fig 3A).

In the post-PC period the sensitivity range was -US$ 16.09 to US$ 13,400.94 (value considered as 3 x GDP per capita as the threshold), and INB began to be positive of US$ 42.95 for the willingness to pay. When placed as willingness to pay in the post-PC period the amount of US$ 210.80 obtained a positive INB of US$ 64.79 (Fig 3B).

According to the cost data distribution identification analysis for each year, lower Anderson-Darling (AD) coefficients predicted the data distribution of more adjusted probabilities were log-logistic for the pre-PC years, AD = 0.170 [p> 0.250]; log-normal for the PC year, AD = 0.324 [p> 0.250]; and Weibull for the post-PC years, AD = 0.197 [p> 0.250]. For the outcomes, triangular distributions were defined.

To perform sensitivity analysis the costs and standard deviation shown in Table 2 were considered. The truncation limits considered for the costs were (7.00–649.00), (8.50–567.10), and (12.80–610.90) for the years 2006, 2007, 2008, respectively; in 2009 it was (13.70–560.70); in the years 2010, 2011, 2012 were (5.90–591.90), (4.80–596.90), and (8.00–556.80), respectively. For the outcome the mean used was the same as that presented in Table 2, there was no need to use standard deviation because it is a triangular distribution. The limits of truncation for the pre-PC period were established for the minimum values of 0.25, which refer to 25% of blood pressure control. For maximum values the highest percentage of variation of the mean was considered, which refers to 2008, being 15%. Therefore, the maximum values calculated for the years of the pre-PC period were 0.65, 0.69, and 0.74 for 2006, 2007 and 2008 respectively. For the post-PC years the minimum and maximum values were calculated by the highest percentage of variation on mean, being 6.4%. Thus the minimum values were 0.92, 0.83, and 0.83; and maximum were 1.00, 0.98, and 0.98 for the years 2010, 2011 and 2012, respectively. For the PC year a minimum value of 0.92 and maximum of 1.00 were established.

**Fig 4:**
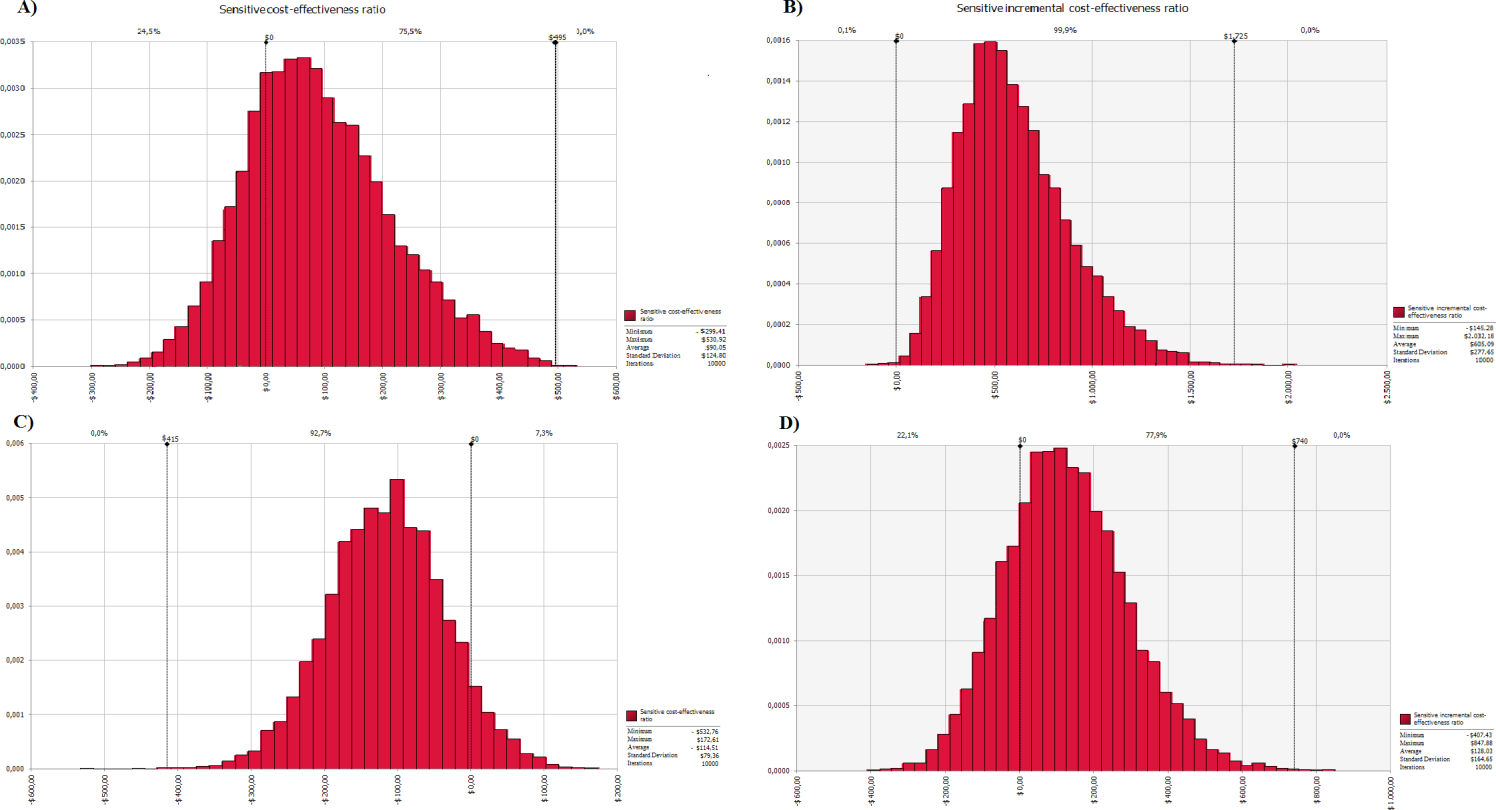
Monte Carlo simulation sensitivity analysis for the ratio of cost effectiveness and the ratio of incremental cost effectiveness in the period. Pre-PC period = baseline; **A)** Monte Carlo simulation sensitivity analysis for ratio of cost effectiveness of pharmaceutical care compared to baseline; **B)** Monte Carlo simulation sensitivity analysis for ratio of incremental cost effectiveness of pharmaceutical care compared to baseline; **C)** Sensitivity analysis by Monte Carlo simulation for ratio of cost effectiveness post pharmaceutical care compared to baseline; **D)** Sensitivity analysis by Monte Carlo simulation for ratio of incremental cost effectiveness for post pharmaceutical care compared to baseline. 10,000 iterations were performed in Monte Carlo simulation to evaluate the variation of the values of the ratio of cost effectiveness and the ratio of incremental cost effectiveness on the variation of costs by pertinent patient to the probability distribution of costs and pressure control percentage of outcome for each baseline year, for the year of pharmaceutical care, and each year after pharmaceutical care. It can be highlighted that after pharmaceutical care there is not the cost of pharmaceutical care for the calculation of the ratios, thus, the ratios reflect the conventional cost for the care of hypertensive patients’ health in the Public Health System as baseline, compared to the result of pharmaceutical care on conventional health costs. Monte Carlo simulations for CER were structured to represent the probability of CER in the PC and post-PC periods being greater than the baseline CER, thus part **A)** reflects the result of CER difference of the PC period minus the baseline CER period and part **C)** reflects the result of the difference of the post-PC CER period minus the baseline CER period.

Fig 4A presented the mean difference between the expected CER of the PC and pre-PC period as US$ 90.05, with a 75.5% chance of being a positive change, which can reach up to US$ 495.00. In post-PC the mean difference of the expected variation of CER post-PC with CER pre-PC was -US$ 114.51 with a 92.7% chance of the results of the CER difference being negative (Fig 4C). The sensitivity of the ICER has shown the mean ICER in the PC period was US$ 605.09, whichpresented 99.9% chance for a positive ICER. Within this variation, the maximum ICER wasUS$ 1,725.00 (Fig 4B). In the post-PC period the mean ICER was US$ 128.03, with a 77.9% chance of the ICER being positive, which can reach US$ 740.00 (Fig 4D).

## Discussion

The mean cost of health care per patient to the PHS in medium-sized cities in Brazil has been estimated at US$ 271.99 per inhabitant/year [35, 36, 37]. The committed cost annually for the treatment of hypertensive patients in the state of São Paulo was US$ 174.24, adjusted for the year 2015 [11, 38, 39]. Our study showed that the mean annual cost/hypertensive patient by conventional health care was US$ 198.37, this is US$ 24.13 more than the mean committed in the PHS for São Paulo state. It is noteworthy that in health care with incorporated PC, in the post-PC period there was an increase of US$ 16.59 per hypertensive patient compared to conventional health care. This can be explained by primary care being more expensive than emergency care to the PHS. The PC program has improved the care profile for hypertensive patients because of increased primary care consultations and reduced emergency consultations [19, 40, 41].

CER calculated in our study has measured that the cost to the health system for blood pressure control per patient was higher with PC management because of immediate investment in this HT in that year. In the post-PC period, the annual mean cost of blood pressure control per patient has reduced by US$ 133 when compared to mean baseline CER. Estimating that the cost to reach effective BP control from 25% to 64% of patients as calculated in our study according to outcome sensitivities [12], the PHS would be paying annually about US$ 272.25 to US$ 580.80 per controlled patient. This highlights that the PC program has impacted directly on costs and outcomes after patients discharge, and was measured by cost-effectiveness analysis, which evidenced it as the dominant strategy. Both ICERs in the PC and post-PC period were below the cost effectiveness threshold of US$ 30,721.28. For adult hypertensive patients who have high cardiovascular risk and are no longer in preventive care, the mean annual cost for blood pressure control reaches US$ 360.51, 89.1% more than the incremental cost post-PC. Most parts of these costs are related to drugs for blood pressure control and cardiovascular risk reduction [42].

Sensitivity analysis for cost effectiveness presented a lower cost trend to reach the blood pressure control of hypertensive patients when patient's care managed by PC was inserted. Therefore, the chance of INB to be negative was 1.6%, which presented a 98.4% chance for PC to be cost effective in accordance with the provision that the PHS would have to invest, until the cost-effectiveness threshold of US$ 13,455. In the post-PC period, the amplitude of INB decreased to US$ 11,841.83, however there was the percentage of 99.8% of PC to be cost effective in relation to the provision that the PHS had to pay for one more patient with blood pressure controlled in the year. According to the PC cost calculated as willingness to pay for PC, despite INB being negative for PC calculated cost in the PC period it was positive in the post-PC period, which meant PC was cost effective in the long-term to the PHS because of the important impact that the patients conduct care to achieve better blood pressure control rates. This fact was also reported in the Rodrigues’s study [43], which calculated the cost of US$ 438.81 per hypertensive patient/year and showed that PC was cost-effective compared to conventional health care.

The cost engaged in PC reached 0.42% of the cost-effectiveness threshold. The literature has shown that this percentage can reach up to 45% of the cost effectiveness threshold value when analyzing PC monitoring of various morbidities [44]. Thus, using this percentage in the cost effectiveness threshold established in our study, it appears that the cost to the PHS would have as an investment in PC by one more patient with blood pressure controlled in the year reach US$ 6,030.42 in the worst scenario. However, even with this increase, PC would be a cost effective strategy because it would still be below the cost effectiveness threshold. In addition, it highlights that PC programs aimed at hypertensive patients can be an important strategy for the PHS to optimize hypertension care. The PHS invests US$ 1,261.68 in antihypertensive drugs annually for patients with controlled pressure; this is almost three times higher than the value assigned to the PC period and almost six times the post-PC period [45, 46].

Monte Carlo sensitivity analysis evidenced that the chances of 75.5% and 92.7% of CER with PC management is less than conventional care. It meant that even with changes in outcomes and costs, the cost of health care for blood pressure control achieved tended to be lower when hypertensive patients had PC assistance, as has also been noted the literature [47].

Additionally, the chances of PC to present negative ICER and to be a dominant strategy was minimal in the PC period, but in the post-PC period it was a 22.1%. It is noteworthy that the largest ICER for PC in both PC and post-PC periods was below the threshold of three times GDP per capita. Furthermore, PC would still be cost effective when considered the maximum ICERs, calculated by Monte Carlo analysis when compared to maximum values for INB, US$ 13,246.38 in the PC period, and US$ 11,841.83 in post-PC as the cost-effectiveness thresholds. In addition, it would represent less than 50% of this cost effectiveness threshold, 15.34% and 7.16% for PC and Post-PC period, respectively. When comparing the higher ICER value calculated by sensitivity analysis, presented in the literature, it still would be lower, representing 33.70% and 14.06% of the threshold of US$ 6,030.42 in the PC and post-PC periods, respectively [44].

We are aware that the number of patients with complete data and included in the analysis was lower than the number enrolled due to missing data for all the years. However, this number did not affect the conclusion of this study because it was performed sensitivity analysis by Monte Carlo simulation, which predicted the variation of costs and outcomes in the outcome of the reasons of cost effectiveness and incremental cost effectiveness, and, even with the worst possibilities of variation, PC presented ICER below 50% of the cost effectiveness threshold. As a result, we believe our findings are robust.

Considering the costing and analytical design of this study, it is expected that this study will subsidize and promote other pharmacoeconomic studies that promote comparison between PC and other HTs for SAH care, and foster robust discussions about this subject. In addition, we strive for these results to assist scientific and academic development because the literature has been poor in original results about cost-effectiveness and PC for hypertension, which also was a limitation for this study. Also, our study can be important to subsidize meta-analyzes in the area, which would increase the decision-making power for investment and would aid in health systems planning for hypertensive patients care, promoting the improvement in indicators and reducing morbidity and mortality caused by SAH.

## Conclusion

Direct cost analysis presented PC as a strategy able to reduce PHS spending on hypertensive patient care. After patients discharge from the PC program, it was inherent that expenditure of the PHS increased because of the improvement in the profile care of hypertensive patients. Furthermore, the annual cost of pressure control increased in the year of investment in PC, but after patient discharge from the PC program the cost for blood pressure control was less than in conventional health care. In addition, the incremental cost for one more patient with blood pressure control per year was lower in the long term with PC management than conventional health care. This highlights that considering the PC investment cost in the short-term, the incremental cost was less than the cost effectiveness threshold, even in the worst scenario. In addition, according to the likely changes of costs and outcomes, there was no chance for the largest calculated incremental cost to overcome the cost effectiveness threshold. It meant that the PC program was a cost effective strategy for hypertensive patient’s care in the PHS when compared with conventional care.

## Supporting information

S1 Appendix Anonymized version DATA. Data from: Long-term economic evaluation of Pharmaceutical Care for patients with systemic arterial hypertension. 2016. 122L. Dissertation (Master). School of Pharmaceutical Sciences of Ribeirão Preto–University of Sao Paulo, Ribeirão Preto, 2016. Available:< www.teses.usp.br/teses/…/Dissertacao_corrigida_simplicado.pdf>.
(PDF)

S2 Appendix Chart 1—Description for calculating the cost of pharmaceutical care. Cazarim MS, Nunes AA, Pereira LRL. Cost-consequence analysis of Pharmaceutical Care program for systemic arterial hypertension in the public health system in Brazil. Braz. J. Pharm. Sci. 2017;53(3):e00217. doi: 10.1590/s2175-97902017000300217.
(PDF)
