# Supplementary material for: Cost-effectiveness analysis of pharmaceutical care for hypertensive patients from the perspective of the public health system in Brazil
Source: PLoS One. 2018 Mar 6;13(3):e0193567. doi: 10.1371/journal.pone.0193567 (PMC5839560; doi:10.1371/journal.pone.0193567)
Supplement: S1 Appendix — Data from: Long-term economic evaluation of Pharmaceutical Care for patients with systemic arterial hypertension. 2016. 122L. Dissertation (Master). School of Pharmaceutical Sciences of Ribeirão Preto–University of Sao Paulo, Ribeirão Preto, 2016. Available:< www.teses.usp.br/teses/…/Dissertacao_corrigida_simplicado.pdf>. (PDF) [file pone.0193567.s001.pdf]

UNIVERSIDADE DE SÃO PAULO

FACULDADE DE CIÊNCIAS FARMACÊUTICAS DE RIBEIRÃO PRETO

Avaliação econômica em longo prazo da atenção farmacêutica para pacientes com hipertensão arterial sistêmica

Maurílio de Souza Cazarim

Ribeirão Preto
2016

UNIVERSIDADE DE SÃO PAULO

FACULDADE DE CIÊNCIAS FARMACÊUTICAS DE RIBEIRÃO PRETO

Avaliação econômica em longo prazo da atenção farmacêutica para pacientes com hipertensão arterial sistêmica

Dissertação de Mestrado apresentada ao Programa de Pós-Graduação em Ciências Farmacêuticas para obtenção do Título de Mestre em Ciências.

Área de Concentração: Medicamentos e Cosméticos.

Orientado: Maurílio de Souza Cazarim

Orientador: Prof. Dr. Leonardo Régis Leira Pereira

Versão corrigida da dissertação de mestrado apresentada ao Programa de Pós-Graduação em Ciências Farmacêuticas em 19/02/2016. A versão original encontra-se disponível na Faculdade de Ciências Farmacêuticas de Ribeirão Preto/USP.

Ribeirão Preto
2016

CAZARIM,
M. S.

Avaliação econômica em longo prazo da atenção farmacêutica para pacientes com hipertensão arterial sistêmica

Espaço de **2,5 cm** reservado para etiqueta de localização da biblioteca

MESTRADO

FCFRP/USP

2016

AUTORIZO A REPRODUÇÃO E DIVULGAÇÃO TOTAL OU PARCIAL DESTE TRABALHO, POR QUALQUER MEIO CONVENCIONAL OU ELETRÔNICO, PARA FINS DE ESTUDO E PESQUISA, DESDE QUE CITADA A FONTE.

CAZARIM, Maurílio de Souza

Avaliação econômica em longo prazo da atenção farmacêutica para pacientes com hipertensão arterial sistêmica. Ribeirão Preto, 2016.
122p.; 30cm.

Dissertação de Mestrado, apresentada à Faculdade de Ciências Farmacêuticas de Ribeirão Preto/USP – Área de concentração: Medicamentos e Cosméticos.

Orientador: Pereira, Leonardo Régis Leira.
1.Atenção Farmacêutica 2.Hipertensão 3.Custos e Análise de Custo 4.Cadeia de Markov 5.Avaliação de Custo-Efetividade.

FOLHA DE APROVAÇÃO

Maurílio de Souza Cazarim

Avaliação econômica em longo prazo da atenção farmacêutica para pacientes com hipertensão arterial sistêmica

Dissertação de Mestrado apresentada ao Programa de Pós-Graduação em Ciências Farmacêuticas para obtenção do Título de Mestre em Ciências.

Área de Concentração: Medicamentos e Cosméticos.

Orientador: Prof. Dr. Leonardo Régis Leira Pereira

Aprovada em: ____/____/____

Banca Examinadora

Prof. Dr. ____

Instituição: _________________________ Assinatura:____________________

Prof. Dr. ____

Instituição: _________________________ Assinatura:____________________

Prof. Dr. ____

Instituição: _________________________ Assinatura:____________________

DEDICATÓRIA

Dedico esta obra, primeiro lugar, a Deus pela sua criação, benevolência e o Amor incondicional que dedica a todos nós. Aos amigos espirituais sempre dispostos a nos ajudar com tamanha responsabilidade, amor e caridade. Aos meus pais José Augusto Cazarim e Juscilene de Souza Cazarim pelo o exemplo de amor, bondade, humildade e empatia; qualidades que guiaram sempre minhas condutas e proporcionaram-me estar caminhando em bons passos. Ao meu irmão Fábio de Souza Cazarim pelos exemplos e ensinamentos, por dispor sempre de um grande amor fraternal. A Estael Cruz por ser a companheira, amiga, parceira, confidente e por aliviar minhas tristezas sempre de prontidão com um sorriso me conduzindo a alegria da vida. A todos aqueles que têm procurado estruturar a saúde de forma humanitária e coesa, não desamparando aqueles que precisam de cuidados e fornecendo-lhes melhores perspectivas.

AGRADECIMENTOS

A agência financiadora Fundação de Amparo à Pesquisa do Estado de São Paulo (FAPESP) pelo fomento a pesquisa com o auxílio da bolsa de pesquisa (Mestrado - Fluxo Contínuo, processo nº 2014/02087-9) e reserva técnica para o desenvolvimento deste estudo.

Ao Conselho Nacional de Desenvolvimento Científico e Tecnológico (CNPq) por anteriormente à FAPESP auxiliar este estudo com a bolsa de pesquisa.

A Universidade de São Paulo e a Faculdade de Ciências Farmacêuticas de Ribeirão Preto, pela estrutura de ensino e por fornecerem todo o suporte necessário para a realização deste trabalho.

À Faculdade de Economia, Administração e Contabilidade de Ribeirão Preto pela capacitação a mim ofertada para o desenvolvimento deste trabalho.

Ao Centro de Pesquisa em Assistência Farmacêutica e Farmácia Clínica (CPAFF) pelas condições de estudo e estrutura oferecidas ao desenvolvimento deste trabalho.

Aos membros e amigos do CPAFF por fazerem dessa jornada um caminho mais ameno e colaborarem com a construção do conhecimento em conjunto.

Aos professores responsáveis pelas disciplinas por mim cursadas na pós-graduação pela acessibilidade e contribuição ao ensino.

Aos funcionários da Secretaria Municipal de Saúde de Ribeirão Preto, em especial à Darlene Mestriner, Lúcia Helena, Daniel do DACA e Marcelo da Informática e Estatística.

Aos amigos Tiago, Lauro, João Paulo, Lucas, Fontoura, André Baldoni e Camilo, agradeço a parceria, auxílio, momentos de descontração que certamente foram influentes para o resultado final deste trabalho.

Aos funcionários do Programa de Pós-Graduação em Ciências Farmacêuticas da Faculdade de Ciências Farmacêuticas de Ribeirão Preto, Eleni Angeli, Rafael Braga, Henrique e Rosana Florêncio pela paciência e eficiência em auxiliar-me.

Á professora Drª. Renata Fonseca Vianna Lopes por deixar sempre as portas abertas ao ensino, por ser um dos exemplos de docentes em que me espelho e por contribuir de forma humana e eficiente ao ensino.

Aos meus pais por representarem o amor, a humildade e a segurança para eu prosseguir neste caminho.

A Estael pela paciência, companheirismo, dedicação e por sempre estar disposta a auxiliar-me.

À minha família por sempre acreditarem em mim e serem a fonte inesgotável de confiança, carinho e afeto.

Aos amigos distantes que compreenderam os momentos ausentes e permaneceram sempre ao meu lado, dando-me forças para continuar a caminhada.

Aos amigos de Ribeirão Preto por trazerem o afago de uma conversa e o acolhimento nos momentos difíceis, em especial ao Marcelo e Valquíria por gentilmente acolher-me sempre que preciso.

Aos amigos Lucas Pinheiro, Felipe Vilela, Davi La gatta, Mariza e Mike por serem especiais e fazerem me sentir mais perto de casa, mesmo a 640 km de distância.

Aos amigos Camila, Leandro, Fernanda e Fábio, Thaís e Rafa por serem especiais e companheiros.

Ao Rafinha, JP e Jú por mostrarem um convívio especial e sempre compartilharem momentos de alegria.

Em especial ao meu orientador, Prof. Dr. Leonardo Régis Leira Pereira por me direcionar a um caminho reto e preciso da pesquisa e docência, sempre depositando confiança no meu trabalho, tornando viável a realização desta obra.

"Devemos ter coragem para vencer nossas imperfeições; a ciência é divina, mas ainda é preciso que os cientistas se divinizem."

Isabel Salomão

RESUMO

CAZARIM, M. S. **Avaliação econômica em longo prazo da Atenção Farmacêutica para pacientes com hipertensão arterial sistêmica**. 2016. 122f. Dissertação (Mestrado). Faculdade de Ciências Farmacêuticas de Ribeirão Preto – Universidade de São Paulo, Ribeirão Preto, 2016.

A hipertensão arterial sistêmica (HAS) é um fator de risco para 9,4 milhões de mortes em todo o mundo. No Brasil, a HAS atribui um custo anual de aproximadamente R$ 969,231,436,00 ao Sistema Único de Saúde (SUS). Alguns estudos têm mostrado uma grande efetividade da Atenção Farmacêutica (ATF) para melhorar os parâmetros clínicos de pacientes com HAS. Diante desse contexto, este trabalho teve o objetivo de desenvolver uma ferramenta econômica considerando custos e desfechos para auxiliar os gestores de saúde a implantar a ATF no SUS e reduzir a morbi-mortalidade ocasionada pela HAS. Para isso, foi conduzido um estudo farmacoeconômico de modelagem com análises de custo-efetividade, custo-benefício e custo-utilidade. Em duas unidades básicas do SUS, em Ribeirão Preto, 104 pacientes foram acompanhados por um programa de ATF durante o ano de 2009. Foram coletados dados clínicos, assistenciais e econômicos referentes aos anos de 2006 a 2012. Os dados foram divididos por períodos, 2006-2008 (pré ATF), 2009 (ATF) e 2010-2012 (pós ATF). A análise econômica foi realizada em cinco etapas: análise direta dos custos, análise indireta dos custos com modelagem de Markov, análise de custo-benefício com obtenção do valor presente líquido (VPL), análise de sensibilidade dos custos, e análise do impacto epidemiológico. O controle pressórico foi alcançado em 54,4%, 98,2% e 93,0% dos pacientes nos períodos pré ATF, ATF e pós ATF, respectivamente. Na análise direta dos custos houve diferença dos custos totais de -R$307,23 e R$4.053,32 e a razão de custo efetividade incremental foi R$1.124,24 e R$100,98, nos períodos ATF e pós ATF, respectivamente. Na projeção por modelagem de Markov houve 46 complicações da HAS e 17 mortes; e 15 complicações e 5 mortes ao longo de dez anos para a assistência a saúde sem a ATF e com a ATF, respectivamente. Houve o custo por paciente de R$ 17,09 / anos de vida ajustados por qualidade e R$ 28,98 por anos de vida ganhos após a alta da ATF. O VPL obtido com o investimento nesta prática farmacêutica foi R$ 3.791.111,13 e a razão benefício - custo (RBC) foi 30,03 (26,74 – 34,28), RBC > 1 com taxa de retorno de investimento de 303%. Conclui-se que a ATF foi capaz de melhorar o controle pressórico e os níveis de colesterol total reduzindo o risco cardiovascular, o que ocasionou a redução de gastos com a saúde e o impacto positivo epidemiológico. Essa análise foi suficiente para fundamentar uma ferramenta farmacoeconômica capaz de auxiliar os gestores na tomada de decisão quanto à implementação da ATF para a redução da morbi-mortalidade ocasionada pela HAS.

Palavras-chaves: Atenção Farmacêutica; Hipertensão; Custos e Análise de Custo; Cadeia de Markov; Avaliação de Custo-Efetividade.

ABSTRACT

CAZARIM, M. S. **Long-term economic evaluation of Pharmaceutical Care for patients with systemic arterial hypertension**. 2016. 122f. Dissertation (Master). Faculdade de Ciências Farmacêuticas de Ribeirão Preto – Universidade de São Paulo, Ribeirão Preto, 2016.

Systemic Arterial Hypertension (SAH) is a risk factor for 9.4 million deaths worldwide. In Brazil, SAH assigns an annual cost of approximately R$969,231,436,00 the Public Health System (PHS). Some studies have shown a great effectiveness of pharmaceutical care (PC) to improve clinical parameters of patients with SAH. In this context, this study aimed to develop an economic tool considering costs and outcomes to help health managers to deploy the PC in the PHS and reduce the morbidity and mortality caused by hypertension. For this, a pharmacoeconomic modeling study of cost-effectiveness analysis, cost-benefit and cost-utility was conducted. Into two basic units of PHS, in Ribeirão Preto, 104 patients were followed for an PC program during 2009. Clinical, healthcare and economic data were collected for the years 2006 to 2012. The data were divided into periods, 2006-2008 pre PC, 2009 PC and 2010-2012 post-PC. The economic analysis was carried out in five stages: Direct cost analysis; indirect cost analysis with Markov modeling; Cost-benefit analysis to obtain the net present value (NPV); Sensitivity analysis of the costs and analysis of epidemiological impact. The pressure control of patients was 54.4%, 98.2% and 93% in the pre PC, PC and post-PC, respectively. In direct cost analysis the difference between the total cost was -R$ 307.23 and R$ 4,053.32; the Incremental Cost Effectiveness Ratio was R$ 1,124.24 and R$ 100.98 for the periods PC and post-PC, respectively. There were 46 complications of hypertension and 17 deaths; and 15 complications and 5 deaths over ten years for health care without the PC and with PC, respectively. The cost per patient was R$ 17.09 / quality-adjusted life years and R$ 28.98 per year of life saved after discharge from the PC. The NPV obtained with the investment in this pharmaceutical practice was R$ 3,791,111.13 and the Benefit-cost Ratio (BCR) was 30.03 (26.74 – 34.28), BCR > 1 and investment return rate was 303%. It was concluded that the PC was able to improve the blood pressure control and total cholesterol level reducing the cardiovascular risk. That meant reduction spending on health and a positive epidemiological impact. This analysis was enough to develop a pharmacoeconomic tool that can assist managers in making decisions regarding the implementation of PC to reduce morbidity and mortality caused by SAH.

Key-Words: Pharmaceutical Services; Hypertension; Costs and Cost Analysis; Markov Chains; Cost-Effectiveness Evaluation.

LISTA DE FIGURAS

**Figura 1.**	Caracterização dos indivíduos diagnosticados com hipertensão arterial sistêmica............................	**11**
**Figura 2.**	Incidência de doenças cardiovasculares de acordo com a faixa etária e o sexo................................	**13**
**Figura 3.**	Fluxograma do desenvolvimento da ferramenta econômica..	**24**
**Figura 4.**	Modelo de fluxo de caixa para a obtenção do valor presente líquido..	**34**
**Figura 5.**	Fluxograma da estratificação do tamanho amostral por indicador a ser analisado............................	**38**
**Figura 6.**	Percentual de risco cardiovascular em dez anos calculado pela escala *ASCVD*................................	**42**
**Figura 7.**	Plano de custo-efetividade da Atenção Farmacêutica por ano...	**51**
**Figura 8.**	Plano de custo-efetividade da Atenção Farmacêutica por período..	**51**
**Figura 9.**	Limiar de custo-efetividade baseado na linha de tendência nos anos pré ATF.................................	**52**
**Figura 10.**	Análise da sensibilidade pelo benefício líquido incremental para a Atenção Farmacêutica............	**53**
**Figura 11.**	Análise da sensibilidade pela disposição a pagar ao desfecho de controle pressórico que a Atenção Farmacêutica alcançou após a alta dos pacientes...	**53**
**Figura 12.**	Árvore de decisão para a aplicação da modelagem de Markov no modelo das doenças cardiovasculares, acidente vascular encefálico, doença isquêmica do coração e doença arterial periférica...	**57**
**Figura 13.**	Árvore de decisão para a aplicação da modelagem de Markov no modelo da insuficiência cardíaca crônica..	**58**
**Figura 14.**	Árvore de decisão para a aplicação da modelagem de Markov no modelo da doença renal crônica	**59**
**Figura 15.**	Projeção logarítmica natural para aos custos da hipertensão calculados na primeira etapa do estudo..	**62**
**Figura 16.**	Fluxo de caixa referente aos dez anos de projeção de custos com a hipertensão e suas complicações	**66**
**Figura 17.**	Gráfico de dispersão do custo com a Atenção Farmacêutica e o valor presente líquido em dez anos..	**70**
**Figura 18.**	Gráfico de densidade de probabilidades para o valor presente líquido...	**71**
**Figura 19.**	Diagrama de Tornado para a influência das variáveis de custo no valor presente líquido................	**72**
**Figura 20.**	Gráfico de tendência do valor presente líquido ao longo de dez anos...	**73**
**Figura 21.**	Gráfico de densidade de probabilidades para o valor presente líquido sem os custos indiretos.........	**74**
**Figura 22.**	Diagrama de Tornado para a influência das variáveis de custo no valor presente líquido na ausência dos custos indiretos..	**75**
**Figura 23.**	Gráfico de tendência do valor presente líquido sem influência dos custos indiretos ao longo de dez anos..	**75**
**Figura 24.**	Gráfico de densidade de probabilidades para o valor presente líquido sem os custos indiretos.........	**76**
**Figura 25.**	Ferramenta farmacoeconômica para auxiliar a tomada de decisão aos gestores de saúde para a implantação da Atenção Farmacêutica...	**79**
**Figura 26.**	Exemplo do funcionamento da ferramenta farmacoeconômica para o auxilio da tomada de decisão	**80**

LISTA DE TABELAS E QUADROS

TABELAS

Tabela 1. Tabela 2x2 para análise epidemiológica........ **36**

Tabela 2. Perfil dos pacientes hipertensos acompanhados pela Atenção Farmacêutica no início do estudo **39**

Tabela 3. Proporção de pacientes com os resultados clínicos satisfatórios para os três períodos de análise dos resultados........ **40**

Tabela 4. Medidas de resumo e dispersão relativas às consultas e ao consumo de medicamentos anti-hipertensivos para os três períodos de análise dos resultados........ **41**

Tabela 5. Custos discriminados por ano e por período para a análise direta de custos........ **47**

Tabela 6. Custos totais para a análise direta dos custos........ **47**

Tabela 7. Análise da razão de custo efetividade e da razão de custo efetividade incremental por ano e período........ **50**

Tabela 8. Custos anuais das complicações da hipertensão e os custos indiretos........ **54**

Tabela 9. Tabela dos custos das complicações da hipertensão quanto a aplicação na projeção de Markov.. **54**

Tabela 10. Taxas e probabilidades calculadas para a incidência/100 de acidente vascular encefálico, doença isquêmica do coração e doença arterial periférica........ **55**

Tabela 11. Taxas e probabilidades calculadas para a prevalência, morte e reincidência /100 de acidente vascular encefálico, doença isquêmica do coração e doença arterial periférica........ **55**

Tabela 12. Taxas e probabilidades calculadas para a incidência /100 e para o nº de óbitos/100 relativas à insuficiência cardíaca e doença renal crônica........ **56**

Tabela 13. Tabela da projeção dos estados de saúde de Markov, apresentando os anos de vida ganhos, anos de vida ajustados pela qualidade e o número necessário para tratar........ **60**

Tabela 14. Custos diretos da hipertensão projetados em dez anos quanto ao tratamento convencional ofertado pelo Sistema Único de Saúde e ao tratamento com o programa de Atenção Farmacêutica **62**

Tabela 15. Projeção dos custos diretos e indiretos para a hipertenção arterial sistêmica e suas complicações no período de dez anos........ **64**

Tabela 16. Tabela de descrição das variáveis de custo para a análise de sensibilidade........ **68**

QUADROS

Quadro 1. Exemplo do modelo de ajuste de doses desenvolvido neste estudo para a comparação e análise do consumo de medicamentos anti-hipertensivos........ **23**

Quadro 2. Descrição para o cálculo dos custos da Atenção Farmacêutica........ **43**

Quadro 3. Descrição dos materiais necessários para estruturar o serviço de Atenção Farmacêutica para o atendimento de 104 pacientes........ **44**

Quadro 4. Descrição dos custos para o cálculo dos custos das consultas no atendimento especializado, pronto atendimento e atendimento básico........ **45**

Quadro 5. Custo de aquisição dos medicamentos anti-hipertensivos pelo município de Ribeirão Preto....... **45**

Quadro 6. Carga tributária descontada do salário do trabalhador no Brasil........ **46**

Quadro 7. Determinação dos custos diretos não médicos........ **46**

LISTA DE ABREVIATURAS E SIGLAS

ACB – Análise de custo-benefício

ACC/AHA - *American College of Cardiology/American Heart Association*

ACE – Análise de custo-efetividade

AH – Anti-hipertensivo

ANOVA – *Analysis of variance*

ASCVD – *Atherosclerotic Cardiovascular Disease*

ATF – Atenção Farmacêutica

AVAQ – Ano de vida ajustado pela qualidade

AVE – Acidente vascular encefálico

AVG – Ano de vida ganho

BLI – Benefício líquido incremental

CEME – Central de Medicamentos

CPAFF – Centro de Pesquisa em Assistência Farmacêutica e Farmácia Clínica

CPMF – Contribuição Provisória sobre Movimentação Financeira

CT – Colesterol total

DAOP – Doença arterial obstrutiva periférica

DAP – Doença arterial periférica

DCV – Doença cardiovascular

DIC – Doença isquêmica do coração

DRC – Doença renal crônica

ECG - Eletrocardiograma

FAEPA – Fundação de Apoio ao Ensino, Pesquisa e Assistência

FCFRP-USP – Faculdade de Ciências Farmacêuticas de Ribeirão Preto, Universidade de São Paulo

FGTS – Fundo de Garantia do Tempo de Serviço

HAS – Hipertensão arterial sistêmica

HDL – *High-density lipoprotein*

IAPs – Institutos de Aposentadoria e Pensões

IBGE – Instituto Brasileiro de Geografia e Estatística

IC – Insuficiência cardíaca

INPS – Instituto Nacional de Previdência Social

LDL – *Low-density lipoprotein*

NNT – Número necessário para tratar

OR – *Odds Ratio*

PAD – Pressão arterial diastólica

PAS – Pressão arterial sistólica

PFT – Problemas farmacoterapêuticos

PIB – Produto interno bruto

PNM – Política Nacional de Medicamentos

PWDT – *Pharmacist`s Workup of Drug Therapy*

RBC – Razão de benefício-custo

RCE – Razão de custo-efetividade

RCEI – Razão de custo-efetividade incremental

REBRATS – Rede Brasileira de Avaliação de Tecnologias em Saúde

REMUME – Relação Municipal de Medicamentos Essenciais

RR – Risco relativo

RRR – Redução do risco relativo

SAMU – Serviço Ambulatorial Móvel de Urgência

SASSOM – Serviço de Assistência à Saúde dos Municipiários.

SBC – Sociedade Brasileira de Hipertensão

SIASUS – Sistema de Informações Ambulatoriais do Sistema Único de Saúde

SIH – Sistema de Informações Hospitalares

SMS-RP – Secretaria municipal de saúde de Ribeirão Preto

SOAP – Subjetivo, objetivo, avaliação e plano

SUS – Sistema Único de Saúde

TG - Triglicerídeos

TIR – Taxa de retorno de investimento

TOM – *Therapeutic outcomes monitoring*

TS – Tecnologia em saúde

UBDS – Unidade Básica Distrital de Saúde

UBS – Unidade Básica de Saúde

VP – Valor presente

VPL – Valor presente líquido

LISTA DE SÍMBOLOS

mL - mililitros

mg - miligramas

mmHg – milímetro de mercúrio

n – número amostral

$Z\alpha/_{2}$ – percentil da normal padrão

Dp – desvio padrão

Δ - variação

Ɛ - precisão

® - registro referência

R$- unidade monetária do real

< - menor que

> - maior que

≤ - menor igual que

≥ - maior igual que

÷ - divisão

× - multiplicação

$^{-}$ - subtração

$^{+}$ - soma

SUMÁRIO

RESUMO........ i
ABSTRACT........ ii
LISTA DE FIGURAS........ iii
LISTA DE TABELAS E QUADROS........ iv
LISTA DE ABREVIATURA E SIGLAS........ v
LISTA DE SÌMBOLOS........ vii
1. INTRODUÇÃO........ 1
2. REVISÃO BIBLIOGRÁFICA........ 4
2.1. Sistema de saúde brasileiro e formação do Sistema Único de Saúde........ 4
2.2. Financiamento do Sistema Único de Saúde........ 5
2.3. Recursos empregados em saúde no Sistema Único de Saúde........ 7
2.4. Atenção Farmacêutica: modelo de prática farmacêutica para o cuidado do paciente........ 9
2.5. Doenças crônicas não transmissíveis: hipertensão arterial sistêmica........ 10
2.6. Doenças cardiovasculares associadas à hipertensão arterial sistêmica........ 12
2.7. Morbidades associadas à hipertensão arterial sistêmica como complicações da doença........ 13
2.8. Medida do risco cardiovascular pela escala *ASCVD*........ 15
2.9. Análises de decisão associada a modelagens para estimativas das complicações de doenças crônicas........ 16
2.10. Avaliação econômica e farmacoeconomia........ 17
2.11. Hipótese do estudo........ 18
3. OBJETIVOS........ 19
3.1. Geral........ 19
3.2. Específicos........ 19
4. CASUÍSTICA E MÉTODOS........ 20
4.1. Desenho do estudo........ 20
4.2. Programa de Atenção Farmacêutica realizado para o cuidado da hipertensão........ 20
4.3. Indicadores clínicos e assistenciais do estudo........ 21
4.4. Estatística e planejamento amostral........ 22
4.5. Análise farmacoeconômica........ 24
4.6. Comitê de ética em pesquisa........ 37
5. RESULTADOS E DISCUSSÃO........ 38
5.1. Perfil dos pacientes hipertensos do estudo........ 38
5.2. Resultados clínicos e assistenciais........ 40
5.3. Determinação e análise dos custos diretos........ 43
5.4. Determinação dos custos das complicações da hipertenção e dos custos indiretos........ 53
5.5. Determinação das probabilidades para a modelagem de Markov........ 55
5.6. Estruturação do modelo de Markov para a projeção das complicações da hipertensão........ 56
5.7. Projeção dos custos para dez anos........ 62
5.8. Obtenção do valor presente líquido atribuído à economia relativa aos gastos com a hipertensão arterial sistêmica e suas complicações........ 65
5.9. Análises de sensibilidade dos custos para o valor presente líquido........ 67
5.10. Impacto epidemiológico da Atenção Farmacêutica quanto ao controle pressórico e as complicações da hipertensão........ 76
5.11. Ferramenta farmacoeconômica........ 78
6. CONCLUSÃO........ 84
7. REFERÊNCIAS........ 86
ANEXOS

1. INTRODUÇÃO

No total de 56 milhões de mortes no mundo em 2012, 38 milhões foram devido às doenças crônicas não transmissíveis. Quase 75% dessas mortes ocorrem em países de média e baixa renda (WHO, 2014b). No Brasil, as doenças crônicas não transmissíveis respondem pelas maiores taxas de morbi-mortalidade e por uma significativa parcela nas despesas com a assistência ambulatorial e hospitalar, correspondendo a 70% dos gastos assistenciais com a saúde no país (OMS, 2003; BRASIL, 2008). Dentre as mais importantes morbidades crônicas não transmissíveis destaca-se a hipertensão arterial sistêmica (HAS) (CIPULLO et al., 2010; WHO, 2013).

A HAS acomete cerca de um terço da população brasileira, alcançando mais de 50% dos indivíduos na terceira idade e 5% das crianças e adolescentes. Sua gravidade relaciona-se ao comprometimento de órgãos alvos, como rins, coração e cérebro (SBC, 2010; WHO, 2014b). Segundo a *World Health Organization* (*WHO*) (2011) a HAS é considerada o principal fator de risco para a ocorrência de outras doenças do sistema circulatório, como as doenças cardiovasculares (DCVs). De modo que o incremento de 10 mmHg na pressão arterial sistólica (PAS) de um paciente com nível pressórico insatisfatório é capaz de aumentar em até 25% o risco do desenvolvimento de DCV (ROUSH et al., 2014).

Em adição, 30% dos hipertensos possuem a pressão arterial dentro dos parâmetros considerados como satisfatórios pela VI diretriz da Sociedade Brasileira de Cardiologia (SBC) (2010), de forma geral ≤ 139 x 89 mmHg, o que representa uma baixa taxa (SBC, 2010, PONTES-NETO et al., 2008). Há estudos que mostram um percentual de controle pressórico abaixo de 20% dentre os pacientes hipertensos no Brasil (JARDIM et al., 2007; ROSÁRIO et al., 2009). Esse quadro tem agravado o índice de mortalidade por DCVs, pois 9,4 milhões das mortes em decorrência das DCVs no mundo tiveram a HAS como fator associado, ou seja, 16,5% das mortes globais no ano de 2010 (LUNA, 2009; WHO, 2013; WHO, 2014a).

Em 2008, 17,3 milhões de pessoas morreram em decorrência das DCVs, estando os maiores índices de mortes relacionados à doença isquêmica do coração (DIC) e acidente vascular encefálico (AVE), com respectivamente, 7,3 e 6,2 milhões de óbitos. Em 2012 as DCVs foram as morbidades que lideraram a taxa de óbitos no mundo, causando 17,5 milhões de mortes, ultrapassando o câncer (8,2 milhões) e o diabetes (1,5 milhões) (WHO, 2014a). Em agravo a este contexto, as estimativas para o número global de óbitos por DCVs é de 23,3

milhões para 2030. Adicionalmente, ressalta-se que as DCVs são uma das principais causas de incapacidade ocupacional (WHO, 2013; WHO, 2014a).

A consequência deste quadro da saúde no Brasil foi apresentada pela VI diretriz da SBC (2010), a qual traduz que cerca de 1,2 milhões das hospitalizações no Brasil estão vinculadas às DCV. Tal realidade remete ao fato que a HAS têm custado cerca de um bilhão e oitocentos mil reais aos sistemas de saúde no Brasil. A parcela representativa para o Sistema Único de Saúde (SUS) foi de aproximadamente um bilhão de reais, o que projetando para o ano de 2015 representou 0,2% do Produto Interno Bruto (PIB) nacional desembolsados somente com a HAS. Sendo que, no mesmo ano, os gastos totais com a saúde no país foram equivalentes a aproximadamente 7% do PIB (PIOLA et al., 2009; SBC, 2010).

Esse contexto delineia uma realidade na qual o investimento para a melhoria do acesso à saúde e para o tratamento no caso da HAS não tem apresentado a efetividade esperada (ROSÁRIO et al., 2009; SBC, 2010). O que é agravado pelo subfinanciamento do SUS e as dificuldades em melhorar a utilização de recursos de saúde. Com isso o SUS tem procurado alternativas para contornar o panorama da HAS no Brasil, traçando estratégias e propostas de cuidado que promovam a melhoria dos resultados clínicos e do cuidado do paciente, e buscando tecnologias em saúde (TS) que sejam capazes de serem incorporadas nos serviços de saúde preexistentes (FINKELMAN, 2002; SBC, 2010). Uma TS que tem apresentado efetividade no controle de doenças crônicas como a HAS (ARAÚJO, UETA, FREITAS, 2005; AGUWA, UKWE, EKWUNIFE, 2008; LYRA, MARCELLINI, PELÁ, 2008; PENAFORTE, 2011), e que tem sido capaz de otimizar os recursos empregados na área da saúde é a Atenção Farmacêutica (ATF) (MACHADO et al., 2007; VON MUENSTER et al., 2008; CARTER et al., 2009a; CARTER et al., 2009b).

A ATF é um modelo de prática profissional cujo farmacêutico promove o cuidado do paciente por meio de consultas para o acompanhamento farmacoterapêutico. Alguns estudos têm mostrado que a ATF tem sido efetiva não somente no controle da pressão arterial, mas também na redução de complicações de saúde ocasionadas pela HAS (OPAS, 2002; DE CASTRO & CORRER, 2007; PEREIRA & FREITAS, 2008; AGUWA, UKWE, EKWUNIFE, 2008; LYRA, MARCELLINI, PELÁ, 2008; ZUBIOLI et al., 2013). Conforme evidenciado por Souza et al. (2007), essa prática é capaz de reduzir em 20 mmHg PAS e 10 mmHg pressão arterial diastólica (PAD) em pacientes hipertensos. Esses resultados clínicos e a aptidão do farmacêutico diante o conhecimento da farmacoterapia permitiram que esse

profissional fosse inserido na linha de cuidado multiprofissional dos pacientes hipertensos da VI diretriz da SBC (SBC, 2010).

Contudo, as políticas públicas de saúde não possuem uma diretriz ou um modelo consolidado para o planejamento da ATF no sistema público de saúde, o que faz com que a ATF seja incipiente no Brasil. Em adição, é possível destacar alguns estudos que avaliam a efetividade da ATF e tentam justificar o encorajamento de tal prática no SUS por meio da farmacoeconomia (MOTA, 2003; BORGES 2008; RASCATI, 2010; BORGES et al., 2011; OLIVEIRA, 2013). Entretanto, esses resultados não tem sido suficientes para subsidiar o planejamento e encorajar essa prática no SUS, o que pode ser justificado uma vez que a otimização dos recursos e o retorno monetário do investimento não são imediatos. Em adição, não existe um modelo de avaliação que seja capaz de fundamentar em termos econômicos a aplicação de tecnologias em saúde como essa, muito menos, capaz de consolidar economicamente o retorno desse investimento (ZUCCHI & FERRAZ, 2010).

2. REVISÃO BIBLIOGRÁFICA

2.1. Sistema de saúde brasileiro e formação do Sistema Único de Saúde

Atualmente o Brasil possui o sistema de saúde composto pelo setor privado e público, sendo o sistema público chamado de Sistema Único de Saúde (SUS). O processo de formação do SUS foi se consolidando na década de noventa, sendo ele idealizado com a reforma sanitária. As bases dos princípios doutrinários e organizacionais do SUS foram oriundos das propostas sanitaristas. Nesse sentido, é possível destacar duas políticas de saúde implementadas que foram relevantes em todo o processo: as Ações Integradas de Saúde, vigentes no período 1983-1987, foi a proposta de integração e racionalização dos serviços públicos de saúde e da articulação destes com a rede conveniada e contratada, o que iria compor um sistema unificado, regionalizado e hierarquizado com referência e contra referência para o atendimento; e a proposta do Sistema Unificado e Descentralizado de Saúde - SUDS, a qual conveniava o sistema vigente, Instituto Nacional de Assistência Médica da Previdência Social – INAMPS, com os governos estaduais, estruturando o princípio da universalização do atendimento, período 1988-1989. Um fato importante precedente ao SUS e decisivo para o projeto de construção da política do SUS foi a VIII Conferência Nacional de Saúde, em 1986, pois foi o marco da participação da comunidade e dos técnicos na discussão de uma política setorial para aprovação da universalização da saúde e do controle social (ALMEIDA, 1995; FLEURY & OUVERNEY, 2008).

A atual política pública de saúde no Brasil se concretizou a partir da Constituição Federal, promulgada em 1988. Desde então foi definida a saúde como dever do Estado, o qual deve geri-la de forma universal, integrada e descentralizada, garantindo a participação da sociedade. Anterior ao SUS, a saúde se presumia na assistência prestada inicialmente pelos Institutos de Aposentadorias, os IAPs, depois pelo Instituto Nacional de Previdência Social - INSS e na última fase pelo Instituto Nacional de Assistência Medica da Previdência Social - INAMPS, sistemas restritos a parcela da população que contribuía com parte do seu salário para ter o direito à saúde (ALMEIDA, 1995).

O Art. 194 da Constituição Federal de 1988 definiu a saúde como política pública do Estado e garantiu a Seguridade Social, no intuito de prover os direitos à saúde, à previdência e à assistência social. A seguridade Social quanto política foi instaurada com a Assembleia Nacional Constituinte de 1987/88, e foi propulsora da Lei Orgânica da Saúde (Lei

8080/1990) que incorporada à Lei Complementar da Saúde (lei 8142/1990), a Lei Orgânica da Seguridade Social (lei 8212/1991) e à Lei Orgânica da Assistência Social (lei 8742/1993) constituíram as bases do regimento legal para a formação do SUS.

Diante deste contexto, surgiu um sistema de saúde universal, proposto a atender toda a população, e atuar de forma equânime e integralizada. Esse sistema ainda incorpora como campo de ação governamental para a saúde, a alimentação, a moradia, o saneamento básico, o meio ambiente, o trabalho, a renda, a educação, o transporte, o lazer e o acesso aos bens e serviços essenciais. Na atualidade, esse sistema dinâmico tem sofrido algumas mudanças e tem enfrentado algumas dificuldades, dentre as principais destaca-se aquelas relacionadas aos aspectos financeiros (FLEURY & OUVERNEY, 2008).

2.2. Financiamento do Sistema Único de Saúde

A legislação brasileira, por meio da Constituição Federal de 1988, passou a destacar o planejamento como "pressuposto de uma gestão fiscal responsável", exigindo que o gestor público planejasse suas ações, de modo transparente, visando ao aumento da eficiência da ação governamental e ao alcance dos resultados. Porém, a Constituição não definiu percentuais de contribuição e tampouco a participação de cada esfera. Esse processo veio se estruturando desde as leis orgânicas, como alternativas prioritárias, e o governo instaurou medidas provisórias como a Contribuição Provisória sobre Movimentação Financeira - CPMF para a arrecadação e o auxílio do financiamento da saúde (FINKELMAN, 2002).

No final da década de 90, o financiamento do SUS começou a ser estruturado com bases legais para propiciar o planejamento adequado da saúde. A Emenda Constitucional nº 29 de 2000 foi importante neste processo, pois fundamentou a articulação frente às questões financeiras do sistema de saúde, estipulando a participação e a inserção da União, dos estados e dos municípios de forma gradativa no financiamento do SUS. Sendo assim, foi possível delinear a disponibilidade e aplicação dos recursos públicos para a sustentação e melhoria da qualidade de um sistema de saúde universal, integral e equânime (FINKELMAN, 2002; BRASIL, 2000).

Contudo, toda a reestruturação para o planejamento e financiamento do SUS previa também a estimulação da participação da iniciativa privada em investimentos de interesse público. Esse ponto foi fundamental para que a Emenda Constitucional nº 29/2000 postergasse, pois o capital privado teve o foco nos interesses de crescimento próprio. Fato

que, juntamente a uma fraca economia capitalista de Estado, culminou em dificuldades administrativas afetando diretamente as políticas públicas do Estado. Sendo assim, a Emenda nº 29/2000 acabou por vigorar com a Lei Complementar nº 141 em 2011, oficializando a forma do repasse financeiro. No entanto, o atraso na estruturação e na consolidação financeira do SUS acarretou e, até hoje acarreta, em consequências ruins para o desempenho do sistema de saúde brasileiro (BRASIL, 2011; TEIXEIRA & TEIXEIRA, 2003).

De certa forma, toda a problemática das questões financeiras envolvidas no SUS pode ser resumida em quatro temas fundamentais: a destinação do capital, aproveitamento dos recursos, incapacitação dos gestores, e o subfinanciamento. Esses quatro itens foram capazes de consolidar uma situação que se estende por anos e que, atualmente, tende a ser solucionada pela Lei Complementar nº 141 de 2011. Essa Lei traz ainda em seu contexto, a reforma fiscal para a saúde, reestabelecendo o planejamento entre as três esferas político-administrativas (BRASIL, 2011; FINKELMAN, 2002).

A destinação do capital é um problema atribuível à inconsistência fiscal do Brasil. A arrecadação fiscal é oriunda basicamente de três formas: o orçamento fiscal, o orçamento das estatais e o orçamento da seguridade social. O orçamento fiscal é composto pelos impostos, sendo o mais relevante deles o imposto de renda, cujos gastos são direcionados ao conjunto de políticas públicas. A seguridade social na Constituição de 1988 tem sido consolidada pelas políticas de saúde e pela previdência e assistência social. Há também o orçamento próprio do Estado composto de recursos orçamentários destinados exclusivamente às políticas públicas. Essa estrutura forma um modelo chamado de vinculação de receitas. Parte dos recursos arrecadados pela União é distribuída nos setores públicos, sendo uma parcela destinada à saúde. Porém, parte desta parcela se direciona diretamente para o sistema de saúde, pois alguns determinantes sociais são interpretados como indicadores de saúde e acabam requerendo a outra parte. Dessa forma, os recursos que são destinados às ações típicas de saúde do SUS se tornam insuficientes para a vitalidade do sistema (BRASIL, 2011).

O aproveitamento dos recursos torna-se um grande problema devido à forma como esse investimento chega à saúde. Em todo o processo ocorre uma burocratização que torna inconsistente todo o planejamento realizado. Muitas vezes os recursos chegam com atraso à data prevista no planejamento, não sendo empregado da mesma forma a ser otimizado, havendo a inconsistência de todo seu planejamento pela instabilidade da economia e a inflação. Dessa forma, pode ocorrer a perda do recurso nas situações em que esse processo

predestina o total emprego dos valores, o que acaba por propiciar o mau uso dos recursos e desinvestimentos quando não utilizado. Adicionalmente, quando não ocorre a utilização, o recurso retorna para a União e não é necessariamente redirecionado para o setor da saúde, mas, distribuído para os gastos públicos. Em suma, essa problemática acaba influindo no planejamento administrativo do SUS (BRASIL, 2011).

A incapacitação dos gestores pode ser considerada um efeito de sucumbência da descentralização, que é um dos princípios organizacionais do SUS, estruturado a partir das normas operacionais e que tem se tornado o foco de um problema de ordem financeira, em decorrência da deficiente articulação entre o processo de automação das esferas político-administrativas. Os municípios foram adquirindo maior autonomia para o gerenciamento da saúde, com isso o estado foi se afastando e tornando-se mais administrativo que gestor. Porém, houve o desequilíbrio entre o eixo estado-município e muitos municípios têm deflagrado o caos na gestão. Há tentativas de amenizar esse problema, que são realizadas por meio dos Contratos Administrativos da Gestão Pública, os COAPS, que por sua vez incumbe a cada esfera as suas responsabilidades administrativa e gerencial, auxiliando a comunicação entre elas quanto à gestão, principalmente no que se refere aos repasses financeiros. Isso tem como resposta a retomada dos espaços na gestão pelo estado, porém ainda tem sido insuficiente para contornar o problema (BRASIL, 2011).

O subfinanciamento pode ser considerado o maior problema do SUS, pois o Brasil investe cerca de 3,5% do seu PIB, sendo que teria que investir o dobro. O que seria cerca de 7,1% do Estado acrescido a 3,0% do setor privado, totalizando em média 10% de investimento. Na realidade se tem 6,5%-7,0% no total, pois os 3,5% são complementados por aproximadamente 3,0% do setor privado. Ao comparar com o setor suplementar, que chega a investir cerca de R$1.650,00 por beneficiário ano, o financiamento do sistema de saúde deveria aumentar em 115% para se igualar. Quando comparado aos sistemas de saúde estruturados em outros países, o Brasil deveria aumentar o investimento em saúde para uma taxa de 5,5% do PIB. Todavia, ressalta-se que essa questão é extremamente influenciável pela robustez da economia do país (BRASIL, 2011).

2.3. Recursos empregados em saúde no Sistema Único de Saúde

Os repasses financeiros no SUS ocorrem por forma de blocos de financiamento que são constituídos por componentes conforme as especificidades de suas ações e dos serviços

de saúde pactuados, o que contribui para a implementação de ações e serviços e, consequentemente, para a organização e eficiência do SUS. Os recursos financeiros são transferidos em conta única e específica para cada bloco, que se dividem em: Atenção Básica, Atenção de Média e Alta Complexidade, Vigilância em Saúde, Assistência Farmacêutica, Gestão do SUS, e Investimentos na Rede de Serviços de Saúde (OLIVEIRA, BERMUDEZ, CASTRO, 2007; BRASIL, 2011).

A Assistência Farmacêutica compõe o cenário de assistência à saúde pelo planejamento e gerenciamento dos insumos farmacêuticos (OLIVEIRA, BERMUDEZ, CASTRO, 2007). Insere-se no SUS como uma política gerencial para garantir o acesso aos medicamentos com qualidade e segurança, sendo esses considerados essenciais à promoção da saúde. Esse setor foi se constituindo desde 1971 com a instituição da Central de Medicamentos (CEME), a qual tinha a missão de fornecer os medicamentos à população sem condições econômicas para adquiri-los. A CEME possuía gestão centralizada tanto para a aquisição, quanto para a distribuição de medicamentos (BRASIL, 1971). Frente a inúmeros problemas, principalmente os decorrentes da logística da centralização dos processos de programação, aquisição e armazenamento que não correspondiam à realidade da demanda dos serviços de saúde dos estados e dos municípios foi publicada, em 1998, a Política Nacional de Medicamentos (PNM). A PNM reorientou a Assistência Farmacêutica na descentralização da gestão, na promoção do uso racional dos medicamentos, na otimização e eficácia do sistema de distribuição no setor público e no desenvolvimento de iniciativas que possibilitem a redução nos preços dos produtos (BRASIL, 2002).

A PNM foi um instrumento norteador de todas as ações no campo da política de medicamentos no país, modificando uma política que era basicamente de suprimento, para uma configuração assistencial mais ampla para o sistema de saúde. Essa reconfiguração na gestão dos insumos farmacêuticos no Brasil foi se moldando como Assistência Farmacêutica (BRASIL, 1998), a qual foi ser aprovada como política pela Resolução nº 338, de 6 de maio de 2004, do Conselho Nacional de Saúde, e foi definida como:

> Conjunto de ações voltadas à promoção, proteção e recuperação da saúde, tanto individual como coletiva, tendo o medicamento como insumo essencial e visando ao acesso e ao seu uso racional. Este conjunto envolve a pesquisa, o desenvolvimento e a produção de medicamentos e insumos, bem como a sua seleção, programação, aquisição, distribuição, dispensação, garantia da qualidade dos produtos e serviços, acompanhamento e avaliação de sua utilização, na perspectiva da obtenção de resultados concretos e da melhoria da qualidade de vida da população (BRASIL, 2004).

Contudo, esse novo direcionamento da política de medicamentos no país tem enfrentado alguns desafios, pois com a evolução tecnológica e a introdução de novos procedimentos diagnósticos e terapêuticos, houve não só o aumento da expectativa de vida da população, mas também o aumento das doenças crônicas, das comorbidades e do número de medicamentos utilizados por paciente. Fato que contribuiu para o uso não racional dos medicamentos e para o aparecimento de complicações de doenças causadas pela ineficácia do tratamento, principalmente por doenças iatrogênicas e/ou por eventos adversos, levando ao aumento da morbi-mortalidade e dos custos dos tratamentos (BARR, 1955; CIPOLLE, STRAND, MORLEY, 1998; STELL et al. 1981).

2.4. Atenção farmacêutica: modelo de prática farmacêutica para o cuidado do paciente

Diante dos desafios a serem superados pela Assistência Farmacêutica como política assistencial à saúde, começou a ser introduzido um modelo de prática farmacêutica baseado em escolas internacionais. O modelo fundamentado na prática clínica do farmacêutico mostrou ser capaz de prover a melhora de resultados clínicos em relação às doenças crônicas. No Brasil, esse modelo foi denominado como ATF e fundamentado, inicialmente, nos modelos norte-americanos (CIPOLLE et al., 1998).

A ATF inseriu-se no conjunto de ações relacionado à Assistência Farmacêutica, como a prática do uso do medicamento para o cuidado do paciente. Nesta prática farmacêutica há a cooperação do farmacêutico com outros profissionais, cujo paciente é o foco das ações (HEPLER; STRAND, 1999; OPAS, 2002; BRASIL, 2007). Sendo assim, a ATF foi definida como o compêndio de atitudes, comportamentos, compromissos, inquietudes, valores éticos, funções, conhecimentos, responsabilidades e habilidades do farmacêutico no acompanhamento farmacoterapêutico com o objetivo de obter resultados terapêuticos bem definidos, promover a saúde e a qualidade de vida do paciente (CIPOLLE, STRAND, MORLEY, 1998; OPAS, 2002).

Os principais métodos de acompanhamento farmacoterapêutico que têm sido utilizados no Brasil são SOAP (Subjetivo, Objetivo, Avaliação e Plano); TOM (*Therapeutic Outcomes Monitoring*); Dáder e PWDT (*Pharmacist`s Workup of Drug Therapy*). Esse último desenvolvido em Minnesota nos EUA para ser aplicável em farmácia comunitária, cujos principais componentes são: análise de dados, plano de atenção, avaliação, intervenção,

monitoramento e registro. Para isso, é necessário realizar sete passos fundamentais: coletar e interpretar informações relevantes do paciente; identificar os problemas farmacoterapêuticos (PFT); descrever os objetivos terapêuticos desejados; selecionar e individualizar o tratamento; implementar a decisão terapêutica; e delinear o plano de monitorização para alcançar os resultados. Este método serve como diretriz para documentar as atividades clínicas não representando simplesmente um formulário para ser completado a cada entrevista farmacêutica (CIPOLLE, STRAND, MORLEY, 2006).

Alguns modelos de ATF têm surgido no Brasil na tentativa de corresponder, principalmente, ao crescente quadro de morbidades crônicas, porém ainda é uma prática incipiente. Essa prática busca ser uma alternativa aos problemas que o sistema de saúde tem enfrentado e que são agravados pelas dificuldades frente ao manejo das morbidades crônicas, as quais têm demandado muito da operacionalização do sistema de saúde e consumido muitos recursos financeiros. A ATF não tem contribuído apenas para reduzir a ocorrência das doenças iatrogênicas, mas também para promover o uso racional de medicamentos e a otimização dos recursos de saúde. É uma prática complexa que tem apresentado bons resultados no cuidado dos pacientes. Contudo, pode-se dizer que a ATF é uma TS que não está implementada no SUS. (ARAÚJO, UETA, FREITAS, 2005; LYRA, MARCELLINI, PELÁ, 2008; BORGES et al., 2011; OLIVEIRA & DE MENEZES, 2013; OLIVEIRA, 2013).

2.5. Doenças crônicas não transmissíveis: hipertensão arterial sistêmica

Em meados do século XX, o panorama mundial da saúde começou a sofrer uma importante modificação. Havia um cenário em que as doenças mais dispendiosas e preocupantes eram as doenças infecciosas, por serem grande causa de morte. No final do século XX, as doenças crônicas não transmissíveis passaram a responder pelas maiores taxas de morbi-mortalidade. Esse quadro tem se agravado em países de média e baixa renda. No Brasil, as doenças crônicas não transmissíveis são responsáveis pela maior parte das despesas com assistência ambulatorial e hospitalar (BRASIL, 2008; OMS, 2003). Adicionalmente, a assistência aos indivíduos com doenças crônicas não transmissíveis tem se tornado um grande desafio aos profissionais de saúde na atualidade, e dentre as mais importantes morbidades crônicas está a HAS (CIPULLO et al., 2010; WHO, 2011; SBC, 2010).

A HAS é uma doença silenciosa caracterizada pelo aumento sustentado da pressão arterial (PA) acima dos valores considerados normais (SBC, 2010). É uma doença considerada um problema de ordem mundial para a saúde pública (WHO, 2011), o que tem gerado investimentos e diferentes planejamentos para o cuidado do paciente hipertenso, visando à melhoria do acesso a saúde. Entretanto, mesmo assim há uma baixa taxa de pacientes com os níveis pressóricos dentro dos parâmetros satisfatórios para o controle da HAS. Adicionalmente, ela é uma doença considerada fator de risco para outras morbidades, em especial para as DCVs (SBC, 2010; ROUSH et al., 2014; WHO, 2014a).

Em 2013, a estimativa média da prevalência da HAS no Brasil foi de 21%, sendo 30% para os indivíduos com 35 anos ou mais. Dos pacientes com HAS a maioria é do sexo feminino e o nível de instrução dos indivíduos chega a 31,1% sem o ensino fundamental ou com ele incompleto. A prevalência da HAS tende a reduzir quanto maior a escolaridade, chegando a 16,7% entre aqueles com ensino fundamental completo e médio incompleto e 13,4% para indivíduos com o ensino médio completo e superior incompleto. A prevalência quanto à raça chega a ser maior entre as pessoas declaradas com cor da pele negra, 24,2%, seguida das pessoas de cor da pele branca, 22,1%, e daquelas declaradas pardas, 20,0% (Figura 1) (BRASIL, 2013a).

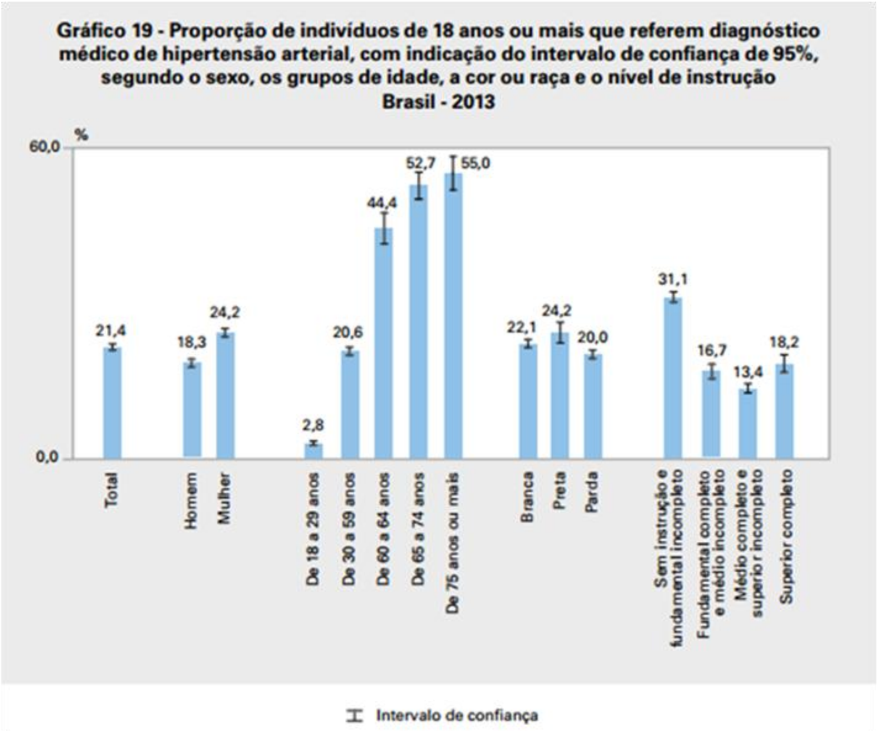

FONTE: Instituto Brasileiro de Geografia e Estatística – IBGE, coordenação de trabalho e rendimento, pesquisa nacional de saúde 2013 (BRASIL, 2013a).

Figura 1 – Caracterização dos indivíduos diagnosticados com hipertensão arterial sistêmica.

2.6. Doenças cardiovasculares associadas à hipertensão arterial sistêmica

A HAS é considerada um dos principais fatores de risco associados às DCVs. É uma comorbidade de grande prevalência ao se analisar a história clínica pregressa dos indivíduos que apresentaram DCVs (LUNA, 2009). Por definição da *WHO* (2011), as DCVs formam um grupo de doenças do coração e dos vasos sanguíneos que incluem como principais manifestações: DIC, doença vascular encefálica representada na maioria das vezes por AVE, doença arterial periférica (DAP), doença reumática do coração, doença cardíaca congênita, trombose venosa profunda e embolia pulmonar. De origem multifatorial, as DCVs possuem fatores de risco não modificáveis como o sexo, idade e histórico familiar, e modificáveis, ou controláveis, como a dislipidemia, diabetes, tabagismo, sedentarismo, alcoolismo, obesidade e HAS. Dentre estes, os fatores modificáveis são responsáveis por 80% das DCVs, sendo a HAS o de maior relevância (MAGNANINI et al., 2009; MAIA et al., 2007; NASCIMENTO, GOMES, SARDINHA, 2011; WHO, 2013).

A elevação da pressão sanguínea associa-se ao aumento do risco de DCVs (LENFANT, 2003). Os valores para a pressão sistólica menor que 140 mmHg e para a diastólica menor que 90 mmHg são preconizados pela VI diretriz da SBC (2010) como níveis satisfatórios de PA para indivíduos sem demais comorbidades e não idosos. Quando os valores de PA estão em níveis insatisfatórios de forma sustentada, a HAS torna-se a principal causa de infarto agudo do miocárdio, paradas cardíacas, derrames cerebrais, isquemias cerebrais, doenças ateroscleróticas, insuficiência cardíaca e doenças renais (SBC, 2010). Esse fato torna-se preocupante devido a grande parte dos pacientes hipertensos possuírem a pressão arterial em níveis insatisfatórios (SBC, 2010, PONTES-NETO et al., 2008; ROSÁRIO et al., 2009). Adicionalmente, para os indivíduos na faixa etária de 40-70 anos, cada incremento de 20 mmHg na pressão sistólica ou 10 mmHg na pressão diastólica pode duplicar o risco de DCVs e aumentar as chances de complicações da HAS (LENFANT, 2003; ROSAMOND et al., 2008). Esse risco agrava-se para o sexo masculino, mas com o aumento da idade o risco torna-se próximo para ambos os sexos (Figura 2).

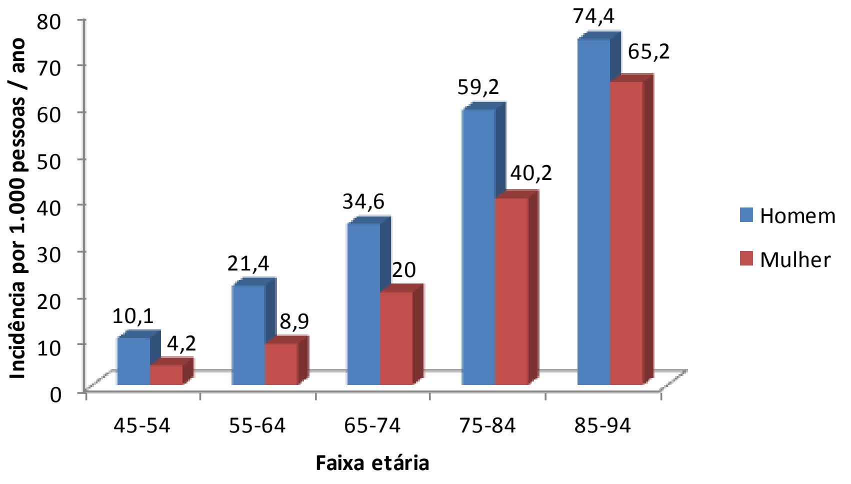

FONTE: Figura adaptada de Rosamond et al., 2008.

Legenda da Figura 2: A taxa de incidência das DCVs incorpora acidente vascular encefálico, doença isquêmica do coração, Insuficiência cardíaca.

Figura 2 – Incidência de doenças cardiovasculares de acordo com a faixa etária e o sexo.

2.7. Morbidades associadas à hipertensão arterial sistêmica como complicações da doença

Dentre as principais complicações de saúde associadas aos pacientes hipertensos a *WHO* (2011) destaca como as mais prevalentes DCVs: AVE, DIC, DAP e insuficiência cardíaca (IC). Há também outra morbidade que não é uma DCV, mas também está fortemente associada à HAS, a doença renal crônica (DRC) (ALLEN et al., 2009; WHO, 2014a, WHO, 2014b).

No mundo, uma das causas mais comuns de incapacidade em adultos é o AVE (ALLEN et al., 2009). Estima-se que cerca de 15 milhões de pessoas por ano sofram esse tipo de comorbidade (BARRETO, 2011). Após o primeiro episódio, as sequelas de hemiplegia estão presentes em 70 a 80% dos casos e parte dessa população não retorna às suas atividades funcionais (ALLEN et al., 2009). O AVE pode ser definido como um “desenvolvimento rápido de sinais clínicos de distúrbios focais (ou globais) da função cerebral com sintomas que perduram por um período superior a 24 horas ou conduzem a morte, sem outra causa aparente que a de origem vascular” (OMS, 2004). A relação da HAS com o AVE é bem elucidada há anos, a elevação da pressão arterial é considerada o principal fator de risco modificável para o AVE (SACCO et al., 1999). De acordo com Zhang et al. (2008) a presença

de níveis pressóricos elevados é comum na apresentação inicial do AVE e implica em pior prognóstico funcional.

As DICs também representam um grave problema de saúde pública no mundo (LAURENTI et al., 2000), principalmente em países desenvolvidos e em desenvolvimento (REDDY e YUSUF, 1998; WHO, 2011). Somente em 2011, no Brasil, 39,1% dos óbitos por doença do aparelho circulatório foi em decorrência das DICs, acometendo principalmente a população adulta em plena fase produtiva, situada na faixa etária de 20 a 59 anos (BRASIL, 2010). As DICs envolvem uma série de comorbidades, sendo as de maior prevalência o infarto e a angina (PIEGAS et al., 2009), respectivamente em homens e mulheres (LAURENTI et al., 2000). De acordo com Antman et al. (2013) a DIC é uma condição em que há um fornecimento insuficiente de sangue e oxigênio a uma porção do miocárdio, o que na maioria das vezes é em decorrência do desequilíbrio entre a oferta e a procura de oxigênio. A causa mais comum de isquemia é a doença aterosclerótica, no entanto, a HAS é um dos fatores potencialmente modificáveis que, de acordo com Moraes e Freitas (2012) pode influenciar no desfecho da DIC.

A DAP pode ser definida como um conjunto de síndromes crônicas ou agudas que alteram o fluxo de sangue e de oxigênio para os membros, por vezes em decorrência da doença aterosclerótica (HERNANDO e CONEJERO, 2007). Presume-se que 20% da população com mais de 55 anos é portadora de DAP (HANKEY et al., 2006). A sua principal complicação clínica é a doença arterial obstrutiva periférica (DAOP), processo patológico gradual, sintomático ou assintomático, que pode culminar com a obstrução de artérias dos membros inferiores (GARCIA, 2006). Representa a principal causa de amputação de extremidades e com grave risco de morbimortalidade cardiovascular (NORGREN et al., 2007; SBACV, 2014). A claudicação intermitente é o mais frequente dos sintomas da DAOP marcada por dor ou desconforto durante o exercício, que desaparece após o repouso (SBACV, 2014). De acordo com Piccinato et al. (2001), a hipertensão é o fator mais potente no desenvolvimento da doença aterosclerótica, por isso, a sua forte relação com a DAP (GARCIA, 2006; HERNANDO e CONEJERO, 2007; SBACV, 2014).

A IC é uma das principais causas de hospitalizações entre as doenças cardiovasculares (BARRETO, 2001), considerada a via final da maioria dos problemas que acometem o coração. Doença de alta prevalência, alto custo, frequentemente incapacitante e de elevada taxa de mortalidade, uma das alterações de saúde em progressão no mundo moderno

(ALBANESI-FILHO, 2005; BOCCHI et al., 2009). De acordo com a III diretriz Brasileira de Insuficiência Cardíaca Crônica (2009) essa síndrome tem caráter sistêmico e é definida como disfunção cardíaca que compromete o suprimento sanguíneo tecidual ou é capaz de realizá-lo apenas sob elevada pressão de enchimento (BOCCHI et al., 2009). A HAS está frequentemente associada a IC tanto como causa, quanto como fator de agravamento da doença (BARRETO, 2001). No estudo em Framingham, 90% dos pacientes com IC possuíam HAS no momento do diagnóstico (LEVY et al., 1996; LENFANT, 1999), sendo ela o determinante substancial para o aumento do risco de desenvolver a IC (STROKES et al., 1989). No entanto, de acordo com Mesquita et al. (2004) a pressão arterial quando controlada contribui na prevenção da IC e na redução de sua incidência. Bocchi et al. (2005) reforça ainda a necessidade do controle pressórico rigoroso em pacientes com IC, a fim de evitar as complicações da doença.

A HAS pode comprometer a homeostase dos rins, órgão alvo de lesão, um dos principais fatores de risco para o desenvolvimento da DRC (USRDS 2009). No Brasil, a incidência e a prevalência da DRC aumentam a cada ano e, geralmente, está associada a um prognóstico ruim, evolução lenta e assintomática (SESSO et al., 2008; MS, 2014). De acordo com as Diretrizes Clínicas para o Cuidado de pacientes com DRC, essa comorbidade é definida pela presença de lesão renal, associada ou não a diminuição da filtração glomerular <60mL/min/1,73 m^2, por um período de três meses, onde ocorre perda progressiva e irreversível da função dos rins (ROMÃO-JÚNIOR, 2004; MS, 2014). Em sua fase mais avançada os rins não conseguem manter a normalidade do meio interno do paciente (ROMÃO-JÚNIOR, 2004), o que é caracterizado como DRC terminal, havendo a necessidade da terapia renal substitutiva como a hemodiálise, a diálise peritoneal e o transplante renal, que possui um alto custo para os sistemas de saúde (BRASIL, 2014). De acordo com Klahr et al. (1994) o controle pressórico adequado pode retardar a incidência e progressão da DRC.

2.8. Medida do risco cardiovascular pela escala *ASCVD*

Juntamente com a HAS outros fatores estão associados ao risco cardiovascular como: hiperlipidemia, obesidade, tabagismo, diabetes, alcoolismo, etnia, histórico familiar e hábito de vida e alimentar (SBC, 2010). Para o cuidado do paciente hipertenso é realizada a estratificação do risco cardiovascular que muitas vezes é feito por escalas de risco padronizadas (LOTUFO, 2008; STONE et al., 2013).

A escala para o risco de doença aterosclerótica cardiovascular *(ASCVD)* mensura o risco da incidência de DCV. Seu escore é gerado em percentual, o que é definido como o percentual de risco que o paciente tem de apresentar uma doença cardiovascular em dez anos. Essa escala de risco foi publicada na diretriz norte-americana *(American College of Cardiology/American Heart Association - ACC/AHA)* em 2013 e difere de algumas escalas utilizadas por apresentar maior sensibilidade à incidência de DCVs e por ser um método de cálculo que estima o risco em 100% de chance para as doenças cardiovasculares em geral, não somente para doenças coronarianas. Ela permite calcular os escores quanto aos fatores de risco totais e somente os fatores modificáveis. Para isso ele considera as variáveis: idade, sexo, os valores de pressão arterial, colesterol total (CT) e a sua fração *high-density lipoprotein* (HDL), além das comorbidades como HAS, tabagismo e diabetes (DAWBER, MOORE-Jr, MANN, 1957; LOTUFO, 2008; STONE et al., 2013; MUNTNER et al., 2014).

2.9. Análises de decisão associada a modelagens para estimativas das complicações de doenças crônicas

A análise de decisão envolve a sistematização de processos racionais para selecionar ou fundamentar uma alternativa dentro de várias possibilidades de escolha. Ela consiste na aplicação de um método analítico que permite a comparação sistemática das diferentes possibilidades. Alguns autores definem certas etapas para a sua construção que em suma seriam: identificar a decisão específica; especificar alternativas; traçar a estrutura de análise; especificar os possíveis custos, desfechos e probabilidades; e realizar os cálculos. A estrutura de uma análise de decisão pode ser desenhada de diferentes formas, uma delas que é muito empregada na área da saúde, é a árvore de decisão devido a sua melhor adequação à aplicação de modelagens matemáticas (CHEN et al., 2013; RASCATI, 2010).

A modelagem de Markov é uma ferramenta matemática atribuída às análises de decisões farmacoeconômicas com a finalidade de projetar estados de saúde e complicações do tratamento. Esse modelo matemático é muito utilizado para as doenças crônicas, uma vez que, constituem desfechos mais complexos por longo período de tempo. Com a aplicação dessa modelagem é possível atribuir as transições de estados de saúde ao longo dos anos, determinados por ciclos, o que permite realizar uma projeção de acordo com a distribuição de probabilidades em um horizonte de tempo e, no final, obter da coorte de pacientes os indivíduos alocados em diferentes estados de saúde. A cada estado de saúde pode ser atribuído um custo e então realizar análises farmacoeconômicas para diferentes medicamentos

ou intervenções (RASCATI, 2010; STEVANOVIC, POSTMA, PECHLIVANOGLOU, 2012).

2.10. Avaliação econômica e farmacoeconomia

Quando a avaliação econômica está associada a produtos e serviços assistenciais a saúde, a farmacoeconomia é o método eficiente de medida e avaliação de custos (RASCATI, 2010, MOTA, 2003). Existem diferentes estudos farmacoeconômicos que vão diferir na sua metodologia quanto aos diferentes objetivos das análises, sendo que cada um deve ser escolhido de acordo com as características a serem avaliadas. Ao considerar para a análise programas ou modelos de intervenções, a farmacoeconomia pode ser empregada como método para mensurar o benefício e verificar se a intervenção compensa o custo atribuído a ela, o que é denominado estudo de custo-benefício, ou para medir um resultado clínico em termos de custos, denominado estudo de custo-efetividade, ou ainda para medir a qualidade em saúde em relação ao custo, estudo de custo-utilidade (DRUMOND et al., 2005; RASCATI, 2010).

As análises farmacoeconômicas de custo-efetividade são utilizadas, por exemplo, para a medição dos custos em moeda e dos desfechos em unidades de saúde, tais como: curas, sobrevida, redução da pressão sanguínea. Os desfechos não precisam ser convertidos em valores monetários (RASCATI, 2010). Uma variante deste modelo, também considerada um tipo de estudo é o estudo de custo-utilidade, o qual mensura o custo de um determinado parâmetro para a qualidade de vida ou amenização da doença (NITA et al., 2009; RASCATI, 2010).

Nas análises de custo-benefício devem ser atribuídos valores monetários aos desfechos, isso torna possível determinar se os benefícios de um programa ou de uma intervenção excedem os custos de sua implementação, o que permite a comparação de diversos programas diferentes através dos cálculos do benefício líquido e da razão benefício / custo ou custo / benefício. Este modelo de estudo permite maior abrangência para aplicações de métodos econômicos e de econometria (NITA et al., 2009; RASCATI, 2010; VALE, STEFFENS, DONALDSON, 2004; MINISTÉRIO DA SAÚDE, 2009).

O valor presente (VP) é um indicador econômico atribuível às análises de custo-benefício. Os valores prometidos no futuro representam um menor valor no presente, dessa forma há a necessidade de corrigi-los. Esses valores podem ser ajustados com as taxas

econômicas de mercado no país, como a inflação e as taxas de juros e índice nacional de preço ao consumidor. O método do *discounting*, por exemplo, é empregado na realização deste ajuste para trazer as economias prometidas no futuro para o VP, e quando o VP remete a diferença entre os custos e benefícios, torna-se o valor presente líquido (VPL) (NETO & LIMA, 2009; RASCATI, 2010).

Todos esses tipos de estudos farmacoeconômicos necessitam que as suas informações sejam validades para a conclusão das análises, para isso, é preciso realizar a análise de sensibilidade. A análise de sensibilidade remete em estimativas a variação ou o limiar dos custos, refletindo possibilidades realísticas desses valores ao correlaciona-los aos limites pré-estabelecidos (NITA et al., 2009; RASCATI, 2010).

2.11. Hipótese do estudo

Diante desta contextualização, foi formulada a pergunta deste estudo "como realizar uma análise econômica capaz de fundamentar a tomada de decisão para os gestores de saúde quanto a implementação ou não da ATF no SUS?". Sendo assim, a hipótese deste estudo que pretende responder essa pergunta é a seguinte: com base em um estudo farmacoeconômico em longo prazo, utilizando conceitos econômicos, métodos de econometria e de epidemiologia, é possível construir um modelo de análise capaz de subsidiar a tomada de decisão quanto ao investimento na ATF para o tratamento de pacientes hipertensos, podendo mostrar não ser necessário o desinvestimento em outros serviços para implantação da ATF no SUS por ser uma prática capaz de trazer um retorno monetário maior que o investimento quando considerado as complicações da HAS no horizonte do tempo.

3. OBJETIVOS

3.1. Objetivo geral

Realizar uma análise farmacoeconômica com os custos diretos e projeções futuras de custos para avaliar o impacto da Atenção Farmacêutica no cuidado de pacientes com hipertensão arterial sistêmica para o Sistema Único de Saúde e com isso, elaborar uma ferramenta econômica que considere as variáveis de custos e os desfechos em saúde capaz de auxiliar os gestores de saúde na tomada de decisão para a implementação desta prática profissional nos serviços de saúde e, assim, reduzir a morbi-mortalidade ocasionada pela hipertensão arterial sistêmica.

3.2. Objetivos específicos

- Analisar o impacto da Atenção Farmacêutica quanto aos indicadores clínicos e assistenciais após a alta dos pacientes;
- Obter os custos diretos relativos à assistência à saúde do paciente hipertenso no Brasil;
- Descrever e analisar os custos diretos comparando o modelo convencional de assistência aos pacientes hipertensos e o modelo incluindo a Atenção Farmacêutica;
- Realizar a análise de custo-efetividade para os resultados dos custos diretos;
- Estimar a ocorrência de complicações da hipertensão arterial no horizonte de dez anos;
- Obter os desfechos de anos de vida ganhos e anos de vida ajustados pela qualidade para realizar a análise de custo-efetividade e custo-utilidade;
- Obter o valor presente líquido relativo ao benefício trazido pela Atenção Farmacêutica para realizar a análise de custo-benefício na projeção de dez anos com os custos diretos e indiretos;
- Analisar o impacto epidemiológico obtendo o número necessário para tratar, *Odds Ratio* e redução relativa de risco;
- Realizar a análise de sensibilidade para o retorno de investimento da Atenção Farmacêutica.

4. CASUÍSTICA E MÉTODOS

4.1. Desenho do estudo

Este é um estudo farmacoeconômico de modelagem com análises de custo-efetividade, custo-benefício e custo-utilidade. De março a novembro de 2014 foram coletados os dados para este estudo, sendo eles divididos em clínicos, assistenciais e econômicos. Os dados clínicos e assistenciais referem-se ao acompanhamento de 104 pacientes hipertensos durante os anos de 2006 a 2012 pelo Centro de Pesquisa em Assistência Farmacêutica e Farmácia Clinica (CPAFF) da Faculdade de Ciências Farmacêuticas de Ribeirão Preto, Universidade de São Paulo (FCFRP-USP). No ano de 2009, foi realizado o programa de ATF destinado ao cuidado dos pacientes com HAS, os quais foram acompanhados por doze meses. Dessa forma, os dados clínicos dos três anos anteriores ao programa de ATF (2006-2008) representaram para este estudo os anos sem a ATF, enquanto que 2009 representou o ano com ATF e os três anos posteriores (2010-2012) representaram os anos pós ATF. Os dados clínicos foram coletados por meio do prontuário do paciente e do prontuário farmacêutico, e os assistenciais por meio do sistema informatizado Hygiaweb®.

4.2. Programa de Atenção Farmacêutica realizado para o cuidado da hipertensão

Foram incluídos no programa de ATF os indivíduos diagnosticados com HAS, residentes em Ribeirão Preto-SP, com idade superior a 20 anos, em acompanhamento médico para a morbidade, usuários do SUS e em administração de medicamento anti-hipertensivo (AH). Foram excluídos os pacientes que não puderam dar continuidade ao planejamento das consultas de ATF, gestantes, lactantes, e aqueles que possuíam comprometimento cognitivo diagnosticado.

O programa de ATF foi colocado em prática em duas unidades básicas do SUS no município de Ribeirão Preto-SP. Foram programadas doze consultas farmacêuticas baseadas nas estratégias de acompanhamento farmacoterapêutico do modelo norte-americano PWDT (CIPOLLE, STRAND, MORLEY, 1998). Um farmacêutico foi responsável por conduzir o programa e realizar as consultas, que ocorreram mensalmente para cada paciente. Na primeira consulta foi realizado o levantamento da história clínica do paciente, estilo de vida, hábitos alimentares, dados sociodemográficos (sexo, idade, cor da pele, escolaridade, atividade ocupacional, renda) e as medidas relativas ao perfil do risco cardiovascular (obesidade, dislipidemia, antecedente familiar, tabagismo, diabetes e circunferência abdominal).

As consultas posteriores seguiram as atividades pertinentes ao acompanhamento farmacoterapêutico, contemplando aferições da pressão arterial e medidas do risco cardiovascular; análise dos medicamentos e dos resultados dos exames; educação em saúde, com as orientações sobre as condutas do paciente quanto ao hábito de vida, adesão ao tratamento; e se necessário foram realizadas intervenções na farmacoterapia. Ao analisar os medicamentos em uso pelo paciente, o farmacêutico verificava a presença de possíveis PFT. Detectando algum PFT, o farmacêutico preenchia um formulário contendo a descriminação do medicamento em questão (nome, apresentação, dose diária, forma farmacêutica e horário da administração), classificava o PFT de acordo com o PWDT, descrevia a intervenção a ser realizada, elencava as metas quanto aos resultados clínicos a serem alcançados com a intervenção, estabelecia a forma de monitoramento desses resultados, designava o prazo para a reavaliação do resultado clínico e, posteriormente, preenchia o campo relativo à adesão e à intervenção ao prescritor. Em adição a isso, o paciente era informado e orientado sobre as novas condutas em sua farmacoterapia, caso houvesse a adesão do prescritor.

A educação em saúde foi fundamentalmente trabalhada pelo farmacêutico neste programa de ATF. Eram realizadas palestras informativas, com materiais educativos em saúde e orientações nas consultas. Não obstante, a adesão à terapia medicamentosa também foi fortemente trabalhada com os pacientes. Ambas as condutas tiveram o objetivo de empoderar o paciente frente ao seu tratamento conscientizando-o sobre a sua doença, as gravidades, as complicações e os cuidados necessários. Essas estratégias tiveram seus resultados mensurados pelos instrumentos SF-36 (*The medical outcomes study 36-item short-form health survey*), e Morisky-Green com quatro perguntas. Sendo o primeiro para avaliar a qualidade de vida e o segundo para avaliar a adesão à farmacoterapia pelo auto relato do paciente quanto à utilização dos medicamentos (CICONELLI et al., 1999; MORISKY, GREEN, LEVINE, 1986).

4.3. Indicadores clínicos e assistenciais do estudo

Os indicadores avaliados no acompanhamento dos pacientes são relativos aos dados clínicos e assistenciais. Os indicadores clínicos referem-se às aferições da PAS e PAD, os resultados de triglicerídeos (TG), CT e as suas frações de Lipoproteína de Baixa Densidade (LDL) e de HDL. Os assistenciais correspondem ao número anual de consultas e o consumo anual de medicamentos AH. As consultas foram classificadas em atendimento básico (consultas com clínico geral e médico da saúde da família), pronto atendimento (consultas por

crises hipertensivas em urgência e emergência) e atendimento especializado (consultas na especialidade de cardiologia), e os medicamentos AH foram considerados aqueles fornecidos pelo SUS, pertencentes à Relação Municipal de Medicamentos Essenciais (REMUME).

4.4. Estatística e planejamento amostral

Foi realizado o planejamento amostral com base no cálculo estatístico para estudos experimentais, utilizando a estimativa da média populacional para a variável de interesse: pressão arterial. Para o cálculo foi utilizado $n = (Z_{\alpha/2} \times \Delta dp / \varepsilon)^2$, onde: $Z_{\alpha/2}$ é o percentil da normal padrão para um nível de significância de 5%; Δdp é a soma dos desvios padrão antes e após a intervenção; ε é a precisão, diferença clinicamente significativa após a intervenção. A partir de dados da literatura foi considerado a soma dos desvios padrão antes e após a intervenção igual a 30 mmHg (LYRA, MARCELLINI, PELÁ, 2008; BORGES, 2008) e clinicamente satisfatória a redução da pressão arterial sistólica entre 5-10 mmHg (AGUWA, UKWE, EKWUNIFE, 2008; LYRA, MARCELLINI, PELÁ, 2008). Logo, para o valor de ε entre 5-10 mmHg foi calculado o n entre 33 e 138.

Para a análise dos indicadores clínicos os dados foram categorizados em satisfatório e insatisfatório quanto aos valores de referência de cada indicador. Para isso foram utilizados os valores de referência para pressão arterial de acordo com a diretriz norte-americana referente ao *VIII Joint National Committee de 2014 - VIII JNC.* O qual estabelece como valores satisfatórios a pressão arterial <150x90 mmHg para indivíduos hipertensos maiores de 60 anos, sem diabetes e sem doença renal crônica, e pressão arterial <140x90 mmHg para indivíduos maiores de 18 anos com hipertensão, associada ou não ao diabetes e à doença renal crônica. Para os níveis de referência do colesterol foi utilizado a diretriz de dislipidemia da *American Association of Clinical Endocrinologists de 2012 (AACE).* De acordo com o estadiamento clínico do risco coronariano calculado pelo escore de risco de Framingham, os pacientes foram classificados em alto, intermediário e baixo risco. Adicionalmente, para cada estadio de risco foi atribuído um valor de referência como satisfatório, conforme estabelecido pela diretriz. Sendo para o CT, <200 mg/dL para alto risco e entre 200-239 mg/dL para os dois últimos estádios de risco; para o LDL, <100 mg/dL, <130 mg/dL, <160 mg/dL respectivamente para os três estádios de risco; para o HDL, >40 mg/dL para homens e >50 mg/dL para mulheres; para o TG <150 mg/dL para alto risco e entre 150-199 mg/dL para os dois últimos estádios de risco.

Quanto à análise das variáveis assistenciais, ressalta-se que foi obtida pela base de dados *Micromedex®* a dose máxima de cada medicamento AH utilizado pelos pacientes deste estudo. A dose máxima foi mimetizada como a potência farmacoterapêutica de cada medicamento para o tratamento da HAS. Com isso foi possível, encontrar o mínimo múltiplo comum entre as doses máximas de todos os medicamentos AH para dividir pela dose máxima de cada medicamento, o que permitiu encontrar um valor fixo de cada medicamento (valor de ajuste). Esse valor foi multiplicado pelo consumo em miligrama (mg) do respectivo medicamento por cada paciente, o que tornou os diferentes medicamentos comparáveis para a análise neste trabalho (Quadro 1).

Quadro 1 – Exemplo do modelo de ajuste de doses desenvolvido neste estudo para a comparação e análise terapêutica de medicamentos

36 = MMC encontrado considerando todos medicamentos anti-hipertensivos utilizados.		
Medicamento:	**Enalapril**	**Anlodipino**
Dose maxima:	40 mg	10 mg
Valor de ajuste:	36 / 40 = **0,9**	36 / 10 = **3,6**
Consumo do paciente:	20 mg por dia	5 mg por dia
Ajuste:	20 mg x 0,9 = **18 mg**	5mg x 3,6 = **18 mg**

Nota Quadro 1: Este método de ajuste de doses para a comparação e análise da utilização de medicamentos está em processo de registro de propriedade intelectual. Neste processo também é incluído o ajuste realizado pelo máximo divisor comum. Assim, esse método não pode ser usado com propostas comerciais. Em adição, ele é autorizado para publicações acadêmicas desde que os autores deste método sejam citados, "*therapeutic doses for comparison model* (TDC model) por Cazarim & Pereira" (pela Agência USP de Inovação).

Para a análise estatística inferencial foi utilizado o *software Statistical Analysis System* (SAS) versão 9.2. Foi considerado o nível de significância de 5% para os testes de hipótese. É importante ressaltar que os dados foram divididos em três períodos para análise: período pré ATF (2006 - 2008), período ATF (2009) e período pós ATF (2010 – 2012).

A estatística inferencial fundamentou-se em medidas repetidas, sendo referentes aos mesmos indivíduos da amostra para análise em momentos temporais diferentes. Sendo assim, para as variáveis clínicas foi realizado o teste *Q de Cochran*, utilizado para comparar variáveis do tipo categóricas. A finalidade foi testar a hipótese que a ATF está associada ao controle desses indicadores. Para isso, foi considerada a distribuição chi-quadrado para 2 graus de liberdade, na qual teve o valor crítico para análise dos resultados de 5,99. Quanto as variáveis assistenciais, o consumo de medicamentos AH em mg, variável do tipo quantitativa contínua, a qual apresentou uma distribuição paramétrica para os dados, foi analisada por *ANOVA one*

way para medidas repetidas com pós-teste *Bonferroni* para a comparação entre os grupos de dados pré ATF, ATF e pós ATF. A finalidade foi testar a hipótese que a modificação na média do consumo de medicamentos AH está associada à ATF. Em relação ao número de consultas, por corresponder a variável do tipo quantitativa discreta com uma distribuição não paramétrica, foi realizada a análise de variância de *Friedman* com pós-teste de *Dunns* para a comparação entre os grupos de dados pré ATF, ATF e pós ATF. O objetivo desta análise foi testar a hipótese se há modificação no número de consultas associada à ATF. Na análise do risco cardiovascular também foi realizado *ANOVA one way* para medidas repetidas com pós-teste *Bonferroni* para testar a hipótese que a redução na média do risco cardiovascular em dez anos está associada à ATF.

4.5. Análise farmacoeconômica

As etapas do estudo farmacoeconômico foram definidas de acordo com as análises a serem realizadas para a elaboração da ferramenta farmacoeconômica (Figura 3).

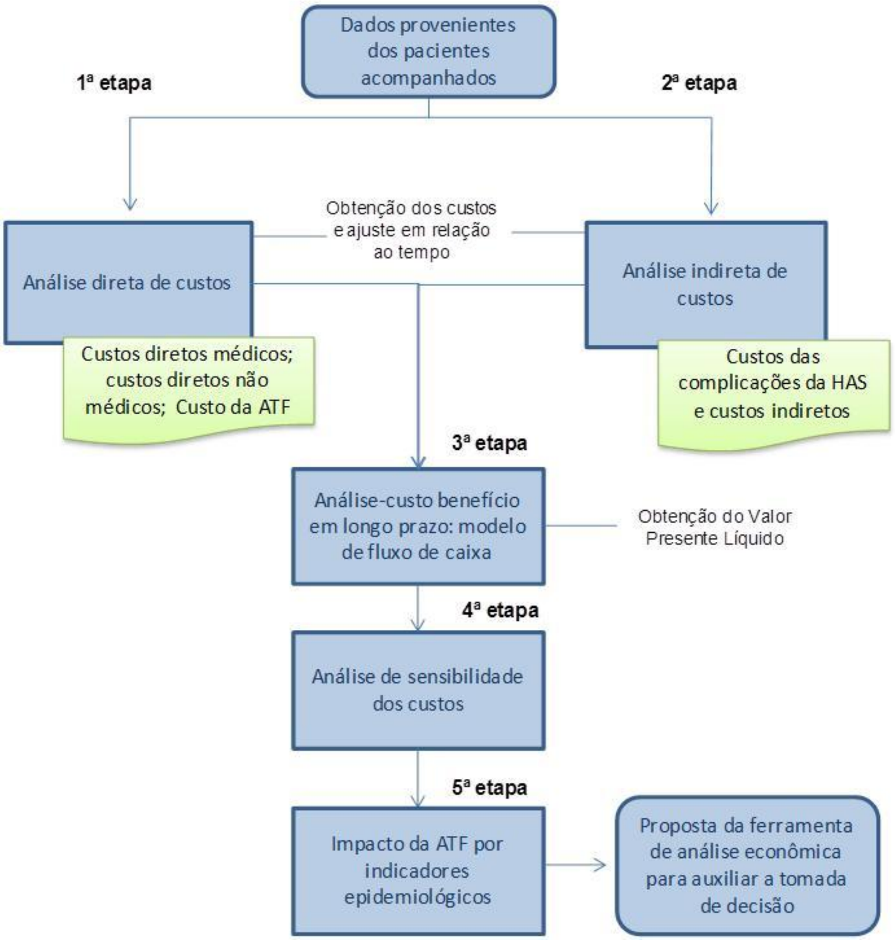

Legenda Figura 3: ATF = Atenção Farmacêutica; HAS = Hipertensão arterial sistêmica.

Figura 3 – Fluxograma da elaboração da ferramenta econômica.

4.5.1. Obtenção e categorização dos custos

Os custos considerados neste estudo foram os diretos não médicos, os diretos médicos, indiretos e o custo com a Atenção Farmacêutica. Ressalta-se que o custo das complicações da HAS faz parte dos custos direto médico, porém foram considerados em um subgrupo por não entrarem na análise direta dos custos, mas sim projetados na análise indireta. Os custos coletados foram referentes ao ano de 2013, por ser o último ano de dados completos da Secretaria Municipal de Saúde de Ribeirão Preto (SMS-RP) no período de coleta de dados deste estudo. Dessa forma, houve necessidade de realizar o ajuste do custo para o ano de 2015, o que ocorreu, também, para os outros custos para a análise indireta coletados na literatura. Para isso, foi considerado o Índice Nacional de Preços ao Consumidor (INPC), disponibilizado nos indicadores econômicos consolidados do Banco Central do Brasil (BRASIL, 2013b; BRASIL, 2014).

4.5.1.1. Custos diretos não médicos

Os custos não médicos são aqueles que estão relacionados aos cuidados de saúde e não estão diretamente relacionados à saúde do paciente. Dentre eles foram considerados o absenteísmo, compreendido pela falta no serviço devido às consultas, e o transporte em condução pública para ida e volta da consulta. Esses custos são relevantes para a análise, pois a ATF é capaz de influenciar no número de consultas quanto ao cuidado da HAS (PENAFORTE, 2011). Sendo assim, o absenteísmo foi calculado de acordo com rendimento bruto médio anual entre os pacientes que participaram deste estudo e que trabalhavam. Para o cálculo foi considerado o que o paciente deixaria de produzir para a economia do país, com base no valor da sua produção (o salário que recebe), em decorrência do tratamento para a HAS (CHIAVENATO, 2004). Para isso, foi utilizada como base para o cálculo 8 horas de trabalho por dia, um mês de férias, 20 dias de trabalho no mês (retirados os finais de semana e os feriados). O rendimento bruto médio anual, considerando 13º salário, foi dividido pelo total de horas trabalhadas no ano, obtendo o valor da hora trabalhada. Esse valor foi multiplicado por meio período de dia trabalhado, quatro horas (considerado por faltar ao serviço para ir à consulta).

Para os custos relacionados ao transporte foi considerado que o paciente utilizou a condução pública para ida e volta das consultas. Para o cálculo foi utilizado o valor da tarifa única cobrada no município em 2013. Esse custo foi atribuído para aqueles pacientes que não

contribuem com impostos para transporte publico ou tem o direito gratuito, ou seja, os trabalhadores autônomos, idosos e não trabalhadores (BRASIL, 2015a).

4.5.1.2. Custo diretos médicos

Foram elencados para esta categoria de custo, os custos com exames laboratoriais, consultas e medicamentos AH. Quanto aos exames, foram considerados aqueles exames utilizados para o diagnóstico da dislipidemia, preconizados ao acompanhamento da HAS pela IV diretriz da SBC (2010), por ser uma morbidade associada a fator de risco para DCV. Dessa forma, foram incluídos os exames de TG, CT e suas frações LDL e HDL (AACE, 2012). O custo foi obtido pela *tabela unificada do sistema de gerenciamento da tabela de procedimentos, medicamentos e OPM do SUS* (SIGTAP) (BRASIL, 2015b). O cálculo foi realizado para cada paciente, multiplicando o número total de exame anual pelo custo unitário do exame.

As consultas foram classificadas em consultas da ATF (consultas com o farmacêutico, utilizadas para o cálculo de custo da ATF), atendimento básico, pronto atendimento e atendimento especializado. Foram obtidos os dados discriminados dos custos municipais de cada unidade de saúde do município pela Divisão de Finanças e de Custo Operacional da SMS-RP, e o número de atendimentos discriminados por setor/especialidade de cada unidade de saúde do município pelo departamento de Informática, Estatística, Controle e Auditoria da SMS-RP. As despesas totais que foram incluídas neste estudo com as unidades de saúde remetem aos custos categorizados em escritório, limpeza, enfermagem, diversos, permanente, salário efetivos, salário contratados (IPMF, sassom, INSS + seguro, FGTS, vale transporte, vale alimentação), água, energia, telefone, serviços, prestadores, aluguéis, viagem, pequenas despesas eventuais taxadas como pronto pagamento, combustíveis e gastos com a Fundação de Apoio ao Ensino, Pesquisa e Assistência do Hospital das Clínicas da Faculdade de Medicina de Ribeirão Preto da Universidade de São Paulo - FAEPA. Sendo excluídos os custos de odontologia, radiografia, medicamentos, este último por ser uma variável de custo analisada separadamente.

O atendimento básico foi considerado em nosso estudo como um atendimento generalista à condição de saúde do paciente, no qual há poucas discrepâncias entre a média dos custos de um paciente hipertenso com a média em geral dos pacientes com outras morbidades. Dessa forma, foram utilizadas as despesas totais com as Unidades Básicas de

Saúde (UBS) consideradas neste estudo e Estratégia Saúde da Família (ESF) e o total de atendimentos nas unidades de saúde. Para o cálculo foi realizada a divisão entre o custo anual e o número anual de atendimentos de cada unidade, obtendo a média entre elas como o custo de um atendimento neste segmento.

Quanto às consultas de pronto atendimento e atendimento especializado foi considerado que não são atendimentos generalistas em termos de custos para as diferentes morbidades. O paciente hipertenso tem um custo diferenciado em cada um desses segmentos e esses custos consideram a logística do atendimento padronizado pela SMS-RP no SUS (BRASIL, 2015c). Sendo assim, foi calculado o custo basal das unidades de pronto atendimento e o custo basal das Unidades Básicas Distritais de Saúde (UBDS) para os atendimentos ambulatoriais. Esse custo remete a despesa que se tem em comum a qualquer tipo de paciente que entra na unidade para ser atendido. Para isso, foram consideradas as categorias de custos escritório, limpeza, diversos, permanente, energia, telefone, serviço, prestadores, aluguéis, viagem, pronto pagamento e combustíveis. Para o cálculo foi realizada a divisão entre o custo anual e o número anual de atendimentos de cada unidade, obtendo a média entre as UBDS como o custo basal de um atendimento especializado e a média entre as unidades de pronto atendimento como o custo basal de um atendimento de urgência ou emergência.

Para calcular o custo da consulta do atendimento especializado foi considerado o custo do exame eletrocardiograma, exame preconizado pela SBC (2010) como avaliação de rotina, do atendimento de enfermagem, do atendimento da especialidade de cardiologia (BRASIL, 2015c), do acompanhamento médico, sendo essas quatro categorias obtidas da tabela unificada SUS, competência de 2013 (BRASIL, 2015b). Para compor o cálculo, esses custos foram adicionados ao custo basal da unidade de atendimento. O custo com monitoramento foi elencado de acordo com a VI diretriz da SBC (2010). Assim, foram considerados os exames de rotina (urina tipo I, potássio plasmático, creatinina sérica, ácido úrico e glicemia de jejum), e a cada dois anos os exames de ultrassonografia doppler colorido e angiografia de arco aórtico, pois estes exames são preconizados para a avaliação complementar de lesões clínicas e subclínicas em órgão alvo. Ressalta-se que para o cálculo de custo do atendimento foi considerado um ECG a cada consulta e os exames de acompanhamento médico a cada seis consultas, considerando a média de três consultas anuais com o médico cardiologista (BRASIL, 2015c; SBC, 2010).

Para o cálculo do custo da consulta de pronto atendimento foi considerado o custo do atendimento médico, do atendimento de enfermagem, dos exames preconizados pela VI diretriz da SBC (2010) como o ECG, radiografia do tórax e creatinofosfoquinase, obtidos pela tabela unificada SUS (BRASIL, 2015b). Esses custos foram adicionados ao custo basal da unidade de atendimento e ao custo do medicamento de urgência (BRASIL, 2015c, SBC, 2010). O custo do medicamento foi calculado pela média do custo unitário do fármaco disponibilizado na REMUME para urgência hipertensiva, sendo eles: Hidralazina cloridrato 20 mg/mL, Nitroprussiato de sódio 25 mg/mL, Furosemida 10 mg/mL, Captopril 25mg e Clonidina 0,15mg. Para os medicamentos de administração endovenosa, foi considerado o custo da solução fisiológica 0,9% + equipo, sendo o custo obtido pela Divisão de Farmácia da SMS-RP e também o custo da administração do medicamento, obtido pela tabela unificada SUS (BRASIL, 2015b).

Quanto aos medicamentos AH, o custo unitário foi adquirido do relatório de aquisição da Divisão de Farmácia da SMS-RP. Esse custo foi dividido pela quantidade em miligramas correspondentes a cada medicamento para obter o custo / miligramas. Para o cálculo foi considerado o consumo anual em miligramas de cada medicamento anti-hipertensivo por paciente. O consumo foi multiplicado pelo custo em miligramas de cada medicamento utilizado por paciente para perfazer o custo de medicamentos AH por paciente/ano.

4.5.1.2.1. Custos das complicações da hipertensão arterial sistêmica

Para o cálculo do custo das complicações relativas à HAS, como AVE, DIC, DAP, IC e DRC foram considerados os custos com o Serviço Ambulatorial Móvel de Urgência (SAMU) por atendimento, o custo médio da hospitalização pela complicação e o custo anual do tratamento pós o evento com a reabilitação pelo SUS. Esses custos incluíram os custos totais, incluindo profissional, hospitalar, insumos farmacêuticos, medicamentos, exames, procedimentos, valor do acompanhante, Serviço de Apoio à Diagnose e Terapia - SADT e os custos com a unidade de tratamento intensivo – UTI, quando necessário. O custo por atendimento do SAMU pelos dados discriminados dos custos municipais pela Divisão de Finanças e de Custo Operacional da SMS-RP, e o número de atendimentos pelo Departamento de Informática, Estatística, Controle e Auditoria da SMS-RP. Foi realizada a média do custo total com o total de atendimentos para obter o custo do SAMU por atendimento. Os custos da internação foram obtidos pelo sistema de informação de internações hospitalares – SIH. Esse sistema integra a base de dados do TabWin do

Departamento de Informática do SUS, DATASUS (BRASIL, 2015g). Quanto aos custos com o tratamento pós-evento, os custos das DCVs, AVE, DIC e DAP foram obtidos pela estudo de Neyeloff (2012) o qual avalia economicamente essas três morbidades e calcula o custo médio do tratamento após o evento, considerando a reabilitação pelo SUS. O custo médio do tratamento da IC foi obtido pelo estudo de Araújo & Oliveira Jr. (2007) e o custo do tratamento com diálise da DRC obtido pela Divisão de Farmácia da SMS-RP. As diretrizes brasileiras de HAS, IC, dislipidemias e os estudos sobre DRC foram seguidos para a interpretação e correta análise do custo final de cada complicação (BOCCHI et al., 2009; SBC, 2010; BASTOS & KIRSZTAJN, 2011; XAVIER et al., 2013).

4.5.1.3. Custos indiretos

Os custos indiretos foram elencados como a perda de produtividade, custo devido invalidez e o custo por morte, esses foram calculados com base na taxa de pacientes hipertensos ativos, trabalhadores, e o rendimento médio real do brasileiro divulgado pelo IBGE (FERREIRA, COSTA, SOUZA, 2011; BRASIL, 2015d). Para ambos os custos foram considerados o valor de encargos sociais como INSS, salário-educação, seguro de acidentes do trabalho, contribuições (encargos repassados ao Sesi/Sesc/Sest, Senai/Senac/Senat, Incra e Sebrae), que chegam a totalizar de 18,8 a 27,8% do salário bruto, dependendo da contribuição do INSS. Foi considerando para o cálculo do salário bruto a média salarial dos pacientes. Ressalta-se que o salário bruto ainda inclui 8% do FGTS e que o valor máximo do INSS do segurado empregado é de R$ 513,01. A média foi ponderada pelas taxas de trabalhadores autônomos e não autônomos para a realização do cálculo da contribuição ao INSS e, consequentemente, calcular os encargos sociais do contribuinte (BRASIL, 2015e, BRASIL, 2015f; BRASIL 2006).

Para o cálculo do custo da perda de produtividade foi considerado a aposentadoria por invalidez, 100% do rendimento médio entre os pacientes que participaram deste estudo com acréscimo de 25% assegurado pelo INSS para o auxílio de um cuidador (BRASIL, 2014a; BRASIL, 1991). Esse montante foi somado ao valor dos encargos sociais que o paciente deixaria de contribuir. Em adição, foi acrescido o custo da complicação da HAS e o total foi multiplicado pelo número de pacientes que apresentaram complicações da doença e que permaneceram em estado estável. Para o cálculo do custo por morte foi considerado o custo da pensão por morte, somado ao valor dos encargos sociais que o paciente deixaria de contribuir. Em adição, a pensão foi estabelecida como vitalícia neste estudo, o determinado

para indivíduos acima de quarenta e quatro anos. Além disso, como o rendimento médio entre os pacientes que participaram deste estudo não ultrapassou o limite estabelecido da pensão por morte, este valor médio foi considerado como valor para a pensão (BRASIL, 2015f; BRASIL, 2015h). Esse custo foi atribuído aos pacientes que vieram a óbito por motivo de DCV e DRC. O salário bruto foi calculado da seguinte forma: salário bruto = salário ÷ (1-alíquota total de impostos). O valor de encargos sociais foi a diferença entre o salário bruto e a média salarial do paciente.

4.5.1.4. Custo da Atenção Farmacêutica

O custo com a ATF foi estimado pelo custo do profissional, adicionado ao custo para a estruturação do serviço (utensílios e materiais), e o custo básico da alocação do profissional em uma unidade de saúde para o atendimento dos pacientes. Para o custo do profissional foram consideradas as taxas conforme a descrição da folha salarial da SMS-RP para os profissionais contratados, como: adicionais por tempo de serviço, sexta parte dos vencimentos e progressões estabelecidas no Artigo 46 da Lei Municipal 2515/12. O vencimento foi considerado o piso salarial do farmacêutico para o estado de São Paulo, no ano de 2013 (ANEXO I). Os custos para a estruturação do serviço, como utensílios e materiais, foram estimados de acordo com os gastos do programa de ATF avaliado por este estudo. O custo com a alocação do farmacêutico para o atendimento foi estimado pela a média por atendimento do custo basal das UBDS e das UBS do município. Para o cálculo foram consideradas oito horas semanais trabalhadas no dia, um mês de férias, e vinte dias trabalhados no mês (descontado feriados e finais de semana). Sendo previsto o atendimento de 104 pacientes no mês, conforme o modelo de ATF deste estudo.

4.5.2. Primeira etapa: análise direta dos custos

Os custos foram tabulados, conforme sua discriminação, por ano e por período. Ressalta-se que para calcular a média do desfecho (controle pressórico por ano) foram considerados aqueles pacientes que tinham medidas para todos os anos do estudo. Quanto aos desfechos por período, foram considerados os pacientes que tinham medidas para os três períodos. A razão benefício-custo (RBC) foi calculada pela expressão RBC= benefício / custo. A razão de custo-efetividade (RCE), que representa o custo para cada unidade de desfecho adquirida, e a razão de custo efetividade-incremental (RCEI), que representa o custo para cada unidade a mais de desfecho adquirida, foram calculadas como RCE = custos ÷

desfechos e RCEI = Δ custos ÷ Δ desfechos. O benefício líquido incremental (BLI), interpretado como o benefício monetário para cada unidade a mais de desfecho adquirida foi utilizado para a sensibilidade das análises de custo efetividade. Ele foi calculado pela expressão BLI = (disposição a pagar pela ATF x Δ desfechos) / Δ custos. Sendo o BLI utilizado para a análise de sensibilidade dos resultados nesta etapa. Para isso, foi considerado o custo com a ATF como disposição a pagar por esse serviço e três vezes o valor do PIB nacional *per capita* anual (BRASIL, 2009; NITA et al., 2009; RASCATI, 2010).

4.5.3. Segunda etapa: análise indireta dos custos com a projeção das complicações da hipertensão através da modelagem de Markov

Para projetar as complicações da HAS foi utilizado um modelo de árvore de decisão. Foram elaborados três modelos, um para as DCVs de maior incidência (AVE, DIC, DAP) (WHO, 2011), outro para IC e outro para DRC, doenças que possuem a HAS como fator de risco associado à sua incidência (SBC, 2010). Os modelos elaborados foram importantes para a aplicação da modelagem matemática de Markov (RASCATI, 2010; CHEN et al., 2013). Com a modelagem foi possível atribuir estados de saúde para os três modelos. Os estados de saúde definido para o primeiro modelo foram HAS, incidência de cada complicação cardiovascular, prevenção secundária e morte. Para o modelo de IC foram definidos como estados de saúde HAS, tratamento IC e morte. Para o modelo de DRC foram definidos HAS, diálise e morte como os estados de saúde. Foram atribuídos nós de probabilidades com as respectivas probabilidades dos pacientes se alocarem a cada estado de saúde. Essas probabilidades foram calculadas com base nas taxas encontradas na literatura, com exceção da probabilidade calculada para a incidência de DCV que foi obtida pela taxa do risco cardiovascular em dez anos calculada pela escala de risco *ASCVD* para os pacientes deste estudo (MILLER & HOMAN, 1994; FLEURENCE & HOLLENBEAK, 2007). Esses modelos foram importantes para a projeção de alocação dos pacientes a cada estado de saúde no horizonte do tempo (DRUMMOND et al., 2005; BRASIL, 2009; RASCATI, 2010; STEVANOVIC, POSTMA, PECHLIVANOGLOU, 2012).

A mortalidade para cada complicação da HAS foi considerada de acordo com a faixa etária da população do estudo. Foi realizada uma média ponderada pelo percentual de homens e mulheres deste estudo para o cálculo das probabilidades de alocação no estado de saúde e morte, visto que a mortalidade varia entre ambos os sexos (ROSAMOND et al., 2008). Segundo Pinto e Mandil (2005) a forma letal da DAP pode ser considerada como a DAOP,

para isso foi utilizado a taxa de mortalidade por DAOP para o cálculo da probabilidade. Todavia, para o cálculo dos custos, foram considerados como custo do tratamento o custo da DAP e custo da incidência como o custo da DAOP. Para o cálculo das probabilidades de recorrência foi utilizado a taxa de incidência entre os indivíduos que já possuíam a doença. Ressalta-se que não foram consideradas reincidência para IC e DRC, pois são doenças em que o prognóstico prevê a agudização do quadro e não um novo acometimento (HANRATTY et al., 2011; NOGUEIRA et al., 2010).

Dessa forma, a árvore iniciou em um nó de decisão, que representou os pacientes com atendimento da ATF e sem o atendimento da ATF, por se tratar do estudo antes e depois, sendo considerado antes de 2009 como sem o atendimento e, em 2009 como o com atendimento. A probabilidade de apresentar as complicações por DCV para o primeiro modelo foi estabelecida pela média do percentual da escala de risco *ASCVD*, calculado para os pacientes do estudo. Essa medida de risco projetou em dez anos o percentual de risco da ocorrência de complicações cardiovasculares para os pacientes. Foi um instrumento preferido diante de outros, por considerar também a etnia do paciente e utilizar as variáveis para estimar o risco para as doenças cardiovasculares e não somente o risco coronariano (DAWBER, MOORE-Jr, MANN, 1957; LOTUFO, 2008; STONE et al., 2013; MUNTNER et al., 2014).

Diante disso, o nó de Markov, no qual se inicia a modelagem, dividiu-se nos estados de saúde que se ramificaram em probabilidades até o nó terminal. Adicionalmente, os pacientes entraram na modelagem e foram alocados a cada ano em um estado de saúde, de acordo com o risco cardiovascular e as probabilidades calculadas. Pela diferença do número de pacientes alocados no estado de óbito e na prevenção secundária, foram calculados respectivamente os anos de vida ganhos (AVG) e os anos de vida ajustados pela qualidade (AVAQ), os quais foram relacionados ao desfecho para as análises de custo efetividade (DRUMOND et al., 2005).

Para cada estado de saúde foi atribuído o respectivo custo: custos da incidência da complicação mais o tratamento para os pacientes que se alocaram no estado de prevenção secundária; custo do tratamento da complicação para aqueles que permaneceram no estado de prevenção secundária; o custo da incidência da complicação mais o custo de morte para os pacientes que se alocaram no estado de morte, após apresentar a complicação da doença; e o custo de morte para aqueles que estavam no estado de morte. O custo da incidência e tratamento foi calculado pelo somatório dos custos com o SAMU, custo médio da

hospitalização, custo do tratamento anual pós-evento e custo indireto de pensão por invalidez. Ressalta-se que foi considerada a taxa de invalidez para as complicações da HAS no cálculo desse custo, obtida dos estudos de Moraes & Freitas (2012), Araújo et al. (2010), Neyeloff (2012) e Hanratty et al. (2011). O custo do tratamento foi calculado pelo somatório desses mesmos atributos, porém sem o custo da hospitalização. O custo da incidência e morte foi calculado pela soma dos custos do SAMU, custo médio da hospitalização e custo indireto de pensão por morte.

O primeiro modelo para as DCVs foi um modelo de estrutura mais complexa por existir a possibilidade de reincidência das complicações e também por ramificar nas DCVs (AVE, DIC e DAP). Essa ramificação teve como base os nós de probabilidades proporcionais à prevalência dessas morbidades. No entanto, as modelagens para IC e DRC foram de estrutura mais simples por serem morbidades em que não ocorre a reincidência e não se ramificam em outras morbidades.

Para o cálculo das probabilidades de prevalência, morte e reincidência do AVE, DIC e DAP foram utilizadas as taxas segundo os estudos de Mansur et al. (2012), Garritano et al. (2012), Rosamond et al. (2008) e Gismondi et al. (2007) para a AVE e DIC. Para a DAP os estudos de Fowkes, (2013), Aragão et al. (2009) e Pinto e Mandil (2005). Quanto a IC e DRC não foram extraídas taxas de prevalência e sim incidência em pacientes com PA descontrolada, pois a escala de risco *ASCVD* não pode ser utilizada para o cálculo da incidência dessas doenças. Dessa forma, os estudos de Nogueira et al. (2010) e a diretriz de IC por Bocchi et al. (2009) foram usados para as taxas de incidência e morte por IC, e os estudos de Hanratty et al. (2011), Bortolotto (2008), Batista et al. (2005) e Siviero (2014) para a DRC. O risco relativo foi calculado com os dados desses estudos para então proporcionar o cálculo de probabilidade da morte e a incidência.

Para a modelagem foram considerados os números de ciclos de Markov que remetem a dez anos, o que é referente à projeção de risco da escala *ASCVD*. Nesses dez anos não foi atribuído o risco de morte natural, pois para a análise de custo foi entendido que os custos deveriam restringir-se à HAS e às complicações da doença para estimar a diferença entre os custos do modelo assistencial convencional do SUS e o modelo com a ATF incorporada. O método utilizado foi de simulação de coorte, onde os pacientes entram todos com uma mesma medida e são alocados nos diferentes estados de saúde a cada ciclo. Para isso, foi simulada a coorte de 104 pacientes, os quais participaram do programa de ATF (RASCATI, 2010;

DRUMMOND, et al., 2005). Para a estruturação e análise desta etapa do trabalho foi utilizado o *software Data TreeAge Pro®* 2011.

4.5.4. Terceira etapa: realização da análise de custo-benefício em longo prazo pelo modelo de fluxo de caixa

Esta etapa do estudo utilizou os custos da análise direta e indireta, os quais formaram um modelo de fluxo de caixa anual para a obtenção do VPL. O VP foi ajustado pela taxa através do método do *discounting*. A taxa de *discounting* para o ajuste temporal dos custos foi considerada conforme a recomendação da Rede Brasileira de Avaliação de Tecnologia em Saúde - REBRATS, a taxa de 5% anual (BRASIL, 2009). Com isso foi possível trazer as economias referentes à diferença dos custos entre a assistência com a ATF e a assistência convencional prometidas no futuro para o valor presente. Esse método aplicado no modelo de fluxo de caixa permitiu obter o benefício relacionado ao programa de ATF e aplicar a análise de sensibilidade dos custos ao longo de dez anos (DRUMOND et al., 2005; NETO & LIMA, 2009; RASCATI, 2010; OLIVEIRA, 2013). Adicionalmente, foi possível calcular a Taxa de retorno de investimento relacionada ao programa de ATF (Figura 4).

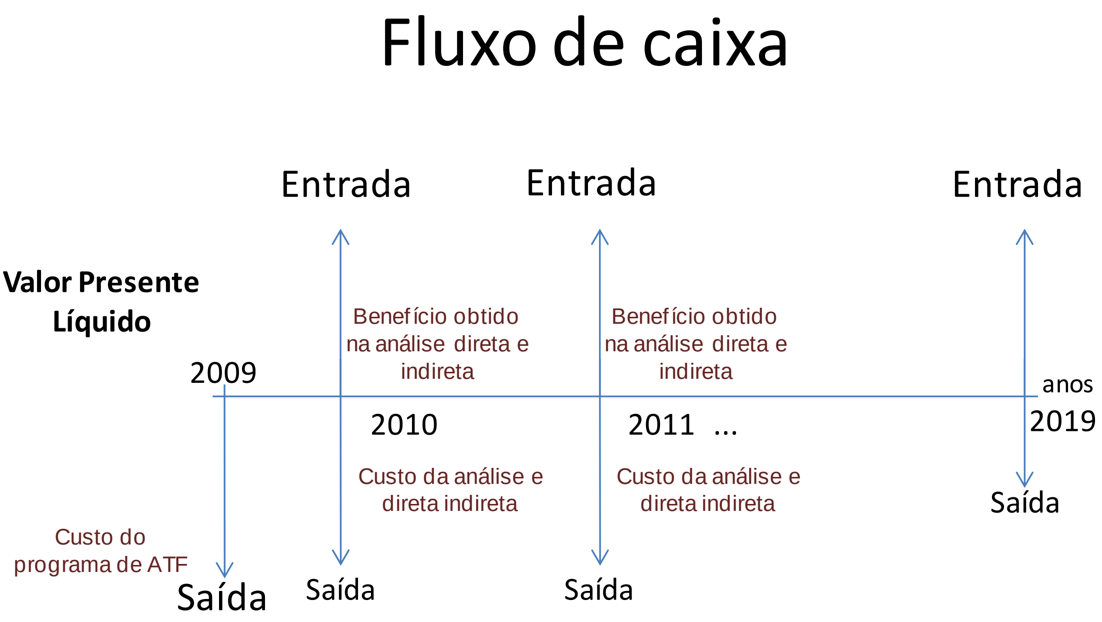

Legenda Figura 4: ATF = Atenção Farmacêutica

Figura 4 – Modelo de fluxo de caixa para a obtenção do valor presente líquido.

4.5.5. Quarta etapa: análise de sensibilidade dos custos para a elaboração da ferramenta farmacoeconômica

A interferência da variação dos custos nos resultados foi mensurada por meio da análise de sensibilidade realizada pelo *software @Risk*® versão 11, modo acadêmico. Os custos foram variados de acordo com os percentuais de variação atribuídos a cada categoria de custo ou pelos valores mínimo e máximo de cada categoria de custo. Para isso, foi considerado a distribuição dos dados relativos a cada categoria de custo e também os valores encontrados na literatura. Para as categorias de custo em que isso não foi possível, foi considerada a variação de 50% na amplitude (BRASIL, 2009; RASCATI, 2010).

Dessa forma, a influência da variação das diferentes categorias de custo no VPL foi calculada por 10.000 iterações pela simulação de Monte Carlo. A sensibilidade foi verificada quanto à densidade de probabilidades para a variação do custo e quanto ao diagrama de tornado para mensurar o grau de influência de cada categoria de custo no VPL. Foi considerada a probabilidade de 95% para análise do VPL, entendido como um intervalo de confiança.

4.5.6. Quinta etapa: impacto epidemiológico da Atenção Farmacêutica

Nesta etapa os indicadores epidemiológicos foram estimados quanto à associação entre a assistência à saúde sem a ATF, considerada como um fator de exposição para a PA em nível insatisfatório e o surgimento das complicações da HAS. O controle pressórico insatisfatório e as complicações da HAS foram entendidos como fatores doença e interpretados como resultado indesejável. O total de pacientes com o nível da PA satisfatório e insatisfatório foi mensurado no estudo clínico e a proporção de indivíduos com e sem complicações da doença, mensurada na segunda etapa deste estudo farmacoeconômico.

O *Odds Ratio* (OR) e o Risco Relativo (RR) podem ser estimados em estudos clínicos para variáveis categóricas e neste estudo foram calculados na análise de contingência entre os períodos Pré ATF e Pós ATF, (FRANCO & PASSOS, 2005; PAGANO & GAUVREAU, 2012). A tabela 2x2 mostra como foi estruturada a análise (Tabela 1). O *software Statistical Package for Social Sciences*® (SPSS) v.16 foi utilizado para realizar a análise de contingência utilizando o chi-quadrado para um grau de liberdade, considerando o nível de significância de 5% para o teste exato de Fisher. O calculo do percentual de Redução do Risco Relativo, ou eficiência da ATF, foi realizado da seguinte forma: (inverso do RR – 1) x 100.

Para calcular a RRR houve a necessidade de inverter o risco relativo devido a estrutura da análise estar avaliando a ausência da ATF como fator de exposição para os pacientes não controlarem a PA ou apresentarem as complicações da HAS.

Tabela 1 - Tabela 2x2 para análise epidemiológica

	Número de pacientes com resultado indesejável	Número de pacientes com resultado desejável	Total
**Pré ATF**	**A**	**B**	**A+B**
**Pós ATF**	**C**	**D**	**C+D**
**Total**	**A+C**	**B+D**	**N**

FONTE: adaptada de Franco e colaboradores (2005).

Legenda Tabela 1: ATF= Atenção Farmacêutica; n compreende o total de indivíduos expostos e não expostos; os indivíduos expostos e não saudáveis foram aqueles com resultado indesejável e sem Atenção Farmacêutica (A); indivíduos expostos e saudáveis foram aqueles com resultado desejável e sem Atenção Farmacêutica (B); indivíduos não expostos e não saudáveis foram aqueles com resultado indesejável e com Atenção Farmacêutica (C); indivíduos não expostos e saudáveis foram aqueles com resultado desejável e com Atenção Farmacêutica (D).

4.5.7. Elaboração da ferramenta de análise econômica

Com a aplicação da modelagem de Markov foi possível obter o número de pacientes que não apresentaram as complicações da HAS devido serem assistidos pela ATF. Dividindo o total de pacientes por esse número foi obtido o Número Necessário para Tratar (NNT), que representa quantos pacientes foram necessário receber essa intervenção farmacêutica para se evitar uma complicação (COUTINHO & CUNHA, 2005).

A RRR foi utilizada para calcular o número de pacientes que apresentaria a PA em níveis satisfatório ao longo de dez anos de análise. Para o valor de investimento no programa de ATF, foi considerado o custo do limiar superior relativo ao intervalo de confiança de 95% e para os benefícios anuais adquiridos pelas complicações da HAS evitadas, foi considerado o limiar inferior do intervalo de confiança de 95%.

Dessa forma, a ferramenta farmacoeconômica foi desenvolvida no *software* Excel®. Ela foi projetada para conseguir interpretar o valor a ser investido pelo gestor de saúde e calcular o número de farmacêuticos a contratar, o número de complicações da HAS a ser evitada, o número de pacientes que o programa de ATF conseguirá atender, o número de pacientes com a PA em níveis satisfatórios e como resultado final o VPL de acordo com o valor que o gestor de saúde tem para investir. Ressalta-se que foi considerado o valor mínimo para o retorno do investimento no limiar de 95%.

4.6. Comitê de Ética em Pesquisa

O presente estudo teve sua aprovação pelo Comitê de Ética em Pesquisa da FCFRP-USP em 10 de Fevereiro de 2014, com a liberação de aprovação nº 004/2014 e protocolo CEP/FCFRP nº324, CAAE 21162713.8.0000.5403 (http://plataformabrasil.saude.gov.br). O programa de ATF, desenvolvido e realizado pelo CPAFF (PENAFORTE, 2011), teve sua aprovação pelo Comitê de Ética em Pesquisa do Centro de Saúde Escola da Faculdade de Medicina de Ribeirão Preto, documento 664/07/COORD, CEP/CSE-FMRP-USP-12/12/2007, protocolo Nº. 256/CEP-CSE-FMRP-USP e liberação 054/2007 (ANEXO II). Ressalta-se que este estudo teve o apoio da agência financiadora Fundação de Amparo à Pesquisa do Estado de São Paulo (FAPESP) pelo fomento a pesquisa com o auxílio da bolsa de pesquisa (Mestrado - Fluxo Contínuo, processo nº 2014/02087-9), concedida para o período relacionado de 01/08/2014 a 15/10/2016. Contudo, as opiniões, hipóteses e conclusões ou recomendações expressas neste material são de responsabilidade do autor e não necessariamente refletem a visão da FAPESP.

5. RESULTADOS E DISCUSSÃO

5.1. Perfil dos pacientes hipertensos

Um problema atribuído aos estudos com humanos em longo prazo é a taxa de evasão e ausência de dados durante o tempo. Segundo a literatura há evasão estimada de 40-56% dos pacientes em estudos de acompanhamento farmacoterapêutico (AGUWA, UKWE, EKWUNIFE, 2008; LYRA, MARCELLINI, PELÁ, 2008). Dos 191 pacientes que aceitaram participar do programa de ATF, 104 permaneceram no acompanhamento até o final, completando as doze consultas, o que representa uma descontinuidade de 45,5% dos pacientes. Não obstante, nem todos os pacientes possuíam dados para os indicadores a serem analisados no estudo, principalmente para os anos após a alta. Desta forma, os dados foram estratificados (Figura 5).

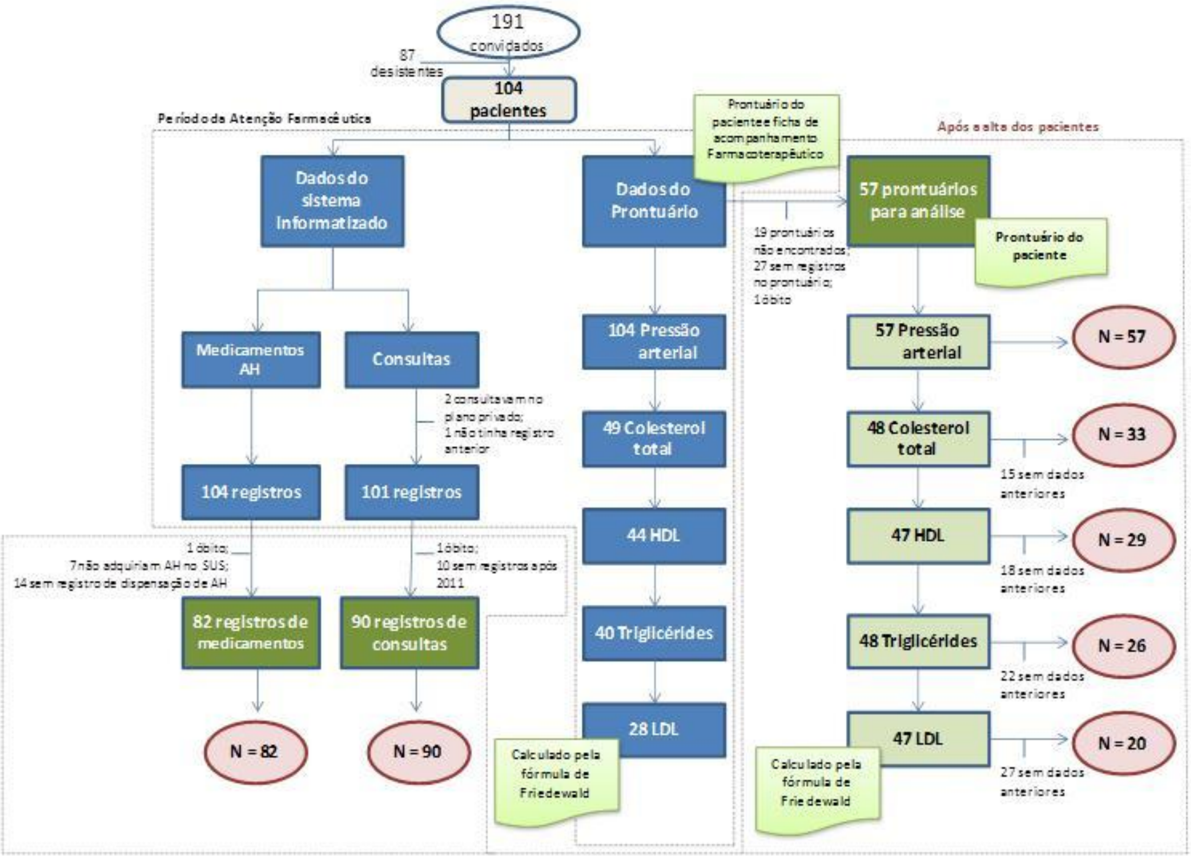

Legenda Figura 5: ATF = Atenção Farmacêutica; AH = Anti-hipertensivo; SUS = Sistema Único de Saúde; LDL = Lipoproteína de baixa densidade; HDL = Lipoproteína de alta densidade; O LDL era registrado nos prontuários por meio de cálculo através da fórmula de *Friedewald*, LDL=(CT-HDL)-(TG/5) e, quando possível, foi calculado dessa forma para os prontuários em que esse dado era faltante.

Figura 5 – Fluxograma da estratificação do tamanho amostral por indicador a ser analisado.

É possível notar que os 104 pacientes que participaram do programa de ATF perfazem o número amostral necessário, permanecendo dentro do intervalo calculado no planejamento amostral. Ainda, para a variável de interesse do estudo, pressão arterial, o número de 57 pacientes também está dentro desse intervalo. Nesse sentido, os resultados deste estudo foram provenientes de uma amostra que faz inferência a uma população de hipertensos com o perfil de risco cardiovascular e sociodemográfico apresentados na Tabela 2.

Tabela 2 – Perfil dos pacientes hipertensos acompanhados pela Atenção Farmacêutica no início do estudo

Perfil Sociodemográfico			Perfil de Risco Cardiovascular			
	n (104)	%			n (104)	%
**Sexo**			**Obesidade** *(IMC ? 30 Kg/m²)	Sim	56	53,9
Masculino	26	25,0		Não	48	46,2
Feminino	78	75,0				
**Idade**						
30-40 anos	2	1,9	**Dislipidemia**	Sim	28	26,9
41-59 anos	35	33,7		Não	76	73,1
>60 anos	67	64,4				
**Cor da pele**						
Branca	58	55,8	**Antecedentes familiar**	Sim	14	13,5
Negra	8	7,7		Não	90	86,5
Parda	38	36,5				
**Escolaridade**						
Analfabeto	8	7,7	**Tabagismo**	Sim	5	4,8
Ensino fundamental	75	72,1		Não	99	95,2
Ensino médio	17	16,3				
Ensino superior	4	3,9				
**Atividade Ocupacional**	71	68,3	**Diabetes**	Sim	19	18,3
				Não	85	81,7
**Renda familiar**						
até 1 SM	7	6,7				
>1-2 SM	26	25,0				
>2-3 SM	29	27,9	**Circunferência abdominal** *	Alterada	84	80,8
>3-5 SM	32	30,8		Não alterada	20	19,2
>5 SM	10	9,6				

Legenda Tabela 2: *Fonte: VI diretrizes Brasileiras de Hipertensão, 2010; Foi considerado como alterado os valores de circunferência abdominal superiores a 102 cm para homens e 88 cm para mulheres (SBC, 2010); SM = salário mínimo; a renda familiar foi classificada de acordo com Instituto Brasileiro de Geografia e Estatística – IBGE.

Foi estimado pela amostra deste estudo, pacientes com um perfil sociodemográfico prevalente do sexo feminino, idosos, cor da pele branca, apenas com o ensino fundamental concluído, em atividade ocupacional e pertencente à classe média baixa. Adicionalmente, observa-se um perfil de risco cardiovascular que justifica a prevenção, sendo que mais da metade dos pacientes foi classificado como obeso e que 80,8% possuíam a circunferência abdominal acima do valor recomendado. Destaca-se, neste estudo, a maior prevalência da HAS a partir da quinta década de vida para o sexo feminino e também para a raça branca, apesar desta morbidade ser mais prevalente na raça negra de acordo com a literatura, ela é

considerada uma doença de etiologia multifatorial. Em adição, é possível ressaltar que os fatores como o nível educacional e a renda são preponderantes para as características étnicas quanto à prevalência da HAS no Brasil, pois são determinantes ao acesso à saúde e à educação em saúde (SBC, 2010, BRASIL, 2013a). Dessa forma, a amostra deste estudo tem a característica de um perfil de pacientes representativo da população hipertensa no Brasil. Assim, torna-se possível extrapolar os resultados alcançados pela ATF quanto aos indicadores clínicos, assistenciais e ao percentual de risco cardiovascular.

5.2. Resultados clínicos e assistenciais

Dos 57 pacientes que tiveram os registros dos indicadores de PAS e PAD durante os períodos de pré ATF, ATF e pós ATF, 31 (54,4%) e 45 (79,0%) apresentaram, respectivamente, essas parâmetros satisfatórios no período pré ATF. No período ATF esse número aumentou para 56 (98,2%) dos pacientes, se sustentando após a alta da ATF em 53 (93,0%), apresentando a estatística Q igual a 43,35 e 85,85, respectivamente. Isso mostrou evidência da ATF estar associada ao controle pressórico em níveis satisfatórios mesmo após a alta dos pacientes. O mesmo foi verificado para o CT, apresentando o valor da estatística Q igual a 14,92 (Tabela 3).

Tabela 3 – Proporção de pacientes com os resultados clínicos satisfatórios para os três períodos de análise dos resultados

Indicadores clínicos	*n*	**Pré ATF**<br>Satisfatório (%)	**ATF**<br>Satisfatório (%)	**Pós ATF**<br>Satisfatório (%)	valor *p*
**PAS**	*57*	31 (54,4%)	56 (98,2%)*	53 (93,0%)*	<0,001
**PAD**	*57*	45 (79,0%)	56 (98,2%)*	53 (93,0%)*	<0,001
**TG**	*26*	14 (53,8%)	11 (42,3%)	15 (57,7%)	0,236
**CT**	*33*	9 (27,3%)	17 (51,5%)*	20 (60,6%)*	<0,001
**LDL**	*20*	8 (40,0%)	11 (55,0%)	11 (55,0%)	0,325
**HDL**	*29*	6 (20,7%)	8 (27,6%)	5 (17,2%)	0,882

Legenda Tabela 3: * Estatística Q para a distribuição chi-quadrado > 5,99 (ponto crítico para rejeitar a hipótese nula); PAS= Pressão arterial sistólica; PAD= Pressão arterial diastólica; TG= Triglicerídeos; CT= Colesterol total; LDL= Lipoproteína de baixa densidade; HDL= Lipoproteína de alta densidade.

A ATF é capaz de abordar o indivíduo como um todo em suas especificidades biopsicossociais envolvendo, para isso, ações de educação em saúde. Apesar de utilizar a farmacoterapia no planejamento das suas ações, a ATF não se restringe apenas aos efeitos farmacológicos. Ressalta-se que são realizadas orientações e intervenções não farmacológicas empoderando o paciente para o seu cuidado, melhorando a adesão terapêutica e a qualidade de vida (OPAS, 2002). O programa de ATF abordado neste estudo pode exemplificar isso, pois a ATF foi capaz de promover 100% da adesão à farmacoterapia, sendo que no período pré ATF

54% dos pacientes não eram aderentes. Em adição, houve o aumento do *escore* nos oito domínios do instrumento de qualidade de vida SF-36 (capacidade funcional, aspectos físicos, dor, estado geral de saúde, vitalidade, aspectos sociais, aspectos emocionais e saúde mental), representando um aumento médio de 66,8 para 85,3 pontos, o que representa 21,7% de melhora na qualidade de vida desses pacientes.

Em adição, o programa de ATF teve como resultado a modificação no perfil assistencial dos pacientes. Fator que pode ser verificado no indicador consulta, pois houve evidência do aumento na média anual de consultas por paciente no atendimento básico, durante o período pós ATF, o que também pode ser verificado ao analisar o intervalo de confiança (IC). Destaca-se que após a ATF a média das consultas se aproximou mais da mediana, e que a mediana esteve mais próxima do terceiro quartil, o que mostra que mais de 50% dos pacientes tiveram a média de consultas no atendimento básico aumentado nesse período. Adicionalmente, no período pré ATF, 25% dos pacientes tiveram média de consultas no pronto atendimento igual ou menor que um e, nos períodos posteriores, esse percentual passou para 50%. Ressalta-se com isso que houve maior proporção de pacientes com o número de consultas igual ou menor que uma consulta no pronto atendimento, sendo evidente a redução para os períodos ATF e Pós ATF (Tabela 4).

Tabela 4 – Medidas de resumo e dispersão relativas às consultas e ao consumo de medicamentos anti-hipertensivos para os três períodos de análise dos resultados

Indicadores Assistenciais	**Pré ATF**		**ATF**		**Pós ATF**		
	Mediana (IQ)	Média ± DP (IC 95%)	Mediana (IQ)	Média ± DP (IC 95%)	Mediana (IQ)	Média ± DP (IC 95%)	valor *p*
**Consultas** (paciente/ano)							
Atendimento Básico	1,50 (0,00; 3,00)	1,66±1,43 (1,36; 1,96)	2,00 (0,00; 3,00)	2,02±1,74 (1,66; 2,39)	2,30 (0,00; 3,30)	*2,36±1,73 (2,00; 2,72)	0,012
Atendimento Especializado	0,00 (0,00; 1,00)	0,60±0,93 (0,40; 0,80)	0,00 (0,00; 1,00)	0,54±0,86 (0,36; 0,72)	0,00 (0,00; 1,00)	0,48±0,86 (0,30; 0,66)	0,238
Pronto Atendimento	1,70 (1,00; 2,47)	1,70±1,38 (1,43; 2,00)	1,00 (0,00; 2,00)	*1,17±1,29 (0,90; 1,43)	1,00 (0,30; 1,30)	*1,06±0,81(0,89; 1,23)	0,002
**Medicamentos AH** (mg/dia) ajustado	30,00 (18,00; 48,00)	33,95±20,84 (29,37; 38,53)	30,00 (18,00; 42,00)	36,68±27,82 (30,56; 42,79)	30,00 (18,00; 42,00)	35,7±24,42 (30,34; 41,07)	0,507

Legenda Tabela 4: AH = anti-hipertensivo; ATF = Atenção Farmacêutica; * = diferença estatística detectada pelo teste de hipótese para a comparação com o período pré ATF; IQ = intervalo interquartil; DP = desvio padrão. O ajuste para os medicamentos anti-hipertensivos foi realizado conforme o Quadro 1.

É importante destacar que neste estudo não houve evidência da ATF estar associada ao aumento da utilização dos medicamentos AH. Embora outros estudos que avaliaram a ATF terem mostrado o aumento do consumo de medicamentos de forma geral, quando foi analisado o consumo de medicamentos específicos ao tratamento da morbidade a que o programa de ATF se destina, não houve evidência para o aumento do consumo, conforme neste estudo (BORGES et al., 2011; LYRA-JUNIOR, MARCELLINI, PELÁ, 2008).

No período pré ATF o percentual médio de risco cardiovascular dos pacientes hipertensos foi 19,5% ± 16,9. No período ATF a média do percentual de risco reduziu para 11,81% ± 8,6, mantendo em 13,59% ± 8,9 após a alta dos pacientes. Ao analisar o risco cardiovascular pertinente aos fatores de risco modificáveis, verifica-se que as médias foram 12,61% ± 11,84; 5,14 ± 4,8 e 5,15% ± 4,10, para os três períodos, respectivamente. Adicionalmente, o teste de hipótese apresentou estatística de $F(2,27) = 9{,}14$ [$p=0{,}0004$] quando considerado todas as variáveis de fatores de risco e $F(2,27)= 15{,}44$ [$p<0{,}0001$] considerando apenas as variáveis de fatores de risco modificáveis. No pós-teste entre grupos o valor de *p* foi menor que 0,05 na comparação dos períodos ATF e pós ATF com o período pré ATF (Figura 6).

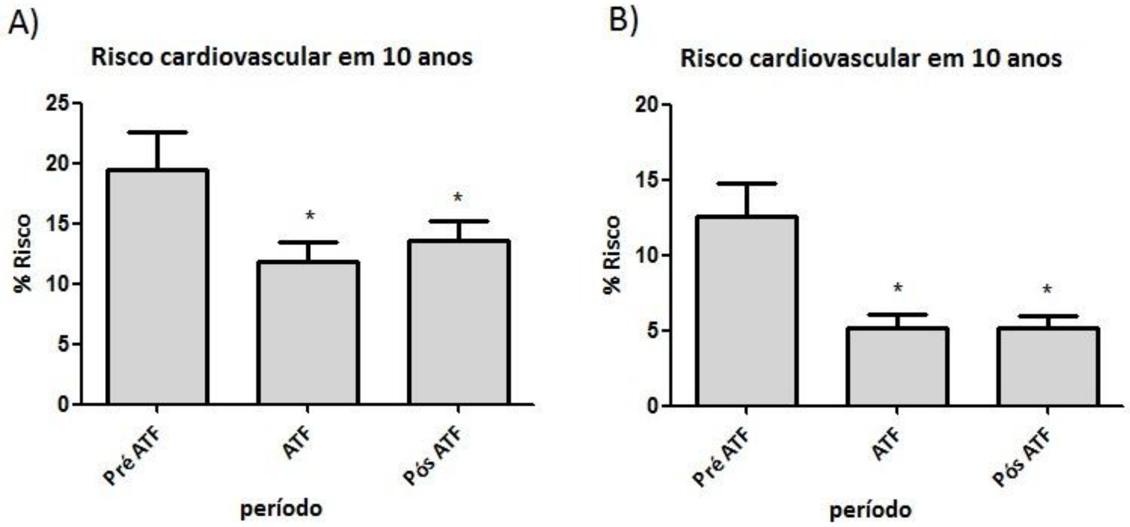

Legenda Figura 6: A parte **A)** desta figura representa o gráfico do percentual de risco cardiovascular atribuído a todas as variáveis consideradas fatores de risco. A parte **B)** da figura representa o gráfico do percentual de risco cardiovascular calculado com base nas variáveis que representam os fatores de risco modificáveis.

Figura 6 -Percentual de risco cardiovascular em dez anos calculado pela escala *ASCVD*.

Houve evidência para a associação da ATF à redução de 30,3% na média de risco cardiovascular, sendo também evidente a associação da ATF para à redução do risco se manter mesmo após a alta dos pacientes. Considerando que a ATF estaria atuando nos fatores de risco modificáveis é possível perceber que essa TS proporcionou a melhora desses fatores, reduzindo o risco cardiovascular pertinente a esses fatores praticamente na mesma proporção nos períodos ATF e pós ATF. Ainda, no período após a alta dos pacientes foi verificado um desvio padrão menor, o que prediz que os pacientes tiveram uma discrepância menor do risco cardiovascular em torno da média calculada, ou seja, mais pacientes com um risco menor.

5.3. Determinação e análise dos custos diretos

É importante destacar que dos 57 pacientes que tiveram os prontuários para a análise deste estudo, seis não tinham dados referentes aos indicadores assistenciais de consumo de medicamento anti-hipertensivo e das consultas. Dessa forma, os cálculos de custo foram atribuídos aos 51 pacientes, sendo o custo total anual calculado pela média, para cada variável de custo entre os 51 pacientes. Esse valor, que representa o custo médio anual por paciente relativo a cada variável de custo, foi multiplicado pelo total de pacientes atendidos pela ATF, ou seja, 104 pacientes, para obter o custo total anual. O custo total por período e o custo por paciente em cada período foram obtidos pela média entre cada ano do período.

O custo total da ATF, considerando este modelo com doze consultas farmacêuticas anuais por paciente, foi de R$ 51.518,60, o que representou R$ 495,37 / paciente e R$ 41,28 / atendimento (Quadro 2). Neste caso, foi considerada a capacidade de atendimento do farmacêutico igual a 104 pacientes / mês, pois foi o número máximo de pacientes que um farmacêutico foi capaz de realizar a ATF perfazendo as doze consultas anuais. O aumento desse número implicaria no aumento do consumo de materiais para a realização da ATF, mas, de forma geral, como o maior custo está implicado com o recurso humano, seria possível reduzir o custo por paciente e por atendimento (BRASIL, 2015m).

Quadro 2- Descrição para o cálculo dos custos da Atenção Farmacêutica

Descrição salarial	Taxas	Montante	ANUAL (com 13º salário)
Salario Base SP 40h		2.100,00	27.300,00
G.E.A. - Art. 1 LC 391/94	0,25	525,00	6.825,00
Insalubridade 20%	0,01675	35,18	457,28
Ad. Do Prêmio Incentivo	0,0835	175,35	2.279,55
Critério Assiduidade - LC 406/94	0,03	63,00	819,00
**TOTAL**		**R$ 2.898,53**	**R$ 37.680,83**
**Descrição do Custo da ATF**			**CUSTO**
Salario Anual			R$ 37.680,83
Materiais			R$ 1.963,05
Unidade para atendimento			R$ 11.874,72
**TOTAL**			**R$ 51.518,60**
Por paciente			R$ 495,37
Por atendimento			R$ 41,28

Legenda Quadro 2: Para os valores de vencimento foi considerado o salário base do farmacêutico no estado de São Paulo. As taxas de acréscimo foram proporcionais ao empenhado pela Secretaria de Saúde do Município de Ribeirão Preto. Os materiais foram os itens essenciais para estruturar um serviço de Atenção Farmacêutica para pacientes hipertensos. O custo total por paciente e por atendimento é respectivo ao período anual.

Os materiais julgados como necessários para a implantação de um serviço de ATF, para o atendimento de 104 pacientes/ano, estão descritos no Quadro 3 com seus respectivos custos.

Quadro 3- Descrição dos materiais necessários para estruturar o serviço de Atenção Farmacêutica para o atendimento de 104 pacientes

Atenção Farmacêutica - 104 paciente / 1 ano<br>Materiais necessários			
**DESCRIÇÃO**	**Quantidade**	**Valor Un.**	**Total**
**Mobilia**			
Mesa	1	135	R$ 135,00
Cadeira	3	60	R$ 180,00
Armário simples	1	180	R$ 180,00
**Material Escritorio**			
Papel sulfite c/500	10	8,65	R$ 86,50
Prancheta	2	2,8	R$ 5,60
Caneta	12	0,49	R$ 5,88
Pastas prontuário	104	1,25	R$ 130,00
Furador de papel	1	14,77	R$ 14,77
Grampeador	1	11,48	R$ 11,48
Tesoura	1	5,38	R$ 5,38
Grampo (caixa c/5000 un)	1	4,2	R$ 4,20
Clipes (caixa)	2	1,56	R$ 3,12
Régua	2	1,16	R$ 2,32
Lixeira	1	30	R$ 30,00
Caneta marca texto	5	2,2	R$ 11,00
**Materias Gerais**			
Computador	1	720	R$ 720,00
Impressora	1	230	R$ 230,00
**Materiais para aferição de medidas**			
Aparelho de pressão (aneróide com esfigmomanômetro)	1	160	R$ 160,00
Fita métrica	2	3,9	R$ 7,80
Balança digital	1	40	R$ 40,00
**TOTAL**			**R$ 1.963,05**

Legenda Quadro 3: Esses valores foram embasados no levantamento de custos para estruturação do serviço de Atenção Farmacêutica na Farmácia Ensino da Faculdade de Ciências Farmacêuticas de Ribeirão Preto – Universidade de São Paulo, em 2015. Valor Un. = valor unitário.

A média do custo do atendimento especializado nas UBDS foi de R$ 75,57 / atendimento. Calculando o custo do atendimento especializado para o paciente hipertenso a média foi de R$ 75,75. Foi verificado que para o pronto atendimento a média de custo da unidade de pronto atendimento foi de R$ 63,26, porém a média de custo / atendimento do paciente hipertenso foi de R$ 67,23. Quanto aos custos do atendimento básico, o qual teve a média ponderada pelo número de ESF e UBS, o total foi de R$ 106,24 / paciente hipertenso (Quadro 4).

Quadro 4 – Descrição dos custos para o cálculo dos custos das consultas no atendimento especializado, pronto atendimento e atendimento básico

Custos do Atendimento Especializado		Custos do Pronto Atendimento		Custo do Atendimento Básico	
**Acompanhamento médico**	Realizado a cada 2 anos				
Angiografia de Arco Aórtico (0210010029)	R$ 137,01	Custo Basal da Unidade de urgência	R$ 41,64	Média de custo/paciente UBS	R$ 118,32
Ultrassonografia Doppler (0205010040)	R$ 39,60				
Ecocardiografia transtoráxica(020501002)	R$ 39,94	Atendimento do Médico (0301010072)	R$ 10,00	Média de custo/paciente ESF	R$ 103,26
**Soma**	**R$ 216,55**				
**Média por consulta**	**R$ 36,09**	Atendimento de Enfermagem (0301010048)	R$ 6,30		
				Média ponderada	R$ 106,24
**Custo da consulta especialidade**		Administração de Medicamento (0301100012)	R$ 0,63		
Custo basal da UBDS/atendimento	R$ 14,31				
Custo do ECG (0211020036)	R$ 5,15	Medicamentos*	R$ 3,51		
Custo do acompanhamento médico	R$ 36,09				
Atendimento da enfermagem (0301010048)	R$ 6,30	Exame ECG (0211020036)	R$ 5,15		
Atendimento do cardiologista (0301010072)	R$ 10,00				
Outros exames de rotina	R$ 3,90				
**TOTAL custo por paciente**	**R$ 75,75**	**TOTAL custo por paciente**	**R$ 67,23**	**Total custo por paciente**	**R$ 106,24**

Legenda Quadro 4: Foi considerado a média de três consultas anuais por paciente no cálculo do custo do atendimento especializado; ( )= código da tabela SUS; *média de custos dos medicamentos usados na crise hipertensiva. Outros exames de rotina são: urina tipo I, potássio plasmático, creatinina sérica, ácido úrico e glicemia de jejum, foi ponderado seu custo anual.

Para os custos dos exames foi considerado o valor de R$ 3,15, valor esse atribuído pela tabela unificada SUS aos exames de CT, LDL, HDL e TG (BRASIL, 2015b). Quanto aos medicamentos, os custos unitários adquiridos pela divisão de farmácia do município foram ajustados para 2015 (Quadro 5).

Quadro 5 – Custo de aquisição dos medicamentos anti-hipertensivos pelo município de Ribeirão Preto

Medicamentos	mg	Valor unitário 2013	Valor Unitário 2015	Custo / mg em 2015		Valor por mg 2013	
Anlodipina	5	0,0187	0,022	R$	0,004400	R$	0,003740
Captopril	25	0,013	0,014	R$	0,000560	R$	0,000520
Carvedilol	12,5	0,075	0,063	R$	0,005040	R$	0,006000
Carvedilol	6,25	0,061	0,064	R$	0,010240	R$	0,009760
Clonidina	0,15	0,16	0,173	R$	1,153333	R$	1,066667
Enalapril	10	0,015694661	0,017962885	R$	0,001796	R$	0,001569
Enalapril	20	0,0198	0,044	R$	0,002200	R$	0,000990
Espironolactona	25	0,085	0,084	R$	0,003360	R$	0,003400
Espironolactona	100	0,25	0,199	R$	0,001990	R$	0,002500
Furosemida	40	0,018	0,018	R$	0,000450	R$	0,000450
Hidroclorotiazida	25	0,014	0,018	R$	0,000720	R$	0,000560
Losartana	50	0,036	0,05	R$	0,001000	R$	0,000720
Nifedipina	20	0,036994558	0,042341086	R$	0,002117	R$	0,001850
Propronolol	40	0,012	0,015	R$	0,000375	R$	0,000300
Isossorbida	10	0,057	0,175	R$	0,017500	R$	0,005700

Legenda Quadro 5: medicamentos disponíveis na REMUME e utilizados pelos pacientes deste estudo durante os anos de 2006 a 2012.

O transporte teve como base de cálculo o valor da tarifa única em 2015 cobrada no município para o transporte público, equivalente a R$ 3,40. O salário médio dos pacientes que trabalhavam foi de R$ 2.252,02. A média do rendimento bruto ponderado pela taxa de INSS para contribuintes autônomos e não autônomos, 17,36% dos contribuintes, segundo o IBGE (BRASIL, 2008) foi de R$ 3.391,63. Foi considerada a alíquota de encargos descontados na fonte de pagamento como 26,8% para autônomos e 35,8% para não autônomos devido a variação da contribuição do INSS (Quadro 6). Ressalta-se que esse rendimento bruto é

sensível aos valores de rendimento médio real para o estado de São Paulo (BRASIL, 2015i). Utilizando o mesmo valor de rendimento bruto foi realizado o cálculo do custo do absenteísmo e do transporte (Quadro 7).

Quadro 6- Carga tributária descontada do salário do trabalhador no Brasil

Impostos	Taxa (%)	Alíquota
INSS	11,0 ou 20,0	0,11 ou 0,20
Seguro Acidente de Trabalho	2,0	0,02
Salário Educação	2,5	0,025
Incra	0,2	0,002
Sesi/Sesc/Sest	1,5	0,015
Senai/Sanac/Senat	1,0	0,01
Sebrae	0,6	0,006
**Encargos sociais**	**18,8**	**0,188**
FGTS	8,0	0,08
**TOTAL**	**26,8 ou 35,8**	**0,268 ou 0,358**

FONTE: Adaptado do Ministério do Trabalho e Emprego (BRASIL, 2006).

Quadro 7- Determinação dos custos diretos não médicos

Transporte		Absenteismo	
Tarifa (ida e volta)	R$ 7,80	Rendimento Bruto anual	R$ 44.091,19
		Hora trabalhada anual	1936h
		Valor da hora trabalhada	R$ 22,77
Pacientes trabalhadores autônomos ou idosos ou não trabalhadores	86%	Custo quanto ao periodo de consulta (4h)	R$ 91,09
		Pacientes trabalhadores	28%
**CUSTO**	**R$ 5,84**	**CUSTO**	**R$ 25,32**

Legenda Quadro 7: o custo do absenteísmo é o valor do custo da consulta multiplicado pela taxa de pacientes trabalhadores; O custo do transporte é o custo da tarifa ida e volta multiplicado pela taxa de pacientes que eram trabalhadores autônomos ou idosos ou não trabalhadores.

5.3.1. Análise direta dos custos discriminados e total

A modificação nas medidas das variáveis clínicas e assistenciais em associação com a ATF representaram alterações no custo do tratamento dos pacientes hipertensos acompanhados. A descrição dos custos representa o custo do tratamento ofertado pelo SUS aos 104 pacientes hipertensos. Destaca-se que houve a redução nos custos com as consultas de

pronto atendimento e especialidade e com o transporte para os períodos ATF e pós ATF. No período ATF é possível notar que o custo com absenteísmo foi menor. De forma geral, o custo total da assistência à saúde do paciente hipertenso foi menor no período ATF (Tabela 5 e Tabela 6).

Tabela 5 – Custos discriminados por ano e por período para a análise direta de custos

Custos discriminados	2006	2007	2008	2009	2010	2011	2012
		**Pré ATF**		**ATF**		**Pós ATF**	
CUSTOS DIRETOS MÉDICOS							
**Exames**	R$ 822,21	R$ 1.040,61	R$ 918,56	R$ 1.207,62	R$ 1.368,21	R$ 1.136,96	R$ 1.027,76
(média)		**R$ 927,13**		**R$ 1.207,62**		**R$ 1.177,65**	
**Consultas**							
Atendimento Básico	R$ 20.798,04	R$ 17.115,06	R$ 18.198,29	R$ 21.664,63	R$ 23.181,15	R$ 28.813,95	R$ 30.330,48
Atendimento Especializado	R$ 6.318,45	R$ 6.318,45	R$ 5.856,12	R$ 6.010,23	R$ 4.469,15	R$ 6.318,45	R$ 4.931,47
Pronto Atendimento	R$ 10.282,28	R$ 11.790,35	R$ 16.862,95	R$ 10.008,09	R$ 11.790,35	R$ 7.814,54	R$ 5.346,79
(média Atendimento Básico)		**R$ 18.703,80**		**R$ 21.664,63**		**R$ 27.441,86**	
(média Atendimento Especializado)		**R$ 6.164,34**		**R$ 6.010,23**		**R$ 5.239,69**	
(média Pronto Atendimento)		**R$ 12.978,53**		**R$ 10.008,09**		**R$ 8.317,23**	
**Medicamentos AH**	R$ 3.029,94	R$ 3.554,90	R$ 3.556,92	R$ 4.071,95	R$ 3.415,93	R$ 3.567,22	R$ 3.624,84
(média)		**R$ 3.380,59**		**R$ 4.071,95**		**R$ 3.536,00**	
CUSTOS DIRETOS NÃO MÉDICOS							
**Transporte**	R$ 227,58	R$ 345,04	R$ 403,76	R$ 278,96	R$ 300,99	R$ 315,67	R$ 242,26
(média)		**R$ 325,46**		**R$ 278,96**		**R$ 286,31**	
**Absenteísmo**	R$ 4.686,61	R$ 5.673,26	R$ 7.646,57	R$ 4.933,27	R$ 6.413,25	R$ 6.906,58	R$ 6.289,92
(média)		**R$ 6.002,15**		**R$ 4.933,27**		**R$ 6.536,58**	

Legenda Tabela 5: ATF = Atenção Farmacêutica; AH = Anti-hipertensivo.

Tabela 6 – Custos totais para a análise direta dos custos

Custos	2006	2007	2008	2009	2010	2011	2012			
		**Pré ATF**		**ATF**		**Pós ATF**		Diferença 1	Diferença 2	Diferença 3
**Custo total**	R$ 46.165,11	R$ 45.837,66	R$ 53.443,17	R$ 48.174,75	R$ 50.939,03	R$ 54.873,38	R$ 51.793,52			
(média)		**R$ 48.481,98**		**R$ 48.174,75**		**R$ 52.535,31**		-R$ 307,23	R$ 4.053,33	R$ 4.360,56
**Custo/Paciente**	R$ 443,90	R$ 440,75	R$ 513,88	R$ 463,22	R$ 489,80	R$ 527,63	R$ 498,01			
(média)		**R$ 466,17**		**R$ 463,22**		**R$ 505,15**		-R$ 2,95	R$ 38,97	R$ 41,93

Legenda Tabela 6: ATF = Atenção Farmacêutica; AH = Antihipertensivo; Diferença 1= ATF- pré ATF; Diferença 2= pós ATF- pré ATF; Diferença 3= pós ATF-ATF.

A prevalência estimada de HAS para os indivíduos acima de 30 anos em Ribeirão Preto-SP é de 59%, segundo os dados da SMS-RP em 2015 (DIB, RIERA, FERRAZ, 2010). O município empregou em saúde no ano de 2013 26,16% dos R$ 1.180.945.729,29 arrecadados neste mesmo ano, o que representou a média de custo com a assistência à saúde de R$ 639,17 habitante / ano para a população de 649 mil habitantes do município (BRASIL, 2012b; BRASIL, 2015l; BRASIL 2015m). O custo empenhado, anualmente, para o tratamento do paciente hipertenso no estado de São Paulo, ajustado para o ano de 2015, foi de R$ 409,46, o que representa 64,06% dos gastos em saúde por habitante / ano em Ribeirão Preto (PONTES-NETO et al., 2008; DIB, RIERA, FERRAZ, 2010; BRASIL, 2015o).

Ao relacionar a estimativa da prevalência de HAS na população do município com idade acima de 29 anos, 205.546 mil habitantes, com o custo do paciente hipertenso para o estado de São Paulo, é possível mensurar um gasto de R$ 84.163.646,38 anuais com a hipertensão no município, sem incorporar nestes gastos as comorbidades ocorridas pelas complicações da doença. Esse valor remete a 19,91% dos gastos em saúde do município que foi de R$ 422.736.794,38, no ano de 2013, considerando os valores dos recursos totais aplicados da esfera federal, estadual e municipal. Utilizando como base o valor empenhado apenas pelo município de R$ 309.049.332,13, esse percentual passou para 27,23% dos gastos em saúde (BRASIL, 2012b; BRASIL, 2015l; BRASIL 2015m).

O período pré ATF remete aos anos que o tratamento convencional foi ofertado aos pacientes hipertensos do estudo pelo sistema de saúde, o *baseline*. Neste período o custo anual por paciente diagnosticado com HAS foi de R$ 466,17 para o município, R$ 56,71 a mais que a média empenhada no estado de São Paulo. O que representa um custo de R$ 96.109.049,14 com a população hipertensa no município, 31,10% dos gastos em saúde municipal.

Foi possível notar que a ATF reduziu o custo do tratamento convencional ofertado pelo SUS para R$ 463,20, o que representa uma economia anual de R$ 898.441,12 para o município. Diante disso, foi observada a redução de R$ 154,11 e R$ 924,65 no custo com consultas na cardiologia e R$ 2.970,44 e R$ 4.662,30 com consultas de pronto atendimento, respectivamente para os períodos ATF e pós ATF. Quanto às consultas do atendimento básico houve o aumento de R$ 2.960,83 e R$ 8.738,06 nos custos, respectivamente, para ambos os períodos. Essa variação de custos pode ser explicada pelo aumento na média do número de consultas no atendimento básico, que foi o segmento com consulta de maior custo R$ 106,22, e a redução no número de consultas de emergência, segmento com consulta de menor custo R$ 67,21. O que repercutiu no aumento do custo por paciente hipertenso pós ATF, correspondendo a R$ 505,15.

Contudo, é possível relacionar esse incremento dos custos após alta do paciente à promoção de uma assistência à saúde mais adequada ao cuidado do hipertenso, já que houve evidência para a associação da redução das crises hipertensivas em decorrência da melhora nos níveis pressóricos alcançados pela ATF. Adicionalmente, a ATF promoveu o cuidado do paciente no nível primário de atenção à saúde. Neste caso, é importante enfatizar que essa modificação foi fundamental para reduzir custos futuros com a HAS e promover a qualidade de vida para o paciente, uma vez que, as doenças crônicas não transmissíveis, como a HAS, a

reeducação para um cuidado diário e preventivo é fundamental, sendo viável que essa estratégia ocorra no nível de atendimento básico (SBC, 2010). Isso reforça a importância da ATF para a promoção da saúde e o empoderamento do paciente para o cuidado da sua saúde como estratégias capazes de evitar custos futuros com comorbidades ocasionadas pela HAS (ARAÚJO, UETA, FREITAS, 2005; BORGES, 2008).

Devido o custo direto não médico estar associado às consultas, as modificações no número das consultas inferiram na redução de R$ 46,50 e de R$ 39,15 nos custos de transporte, em ambos os períodos, e na redução dos custos com o absenteísmo, apenas para o período ATF. Para os custos da assistência a saúde quanto aos exames laboratoriais e medicamentos AH houve o aumento em ambos os períodos. O custo com os medicamentos por paciente / ano foi de R$ 26,98, R$ 31,88 e R$ 29,96, respectivamente nos períodos pré ATF, ATF e pós ATF. Ao mensurar o custo com medicamentos AH no período ATF e pós ATF por habitante do município, R$ 14,26 e R$ 13,39, respectivamente, verifica-se que não foi ultrapassada a média do custo com medicamentos empenhado pelo município por habitante, R$ 16,02 e nem mesmo o valor gasto por habitante/ano com a Assistência Farmacêutica básica no município em 2012, R$ 18,73, valor ajustado para 2015 (BRASIL, 2012b; BRASIL, 2015l; BRASIL 2015m).

Acrescentando os custos do investimento na ATF foi possível notar que o custo total do tratamento dos pacientes hipertensos aumentou para R$ 99.693,35, o que representa um custo de R$ 958,59 por paciente. Apesar de esse valor ser maior que o decréscimo de custos ocasionado pela ATF no tratamento convencional ofertado pelo SUS, ele representa uma importante resposta no resultado clínico a curto prazo, uma vez que, houve evidência da associação do programa de ATF com a melhora da PAS, PAD e CT, mostrando que esses resultados clínicos foram mantidos mesmo após a alta dos pacientes, período em que não houve mais gastos com a ATF. Adicionalmente, houve evidência da ATF estar associada à redução do risco cardiovascular em dez anos, sendo este resultado também mantido três anos após a alta.

5.3.2. Análise da razão custo efetividade e razão de custo efetividade incremental para os custos diretos

O percentual de níveis pressóricos satisfatórios foi considerado como desfecho para as análises de custo efetividade. É notável que a média desse percentual por período difira da

média realizada para cada ano, pois por período o número de pacientes com dados ausentes diminuiu, o que aumentou o *n* para o cálculo da média por período e tornou mais representativo à coorte de pacientes deste estudo.

Analisando a RCE é possível observar que o custo para o sistema de saúde controlar a pressão arterial de um paciente foi maior com a ATF, o que ocorreu devido ao investimento necessário nesta TS, porém após a ATF, nota-se que o custo para controlar a pressão arterial de um paciente reduziu no período e em todos os anos. O custo no período pós ATF diminuiu de R$ 313,77 anuais por paciente com a pressão arterial controlada. Ao analisar a RCEI, verifica-se que o custo para ter um paciente a mais no ano com a pressão arterial controlada foi R$ 1.124,24 com a ATF e R$ 100,98 após a alta. Ressalta-se que nos anos 2010 e 2012 do período pós ATF comparado ao último ano antes dos pacientes serem acompanhados pela ATF (2008) a RCE foi negativa, revelando que nestes anos a ATF mostrou ser uma TS menos onerosa e com melhores desfechos (Tabela 7).

Tabela 7 – Análise da razão de custo efetividade e da razão de custo efetividade incremental por ano e período

	Pré ATF			ATF	Pós ATF		
	**2006**	**2007**	**2008**	**2009**	**2010**	**2011**	**2012**
Custo	R$ 443,90	R$ 440,75	R$ 513,88	R$ 958,59	R$ 489,80	R$ 527,63	R$ 498,01
Desfecho	56% controlado	60% controlado	64% controlado	98% controlado	98% controlado	88% controlado	88% controlado
**RCE**	R$ 792,67	R$ 734,58	R$ 802,93	R$ 978,15	R$ 499,79	R$ 599,58	R$ 565,93
**RCEI (2009)**	R$ 1.225,46	R$ 1.362,74	R$ 1.307,98				
**RCEI (2010)**	R$ 109,29	R$ 129,08	-R$ 70,82				
**RCEI (2011)**	R$ 261,67	R$ 310,29	R$ 57,30				
**RCEI (2012)**	R$ 169,12	R$ 204,53	-R$ 66,09				
Custo médio por período		R$ 466,17		R$ 958,59		R$ 505,15	
Desfecho por período		54,4% controlado		98,2% controlado		93,0% controlado	
**RCE**		R$ 856,94		R$ 976,16		R$ 543,17	
**RCEI (ATF)**		R$ 1.124,24					
**RCEI (Pós ATF)**		R$ 100,98					

Legenda Tabela 7: RCE = Razão média de custo-efetividade (RCE=custo/desfecho); RCEI = Razão de custo-efetividade incremental (RCEI=Δ custos/ Δ desfechos); Desfecho = Percentual de controle pressórico entre os pacientes.

Ao analisar os resultados no plano custo-efetividade, observa-se que o ano de 2010 houve maior efetividade no controle pressórico a um menor custo. Ressalta-se que o tratamento incluindo a ATF teve maior efetividade que o tratamento convencional em todas as comparações, destacando os anos após a alta da ATF, ou seja, 2010 e 2012 quando comparados ao ano de 2008, pois foram dominantes. Assim, além da ATF ser mais efetiva nesses anos, houve a redução no custo do tratamento (Figura 7). Quando observado por período, nota-se que no período pós ATF houve menor impacto desta TS nos desfechos, o que foi compensado pela menor diferença de custos (Figura 8).

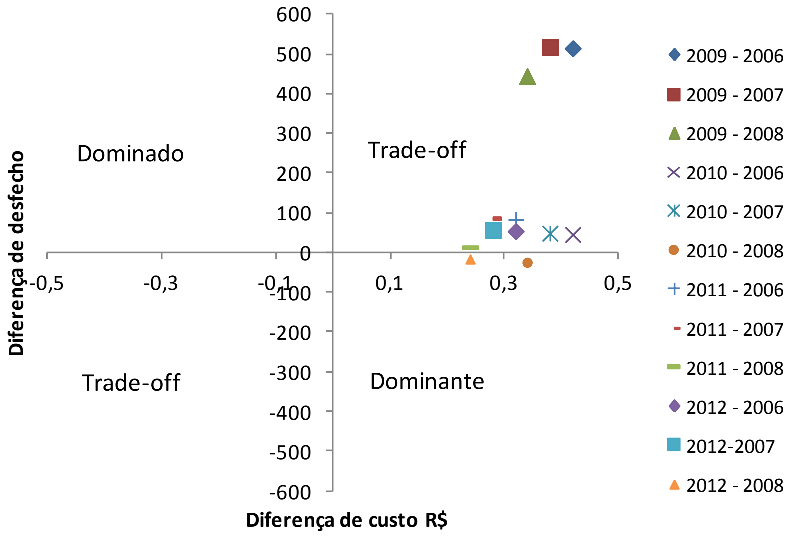

Figura 7 – Plano de custo-efetividade da Atenção Farmacêutica por anos.

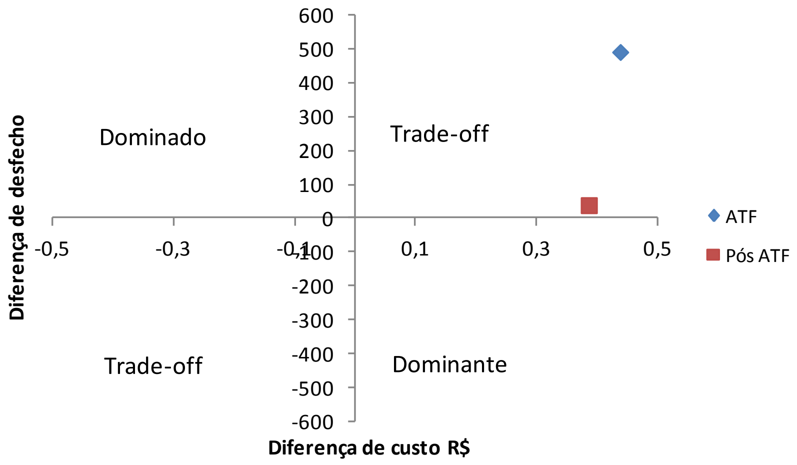

Figura 8 - Plano de custo-efetividade da Atenção Farmacêutica por período.

Traçando o limiar de custo efetividade com a linha de tendência do *baseline* do estudo (2006, 2007 e 2008), verifica-se que os anos após a intervenção (2010, 2011 e 2012), estão situados abaixo do limiar, o que significa que a ATF foi uma intervenção custo efetiva se comparada ao tratamento convencional ofertado ao paciente hipertenso no SUS. Destaca-se o ano de 2010, por ter o menor custo para o sistema de saúde para controlar a pressão arterial de um paciente (Figura 9).

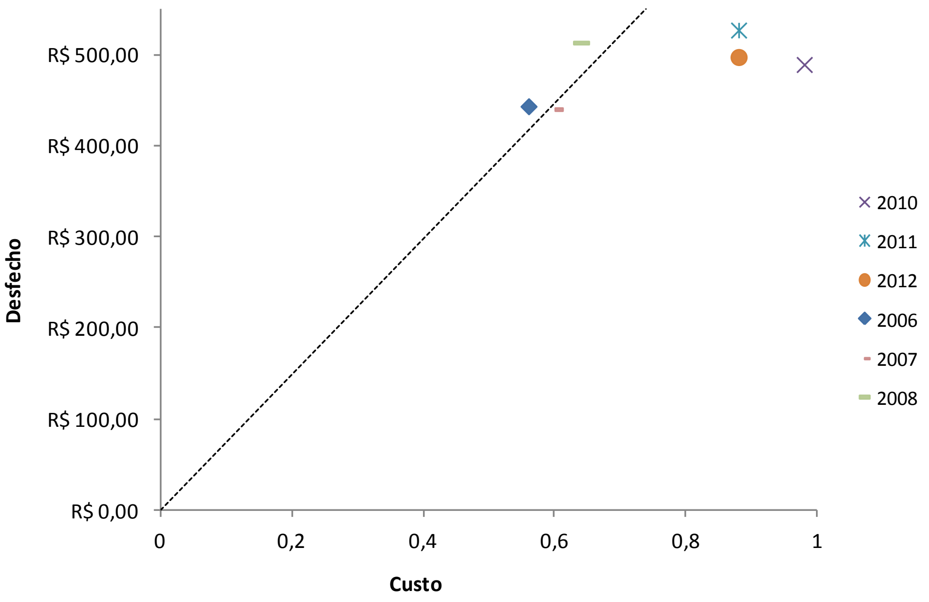

Figura 9 – Limiar de custo-efetividade baseado na linha de tendência nos anos pré ATF.

5.3.3. Análise de sensibilidade pelo benefício líquido incremental

Atribuindo o valor de um real como valor mínimo da disposição a pagar para controlar a pressão de um paciente no ano, se obtém um benefício líquido incremental (BLI) negativo, equivalente a -R$ 491,41. Como valor máximo, é recomendável pela REBRATS utilizar três vezes o valor do PIB per capita (R$ 81.687,00) como limiar de custo-efetividade (BRASIL, 2015p). Utilizando esse valor como a disposição a pagar pelo controle pressórico de um paciente durante o ano, foi obtido um BLI de R$ 35.286,49. Dessa forma, os valores de –R$ 491,41 a R$ 35.286,49 representaram um intervalo que indica que os resultados são sensíveis para atribuir um valor à disposição a pagar pelo controle pressórico de um paciente / ano. Neste caso, a disposição a pagar de R$ 1.124,24 pelo controle pressórico de um paciente durante um ano, que é a $RCEI_{ATF}$, representa o valor para o BLI começar a ser positivo (Figura 10). Após a ATF o intervalo de sensibilidade diminuiu, –R$ 38,58 e R$ 31.492,21, e o BLI começou a ser positivo para a disposição a pagar de R$ 100,98 pelo controle pressórico de um paciente durante um ano. Quando colocado como disposição a pagar no período pós ATF o valor de R$ 492,41 empenhados a mais para cada paciente assistido pela ATF em 2009 obtém-se um BLI de R$ 151,01. O BLI positivo significa, nesta análise, que ao desembolsar o custo da ATF como disposição a pagar, essa TS ainda é custo efetiva mesmo para o período no qual não há mais a intervenção aos pacientes (Figura 11).

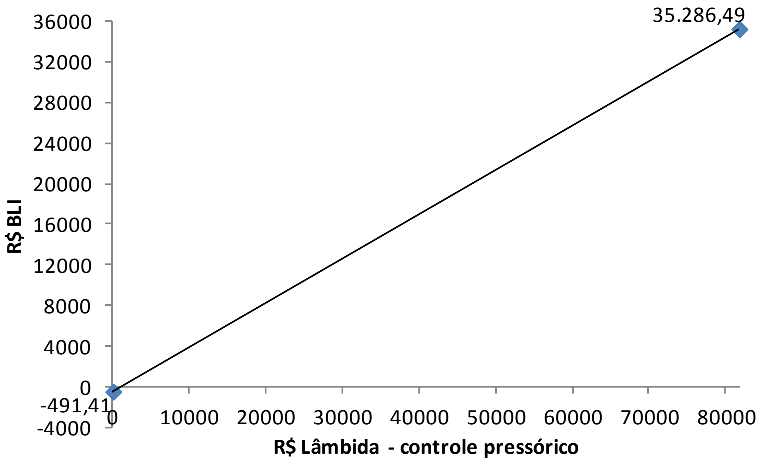

Figura 10 – Análise da sensibilidade pelo benefício líquido incremental para a Atenção Farmacêutica.

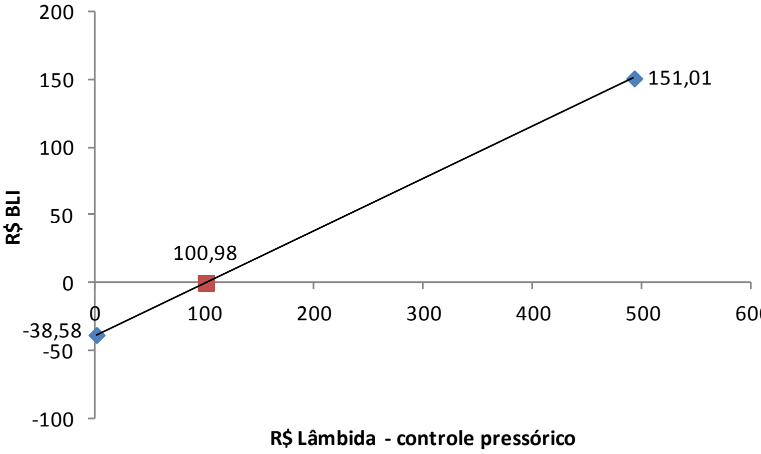

Figura 11 – Análise da sensibilidade pela disposição a pagar ao desfecho de controle pressórico que a Atenção Farmacêutica alcançou após a alta dos pacientes.

De acordo com Etemad & Hay (2003) o cuidado farmacêutico em um programa de dispensação de medicamentos foi custo-efetivo, sendo capaz de reduzir custos com complicações. Em adição a sensibilidade da análise de custo-efetividade desse programa foi de 14 - 45% do máximo estabelecido como limiar. Este estudo mostrou a sensibilidade para o programa de ATF de 0,42% do limiar de custo-efetividade estabelecido pelo BLI após a alta dos pacientes. O que evidencia a ATF como uma TS capaz de ser incorporada ao SUS para melhorar o controle pressórico.

5.4. Determinação dos custos das complicações da hipertensão e dos custos indiretos

Ao custo com a hospitalização foi acrescido o custo do SAMU, pois neste estudo foi considerado que para todo agravo de urgência o SUS terá disponível este serviço. Nessa

categoria de custos, observa-se que o maior custo foi para a DIC, equivalente a R$ 8.937,14. O custo pós-evento de maior impacto foi para DRC, R$ 27.928,68, custo esse atribuído ao valor da diálise, realizando treze sessões por mês a um custo médio de R$ 179,03 / paciente / sessão. Considerando as taxas estimadas de invalidez para cada morbidade, 70% AVE, 60% DIC, 20% DAP, 57% IC e 57% DRC, a morbidade com maior custo para pensão por invalidez e contribuição de encargos foi o AVE, R$ 12.237,37 e R$ 3.271,18, respectivamente (Tabela 8).

Tabela 8 – Custos anuais das complicações da hipertensão e os custos indiretos

	Custo SAMU média anual/ocorrência	Custo hospitalização media anual/paciente	Custo Hospitalização + SAMU	Custo pós evento anual	Custo da pensão por invalidez com o adicional de 25%	Custo por morte (pensão por morte)	Contribuição de encargos	Contribuição de encargos (considerando o índice de invalidez)
**AVE**	R$ 230,56	R$ 3.361,31	R$ 3.591,86	R$ 3.350,10	R$ 12.237,37	R$ 12.881,44	R$ 4.673,11	R$ 3.271,18
**DIC**	R$ 230,56	R$ 8.706,58	R$ 8.937,14	R$ 4.282,07	R$ 10.949,22	R$ 12.881,44	R$ 4.673,11	R$ 2.803,87
**DAP**	R$ 230,56	R$ 3.031,95	R$ 3.262,51	R$ 2.365,00	R$ 5.796,65	R$ 12.881,44	R$ 4.673,11	R$ 934,62
**IC**	R$ 230,56	R$ 6.991,89	R$ 7.222,44	R$ 3.365,00	R$ 10.562,78	R$ 12.881,44	R$ 4.673,11	R$ 2.663,67
**DRC**	R$ 230,56	R$ 2.572,89	R$ 2.803,45	R$ 27.928,68	R$ 10.433,97	R$ 12.881,44	R$ 4.673,11	R$ 2.616,94

Legenda da Tabela 8: AVE = acidente vascular encefálico; DIC = doença isquêmica do coração; DAP = doença arterial periférica; IC = insuficiência cardíaca; DRC = doença renal crônica; SAMU = Serviço Ambulatorial Móvel de Urgência.

Os custos calculados na Tabela 8 foram a base para: cálculo dos custos de incidência + tratamento, custos com o tratamento apenas, custo da incidência + morte, e custo da morte relacionados às complicações da HAS. Dentre as complicações é possível notar que a DRC é a mais onerosa, custo de R$ 43.783,04 no ano em que há a incidência e de R$ 40.979,59 nos anos de tratamento. Todavia, quando há incidência seguida de morte a DIC é a mais onerosa, R$ 26.491,69, o que ocorre, provavelmente, devido aos custos com a incidência da DIC serem maiores que os custos com a DRC, e o custo do tratamento para DRC ser maior que o custo para o tratamento da DIC (Tabela 9).

Tabela 9 – Tabela dos custos das complicações da hipertensão quanto a aplicação na projeção de Markov

	Incidência e tratamento	Tratamento	Incidência e morte	Morte
**AVE**	R$ 22.450,51	R$ 18.858,65	R$ 21.146,41	R$ 17.554,55
**DIC**	R$ 26.972,30	R$ 18.035,16	R$ 26.491,69	R$ 17.554,55
**DAP**	R$ 12.358,78	R$ 9.096,27	R$ 20.817,06	R$ 17.554,55
**IC**	R$ 23.813,89	R$ 16.591,45	R$ 24.776,99	R$ 17.554,55
**DRC**	R$ 43.783,04	R$ 40.979,59	R$ 20.358,00	R$ 17.554,55

Legenda da Tabela 9: AVE = acidente vascular encefálico; DIC = doença isquêmica do coração; DAP = doença arterial periférica; IC = insuficiência cardíaca; DRC = doença renal crônica.

5.5. Determinação das probabilidades para a modelagem de Markov

As taxas e probabilidades de incidência calculadas para o modelo das complicações do AVE, DIC e DAP foram respectivas ao escore calculado pela escala *ASCVD* (Tabela 10). As probabilidades de prevalência foram proporcionais à prevalência de cada morbidade, dados da literatura. A probabilidade de morte e reincidência foi proveniente das taxas da literatura para cada morbidade. É notado que devido à redução do risco cardiovascular associada ao programa de ATF a probabilidade de incidência das DCVs foi menor no período pós ATF. A doença de maior probabilidade de letalidade ao incidir, foi a DIC e a de maior reincidência a DAP (Tabela 11).

Tabela 10 – Taxas e probabilidades calculadas para a incidência / 100 de acidente vascular encefálico, doença isquêmica do coração e doença arterial periférica

	Pre ATF	ATF
**Incidência DCV em 10 anos** (ASCVD)	19,50%	11,80%
**Taxa**	0,021690	0,012560
**Probabilidade**	0,021450	0,012480

Legenda da Tabela 10: DCV = Doença cardiovascular; *ASCVD* = escala de risco cardiovascular; ATF = Atenção Farmacêutica.

Tabela 11 – Taxas e probabilidades calculadas para a prevalência, morte e reincidência / 100 de acidente vascular encefálico, doença isquêmica do coração e doença arterial periférica

	Prevalência	Morte	Reincidência
**AVE**			
taxa	0,320000	0,324300	0,031000
Probabilidade	0,421000	0,276900	0,030524
**DIC**			
taxa	0,300000	0,586100	0,026800
Probabilidade	0,395000	0,443500	0,026541
**DAP**			
taxa	0,140000	0,300000	0,110000
Probabilidade	0,184000	0,029550	0,104165

Legenda da Tabela 11: AVE = acidente vascular encefálico; DIC = doença isquêmica do coração; DAP = doença arterial.

As taxas e probabilidades de incidência para as morbidades IC e DRC subdividiram-se no período pré ATF e ATF, por serem atribuídas, pela literatura, aos indivíduos com o controle insatisfatório da HAS. Dessa forma, foram calculadas com base na proporção de indivíduos com a PA em nível satisfatório. As probabilidades de morte foram provenientes das taxas da literatura para cada morbidade. Verifica-se que devido ao controle pressórico

associado à assistência com a ATF as probabilidades de incidência pós ATF foram menores (Tabela 12).

Tabela 12 – Taxas e probabilidades calculadas para a incidência / 100 e para a nº óbitos / 100 relativas à insuficiência cardíaca e doença renal crônica

Complicação da HAS	Incidência	Morte
**IC (Pre ATF)**		
taxa	0,010450	0,058800
Probabilidade	0,010396	0,057105
**IC (ATF)**		
taxa	0,000867	0,058800
Probabilidade	0,000867	0,057105
**DRC (Pre ATF)**		
taxa	0,015700	0,217970
Probabilidade	0,015577	0,195850
**DRC (ATF)**		
taxa	0,000971	0,217970
Probabilidade	0,000971	0,195850

Legenda da Tabela 12: IC = insuficiência cardíaca; DRC = doença renal crônica; ATF = Atenção Farmacêutica; morte = nº de óbitos/100.

5.6. Estruturação do modelo de Markov para a projeção das complicações da hipertensão

No modelo de Markov para a projeção das complicações AVE, DIC e DAP, todos os pacientes iniciaram no estágio de HAS. De acordo com as probabilidades poderiam continuar estáveis com HAS ou apresentar uma das complicações. Os pacientes que permaneciam estáveis voltavam para o estado de HAS no ciclo seguinte, aqueles que apresentaram algumas das complicações da HAS foram alocados no estado de saúde da incidência da respectiva morbidade. Neste estado o paciente teve a probabilidade de morrer ou ir para o estado de prevenção secundária da doença ou tratamento da DCV, no próximo ciclo. No ciclo seguinte, aqueles que estavam na prevenção secundária tiveram a probabilidade de reincidência de algumas das morbidades ou então de permanecer estável no tratamento da complicação (Figura 12).

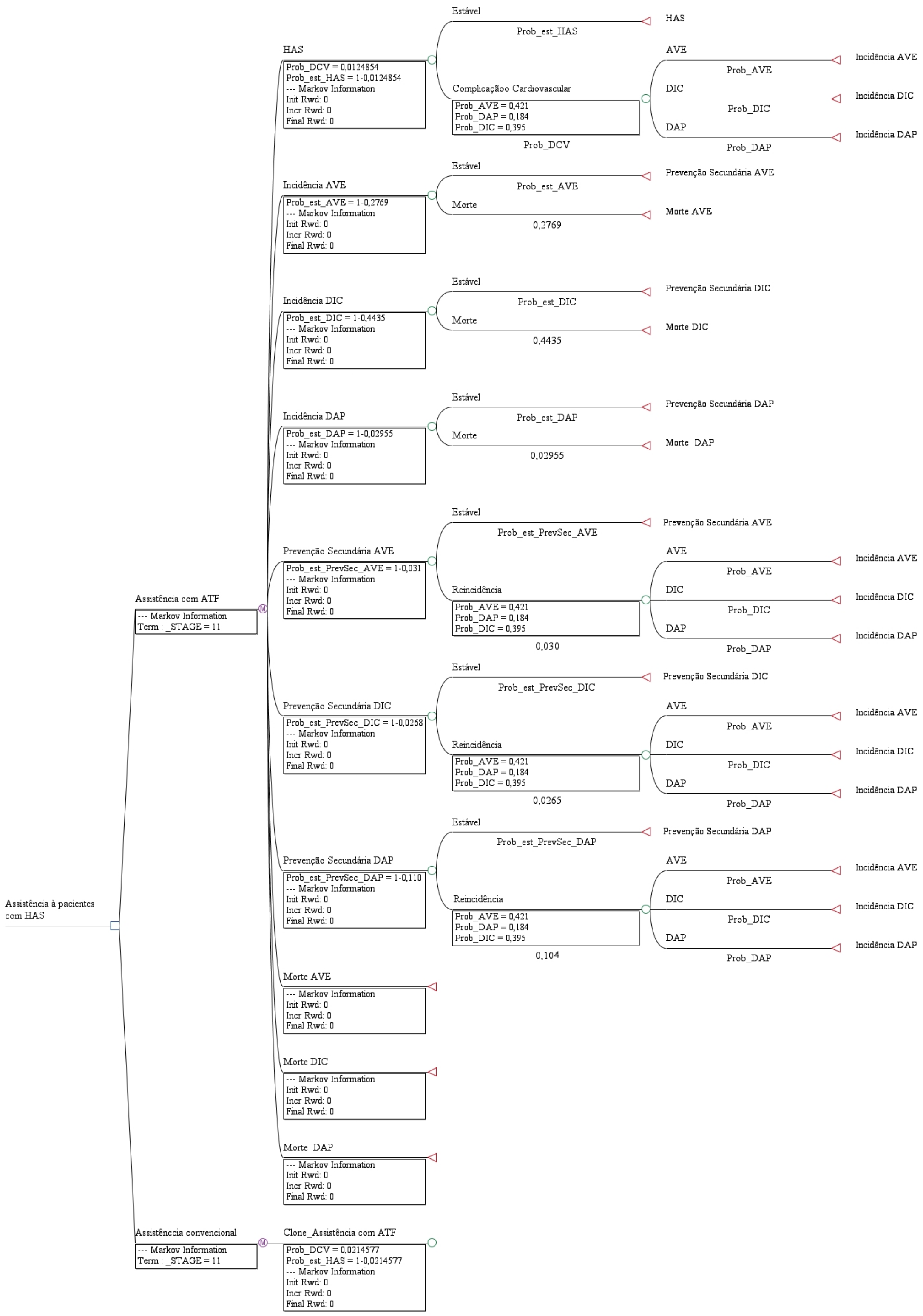

Legenda da Figura 12: ATF = Atenção Farmacêutica; Assistência convencional = assistência à saúde pelo SUS sem a ATF, dados relativos ao período pré ATF; Assistência com ATF = assistência à saúde com o programa de ATF, dados relativos aos períodos ATF e pós ATF; HAS = Hipertensão Arterial Sistêmica; AVE = Acidente Vascular Encefálico; DIC = Doença Isquêmica do Coração. O quadrado azul dividindo os primeiros dois ramos representa o nó de decisão para modelar a projeção com os dados referentes à assistência convencional do SUS e a com a ATF. O círculo roxo representa o nó de Markov do qual originam os ramos dos estados de saúde de Markov. Os círculos verdes significam nós de probabilidades e os triângulos no final da ramificação representa o nó terminal.

Figura 12 – Árvore de decisão para a aplicação da modelagem de Markov no modelo das doenças cardiovasculares, acidente vascular encefálico, doença isquêmica do coração e doença arterial periférica.

No modelo de Markov para a IC, os pacientes iniciaram todos no estado de saúde HAS e desse estado eles tiveram a chance, de acordo com as probabilidades, de incidência de IC e de permanecer estável com HAS no ciclo seguinte. Aqueles que tiveram IC foram para o estado de tratamento no ciclo seguinte. Neste estado o paciente teve a probabilidade de ficar estável fazendo o tratamento ou então morrer (Figura 13). Para a DRC a estruturação do modelo foi semelhante à IC, porém o estado de saúde de tratamento foi modificado para diálise e o modelo seguiu as probabilidades da DRC (Figura 14).

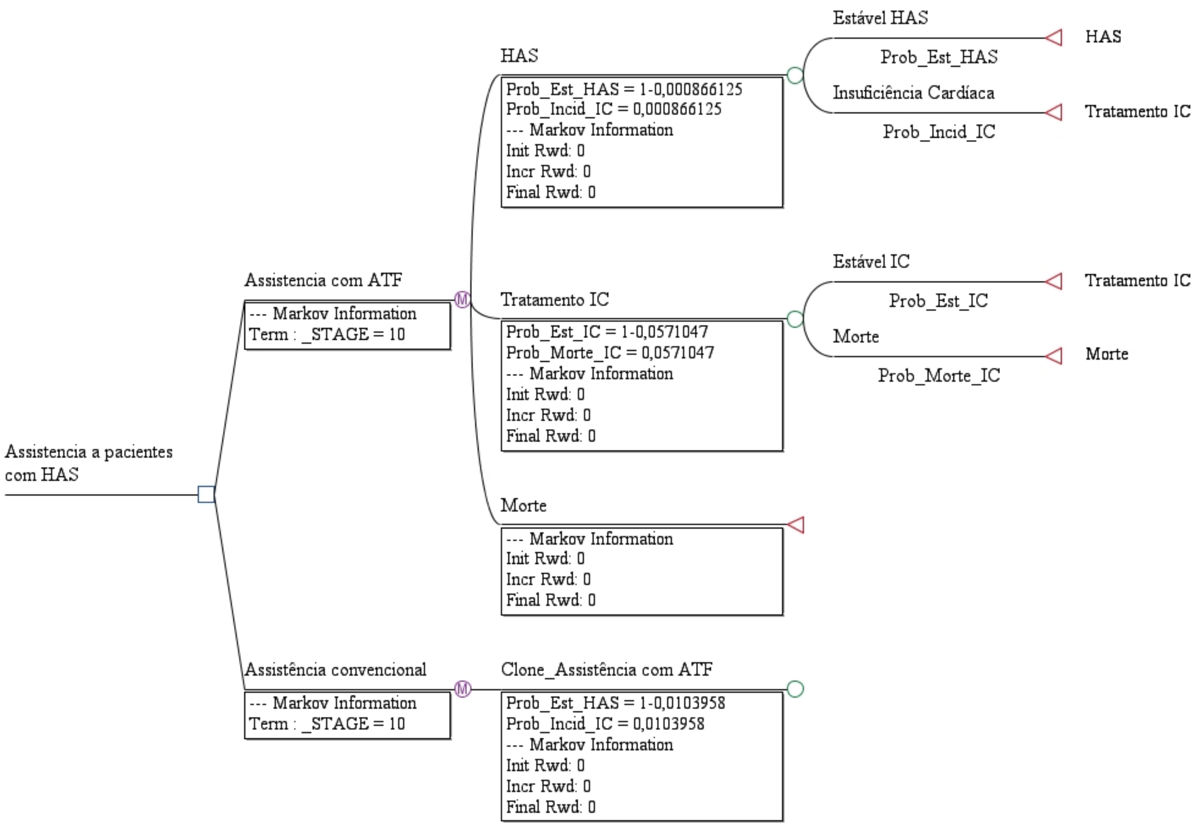

Legenda Figura 13: ATF = Atenção Farmacêutica; Assistência convencional = assistência à saúde pelo SUS sem a ATF, dados relativos ao período pré ATF; Assistência com ATF = assistência à saúde com o programa de ATF, dados relativos aos períodos ATF e pós ATF; HAS = Hipertensão Arterial Sistêmica; IC = Insuficiência Cardíaca. O quadrado azul dividindo os primeiros dois ramos representa o nó de decisão para modelar a projeção com os dados referentes à assistência convencional do SUS e com a ATF. O círculo roxo representa o nó de Markov do qual originam os ramos dos estados de saúde de Markov. Os círculos verdes significam nós de probabilidades e os triângulos no final da ramificação representa o nó terminal.

Figura 13 – Árvore de decisão para a aplicação da modelagem de Markov no modelo da insuficiência cardíaca crônica.

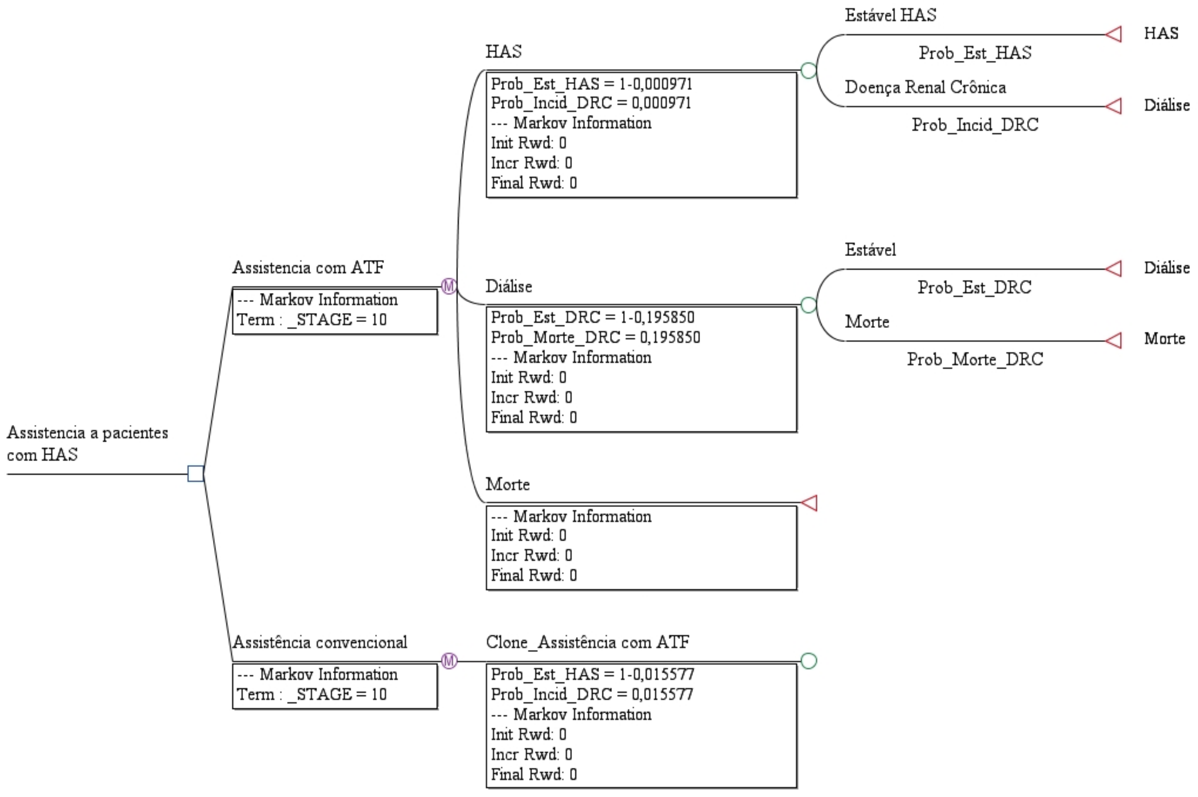

Legenda Figura 14: ATF = Atenção Farmacêutica; Assistência convencional = assistência à saúde pelo SUS sem a ATF, dados relativos ao período pré ATF; Assistência com ATF = assistência à saúde com o programa de ATF, dados relativos aos períodos ATF e pós ATF; HAS = Hipertensão Arterial Sistêmica; DRC = Doença Renal Crônica. O quadrado azul dividindo os primeiros dois ramos representa o nó de decisão para modelar a projeção com os dados referentes à assistência convencional do SUS e com a ATF. O círculo roxo representa o nó de Markov do qual originam os ramos dos estados de saúde de Markov. Os círculos verdes significam nós de probabilidades e os triângulos no final da ramificação representa o nó terminal.

Figura 14 – Árvore de decisão para a aplicação da modelagem de Markov no modelo da doença renal crônica.

A projeção a cada ano para as complicações da HAS foi realizada para a assistência convencional à saúde (período pré ATF) e para a assistência à saúde com o programa de ATF (período ATF e pós ATF). Dessa forma, os pacientes foram alocados em cada estado de saúde para ambos os tipos de assistência em dez anos (Tabela 13).

Tabela 13 – Tabela da projeção dos estados de saúde de Markov, apresentando os anos de vida ganhos, anos de vida ajustados pela qualidade e o número necessário para tratar

Condições de saúde	Ano 1		Ano 2		Ano 3		Ano 4		Ano 5		Ano 6		Ano 7		Ano 8		Ano 9		Ano 10	
	Sem ATF	Com ATF	Sem ATF	Com ATF	Sem ATF	Com ATF	Sem ATF	Com ATF	Sem ATF	Com ATF	Sem ATF	Com ATF	Sem ATF	Com ATF	Sem ATF	Com ATF	Sem ATF	Com ATF	Sem ATF	Com ATF
HAS	100	104	94	102	90	100	84	98	80	98	74	97	71	94	67	94	62	93	58	89
AVE																				
Tratamento	1	0	1	1	2	1	3	2	3	2	4	2	4	3	5	3	6	3	6	4
Morte	0	0	1	0	1	0	1	1	1	1	2	1	2	1	2	1	2	1	3	2
DIC																				
Tratamento	0	0	1	1	1	1	2	1	2	1	3	2	3	2	4	2	4	2	5	3
Morte	0	0	1	0	1	1	2	1	2	1	2	1	3	2	3	2	4	2	4	2
DAP																				
Tratamento	0	0	1	0	1	1	1	1	2	1	2	1	2	1	2	1	3	2	3	2
Morte	0	0	0	0	0	0	0	0	0	0	0	0	0	0	0	0	0	0	0	0
IC																				
Tratamento	1	0	2	0	3	0	4	0	5	0	6	0	7	1	7	1	7	1	8	1
Morte	0	0	0	0	0	0	0	0	1	0	1	0	1	0	2	0	2	0	2	0
DRC																				
Tratamento	2	0	3	0	4	0	5	0	5	0	6	0	6	0	6	0	7	0	7	0
Morte	0	0	0	0	1	0	2	0	3	0	4	0	5	0	6	0	7	0	8	1
**Morte Total**	**0**	**0**	**2**	**0**	**3**	**1**	**5**	**2**	**7**	**2**	**9**	**2**	**11**	**3**	**13**	**3**	**15**	**3**	**17**	**5**
**AVAQ/ano**	0,66	0,85	0,64	0,84	0,63	0,83	0,61	0,82	0,60	0,82	0,58	0,81	0,56	0,80	0,55	0,80	0,53	0,80	0,51	0,78
**AVAQ (total)**	0,66	0,85	1,30	1,69	1,93	2,52	2,54	3,34	3,14	4,16	3,72	4,97	4,28	5,77	4,83	6,57	5,36	7,37	5,87	8,15
**Anos de vida**	1,00	1,00	1,97	2,00	2,95	2,99	3,90	3,97	4,83	4,95	5,75	5,93	6,64	6,90	7,52	7,88	8,37	8,85	9,21	9,80
**AVG**	0,00		0,02		0,04		0,07		0,12		0,18		0,26		0,36		0,48		0,59	
**NNT**	26 (25,0%)		13 (12,5%)		10 (10,0%)		7 (7,1%)		6 (5,5%)		5 (4,3%)		5 (4,3%)		4 (3,6%)		3 (3,1%)		3 (3,1%)	

Legenda Tabela 13: ATF = Atenção Farmacêutica; sem ATF = assistência à saúde pelo SUS sem a ATF, dados relativos ao período pré ATF; com ATF = assistência à saúde com o programa de ATF, dados relativos aos períodos ATF e pós ATF; HAS = hipertensão arterial sistêmica; AVE = acidente vascular encefálico; DIC = doença isquêmica do coração; IC = insuficiência cardíaca; DRC = doença renal crônica; AVAQ = anos de vida ajustados pela qualidade; AVG = anos de vida ganhos; NNT = número necessário para tratar.

Para o cálculo dos AVAQs foi considerado para a pontuação da condição de apenas hipertenso o valor de 0,668 para o período pré ATF e de 0,853 para o período pós ATF, valores mensurados pelo instrumento de qualidade de vida SF-36 como resultado deste programa de ATF. Para os pacientes com complicações da HAS a pontuação foi de 0,5, e no caso de morte teve a pontuação zero como qualidade de vida. Essa pontuação foi atribuída à proporção de paciente de cada condição de saúde. Os AVAQs e os anos de vida foram calculados conforme Rascati (2010), a diferença entre os anos de vida obtidos entre a assistência à saúde convencional e com o programa de ATF foi utilizada para expressar os AVGs.

Dos 104 pacientes do estudo, 46 apresentaram complicações da HAS, sendo 31 delas complicações cardiovasculares, e 17 pacientes morreram no total de dez anos quando assistidos pelo modelo assistencial convencional. No modelo assistencial com a ATF, 15 apresentaram complicações da HAS, sendo 14 delas complicações cardiovasculares, e 5 pacientes morreram ao longo de dez anos. Com a ATF houve menor incidência de complicações como IC e DRC, já que estão estritamente associadas ao controle pressórico. O

que é justificado, uma vez que, na assistência com a ATF 93% dos pacientes apresentaram a PA em níveis satisfatórios após a alta.

Considerando as condições da HAS e as suas complicações, nota-se pelo NNT que a cada três pacientes assistidos pelo programa de ATF uma complicação da HAS foi evitada em dez anos. Em adição, o modelo assistencial convencional proporcionou aos pacientes hipertensos, em dez anos, uma qualidade de vida menor quando comparada ao modelo com a ATF, respectivamente, 5,87 e 8,15 anos vividos com qualidade de vida em dez anos, o que representa 2,28 anos ganhos com qualidade. A mesma comparação pode ser feita aos anos de vida, sendo 9,21 anos de vida para a coorte dos pacientes assistidos pelo modelo convencional e 9,80 anos quando essa mesma coorte é assistido pelo modelo que incorpora a ATF, o que representa 0,59 anos ganhos.

Utilizando essas medidas como desfecho foi possível calcular a RCEI. O sistema de saúde gasta R$ 466,17 anuais para tratar o paciente hipertenso com a assistência convencional e R$ 958,59 anuais para tratar o paciente com o modelo de ATF incluso na assistência. Isso representa que ao incorporar a ATF para o tratamento dos pacientes hipertensos, houve o custo total de R$ 43.725,15 / AVAQ e R$ 168.971,78 / AVG; e por paciente hipertenso foi R$ 215,97 /AVAQ e R$ 869,51 / AVG. Adicionalmente, referindo esta análise aos custos pós ATF, onde não há mais investimento nesta TS, o custo total foi R$ 23.041,80 / AVAQ e 89.042,89 / AVG; o que representa para cada paciente R$ 17,09 / AVAQ e R$ 28,98 / AVG. O estudo de Grover et al. (2008) mostrou que o tratamento preventivo para HAS, recomendado pela diretriz canadense, é capaz de alcançar 0,2 AVGs, atrubindo a RCEI de R$ 144.385,00 por AVGs em comparação com o tratamento convencional. Ressalta-se que a ATF foi capaz de promover o cuidado preventivo dos pacientes hipertensos e adicionalmente melhorou a qualidade de vida dos pacientes deste estudo, reduzindo a ocorrência das complicações da HAS e o risco cardiovascular.

O custo de um ano vivido a mais e um ano vivido a mais com qualidade pode ser considerado intangível, mas ao atribuir o limiar de três vezes o PIB per capta, R$ 81.687, verifica-se que a ATF foi custo efetiva para AVAQ. Todavia, esses valores mostram que um custo de investimento na saúde capaz de reduzir complicações e reduzir custos futuros é capaz de melhorar a qualidade de vida e prolongar a vida de pacientes com hipertensão arterial, o que remete a um impacto positivo economicamente quando previsto à atividade econômica e ocupacional do indivíduo.

5.7. Projeção dos custos para dez anos

A projeção dos custos para a HAS, calculados para a morbidade na primeira etapa deste estudo farmacoeconômico, seguiu uma distribuição logarítmica natural. De acordo com a distribuição dos dados foi previsto que na ausência de inflação, estes custos tendem a aumentar com o tempo, de forma que a cada ano esse aumento diminui (Figura 15).

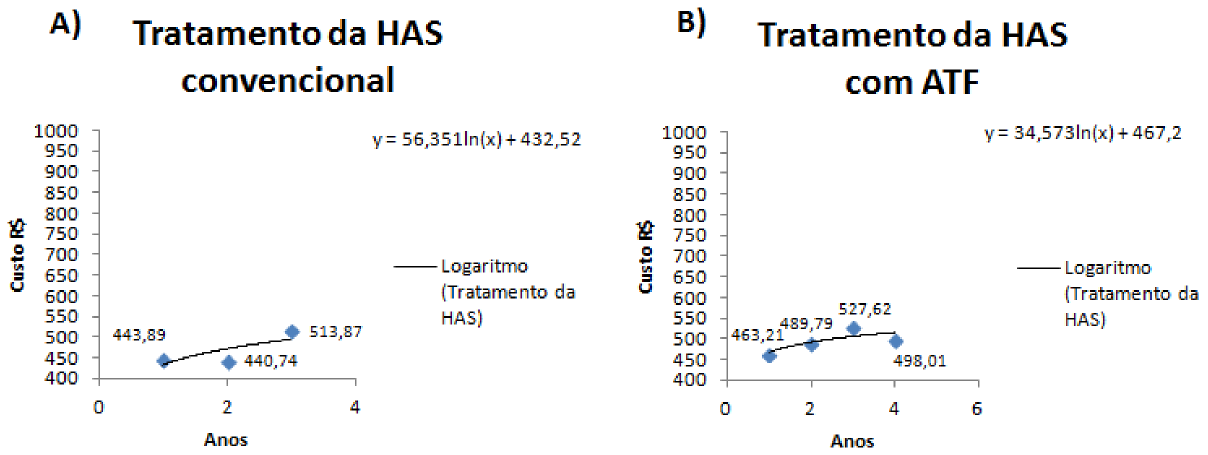

Legenda Figura 15: A figura **A)** representa a distribuição dos custos nos anos pré ATF e a figura **B)** representa a distribuição nos anos ATF e pós ATF. Na equação, o custo (y) é representado por uma equação Logarítmica Natural que varia com o tempo (x).

Figura 15 – Projeção logarítmica natural para aos custos da hipertensão calculados na primeira etapa do estudo.

Dessa forma, os custos foram projetados para o período de dez anos, considerando os custos no período da assistência convencional à saúde e os custos da assistência à saúde com o programa de ATF. Foi verificado, que a partir do ano quatro, os custos da assistência com a ATF por paciente hipertenso foi menor que o custo da assistência convencional (Tabela 14).

Tabela 14 – Custos diretos da hipertensão projetados em dez anos quanto ao tratamento convencional ofertado pelo Sistema Único de Saúde e ao tratamento com o programa de Atenção Farmacêutica

Tratamento convencional		Tratamento com ATF	
**Ano**	**Custo R$**	**Ano**	**Custo R$**
1	443,90	1	463,22
2	440,75	2	489,80
3	513,88	3	527,63
4	510,64	4	498,01
5	523,21	5	522,84
6	533,49	6	529,15
7	542,17	7	534,48
8	549,70	8	539,09
9	556,34	9	543,16
10	562,27	10	546,81

Os custos diretos do tratamento da HAS projetado a cada ano juntamente com os custos de incidência + tratamento, tratamento, incidência + morte e morte relativos a cada complicação da HAS somados aos custos indiretos, foram aplicados aos indivíduos a cada ano da projeção de Markov, o que ocorreu de acordo com a sua alocação nos estados de saúde. Verifica-se que com o passar do tempo ocorrem mais complicações da HAS quando não há assistência pelo programa de ATF, consequentemente o número de pacientes no estado de saúde HAS e o custo específico para o tratamento da HAS diminuem para a assistência sem ATF e o contrário acontece para a assistência com ATF. Contudo, os maiores custos estão associados às complicações da HAS, tanto que, um dos principais objetivos do tratamento desta morbidade é, justamente, evitar futuras complicações. Neste sentido, o modelo assistencial convencional esteve atrelado a maiores custos, justamente em decorrência dos gastos com as complicações da doença (Tabela 15).

Tabela 15 – Projeção dos custos diretos e indiretos para a hipertenção arterial sistêmica e as suas complicações no período de dez anos

Custo	Ano 1		Ano 2		Ano 3		Ano 4		Ano 5		Ano 6		Ano 7		Ano 8		Ano 9		Ano 10	
R$	Sem ATF	Com ATF	Sem ATF	Com ATF	Sem ATF	Com ATF	Sem ATF	Com ATF	Sem ATF	Com ATF	Sem ATF	Com ATF	Sem ATF	Com ATF	Sem ATF	Com ATF	Sem ATF	Com ATF	Sem ATF	Com ATF
**HAS**	44389,53	48174,75	41430,20	49959,44	46248,90	52762,87	42893,68	48805,44	41857,07	51238,62	39478,07	51327,21	38494,35	50240,74	36829,81	50674,70	34492,82	50514,31	32611,83	48665,85
**AVE**																				
Incid. e Trat	22.450,51	0,00	0,00	22.450,51	22.450,51	0,00	22.450,51	22.450,51	0,00	0,00	22.450,51	0,00	0,00	22.450,51	22.450,51	0,00	22.450,51	0,00	0,00	22.450,51
Tratamento	0,00	0,00	18.858,65	0,00	18.858,65	18.858,65	37.717,30	18.858,65	56.575,95	37.717,30	56.575,95	37.717,30	75.434,60	37.717,30	75.434,60	56.575,95	94.293,25	56.575,95	113.151,90	56.575,95
Incid e morte	0,00	0,00	21.146,41	0,00	0,00	0,00	0,00	21.146,60	0,00	0,00	21.146,60	0,00	0,00	0,00	0,00	0,00	0,00	0,00	21.146,60	21.146,60
morte	0,00	0,00	0,00	0,00	17.554,74	0,00	17.554,74	0,00	17.554,74	17.554,74	17.554,74	17.554,74	35.109,47	17.554,74	35.109,47	17.554,74	35.109,47	17.554,74	35.109,47	17.554,74
**DIC**																				
Incid. e Trat	0,00	0,00	26.972,30	26.972,30	0,00	0,00	26.972,30	0,00	0,00	0,00	26.972,30	26.972,30	0,00	0,00	26.972,30	0,00	0,00	0,00	26.972,30	26.972,30
Tratamento	0,00	0,00	0,00	0,00	18.035,16	18.035,16	18.035,16	18.035,16	36.070,32	18.035,16	36.070,32	18.035,16	54.105,48	36.070,32	54.105,48	36.070,32	72.140,64	36.070,32	72.140,64	36.070,32
Incid e morte	0,00	0,00	26.491,88	0,00	0,00	26.491,88	26.491,88	0,00	0,00	0,00	0,00	0,00	26.491,88	26.491,88	0,00	0,00	26.491,88	0,00	0,00	0,00
morte	0,00	0,00	0,00	0,00	17.554,74	0,00	17.554,74	17.554,74	35.109,47	17.554,74	35.109,47	17.554,74	35.109,47	17.554,74	52.664,21	35.109,47	52.664,21	35.109,47	70.218,95	35.109,47
**DAP**																				
Incid. e Trat	0,00	0,00	12.358,78	0,00	0,00	12.358,78	0,00	0,00	12.358,78	0,00	0,00	0,00	0,00	0,00	0,00	0,00	12.358,78	12.358,78	0,00	0,00
Tratamento	0,00	0,00	0,00	0,00	9.096,27	0,00	9.096,27	9.096,27	9.096,27	9.096,27	18.192,54	9.096,27	18.192,54	9.096,27	18.192,54	9.096,27	18.192,54	9.096,27	27.288,81	18.192,54
Incid e morte	0,00	0,00	0,00	0,00	0,00	0,00	0,00	0,00	0,00	0,00	0,00	0,00	0,00	0,00	0,00	0,00	0,00	0,00	0,00	0,00
morte	0,00	0,00	0,00	0,00	0,00	0,00	0,00	0,00	0,00	0,00	0,00	0,00	0,00	0,00	0,00	0,00	0,00	0,00	0,00	0,00
**IC**																				
Incid. e Trat	23.813,89	0,00	23.813,89	0,00	23.813,89	0,00	23.813,89	0,00	23.813,89	0,00	23.813,89	0,00	23.813,89	23.813,89	0,00	0,00	0,00	0,00	23.813,89	0,00
Tratamento	0,00	0,00	16.591,45	0,00	33.182,90	0,00	49.774,35	0,00	66.365,80	0,00	82.957,25	0,00	99.548,70	0,00	116.140,15	16.591,45	116.140,15	16.591,45	116.140,15	16.591,45
Incid e morte	0,00	0,00	0,00	0,00	0,00	0,00	0,00	0,00	24.777,18	0,00	0,00	0,00	0,00	0,00	24.777,18	0,00	0,00	0,00	0,00	0,00
morte	0,00	0,00	0,00	0,00	0,00	0,00	0,00	0,00	0,00	0,00	17.554,74	0,00	17.554,74	0,00	17.554,74	0,00	35.109,47	0,00	35.109,47	0,00
**DRC**																				
Incid. e Trat	87.566,08	0,00	43.783,04	0,00	43.783,04	0,00	43.783,04	0,00	0,00	0,00	43.783,04	0,00	0,00	0,00	0,00	0,00	43.783,04	0,00	0,00	0,00
Tratamento	0,00	0,00	81.959,18	0,00	122.938,77	0,00	163.918,36	0,00	204.897,95	0,00	204.897,95	0,00	245.877,54	0,00	245.877,54	0,00	245.877,54	0,00	286.857,13	0,00
Incid e morte	0,00	0,00	0,00	0,00	20.358,19	0,00	20.358,19	0,00	20.358,19	0,00	20.358,19	0,00	20.358,19	0,00	20.358,19	0,00	20.358,19	0,00	20.358,19	20.358,19
morte	0,00	0,00	0,00	0,00	0,00	0,00	17.554,74	0,00	35.109,47	0,00	52.664,21	0,00	70.218,95	0,00	87.773,69	0,00	105.328,42	0,00	122.883,16	0,00
**TOTAL**	**178.220,01**	**48.174,75**	**313.405,77**	**99.382,25**	**393.875,75**	**128.507,33**	**537.969,13**	**155.947,36**	**583.945,09**	**151.196,83**	**719.579,77**	**178.257,71**	**760.309,80**	**240.990,38**	**834.240,40**	**221.672,90**	**934.790,91**	**233.871,29**	**1.003.802,50**	**319.687,91**

Legenda Tabela 15: ATF = Atenção Farmacêutica; sem ATF = assistência à saúde pelo SUS sem a ATF, dados relativos ao período pré ATF; com ATF = assistência à saúde com o programa de ATF, dados relativos aos períodos ATF e pós ATF; HAS = hipertensão arterial sistêmica; AVE = acidente vascular encefálico; DIC = doença isquêmica do coração; IC = insuficiência cardíaca; DRC = doença renal crônica.

O maior custo com as complicações durante os dez anos de projeção foi atribuído à DRC. Os custos dos modelos assistenciais sem e com ATF foram maiores à medida que os anos se passaram, devido a alocação acumulativa dos pacientes em estados de saúde da complicação da HAS. Ressalta-se que em todos os anos de projeção o custo da assistência à saúde com o modelo de ATF foi menor. No final, ao somar os custos de todos os anos, a assistência convencional teve um custo de R$ 6.260.139,13 e a assistência com a ATF de R$ 1.777.688,73.

A assistência à saúde com a ATF foi capaz de salvar anos de vida e aumentar a qualidade dos anos vividos para os pacientes hipertensos diante a mortalidade ocasionada pela HAS e as suas complicações. O maior custo com as complicações da HAS foi atribuído à DRC. Foi verificado que o custo da assistência com a ATF foi menor que o custo da assistência convencional no SUS ao projetar as complicações da HAS em dez anos. O que permitiu calcular a diferença entre os custos como sendo o benefício. Dessa forma, os maiores benefícios obtidos em dez anos foram no sexto e no penúltimo ano. Na análise dos benefícios adquiridos em dez anos foi verificado um VPL positivo, mostrando que o investimento na ATF foi capaz de trazer o retorno monetário, pois economizou custos futuros com as DCVs e DRC.

5.8. Obtenção do valor presente líquido atribuído à economia relativa aos gastos com a hipertensão arterial sistêmica e as suas complicações

Foi realizada a diferença entre os custos calculados na projeção das complicações da HAS para o modelo assistencial convencional e para o modelo com ATF. Essa diferença, quando positiva, aparece na entrada no fluxo de caixa e quando negativa, nos valores de saída. Foi notado que não houve diferença negativa, ou seja, em todos os anos a ATF trouxe benefício monetário. Considerando o ano zero como o ano de 2009, foi observada a saída de R$ 51.518,60, referente ao investimento na ATF. Nota-se que em 2015 (ano 6) e 2018 (ano 9) foram os anos de maiores benefícios, respectivamente, R$ 541.322,05 e R$ 576.648,30.

Calculando o VPL de acordo com a taxa de *discouting*, observa-se um retorno do investimento na ATF igual a R$4.078.396,13, o que significa que a economia gerada em dez anos com o atendimento de 104 pacientes pela ATF representa 4,84% dos custos anuais de Ribeirão Preto para o tratamento de toda a população hipertensa do município. Adicionalmente, ao investir na ATF com o intuito de assistir toda a população hipertensa, o

benefício gerado pelos custos economizados com as complicações da doença ultrapassaram os gastos assistenciais com a HAS. Neste sentido, o município ao reduzir os gastos para tratar as complicações das morbidades em saúde, pode investir na promoção da mesma, ampliando o atendimento e promovendo o cuidado integral aos pacientes, além de possibilitar investimentos em outras morbidades (Figura 16).

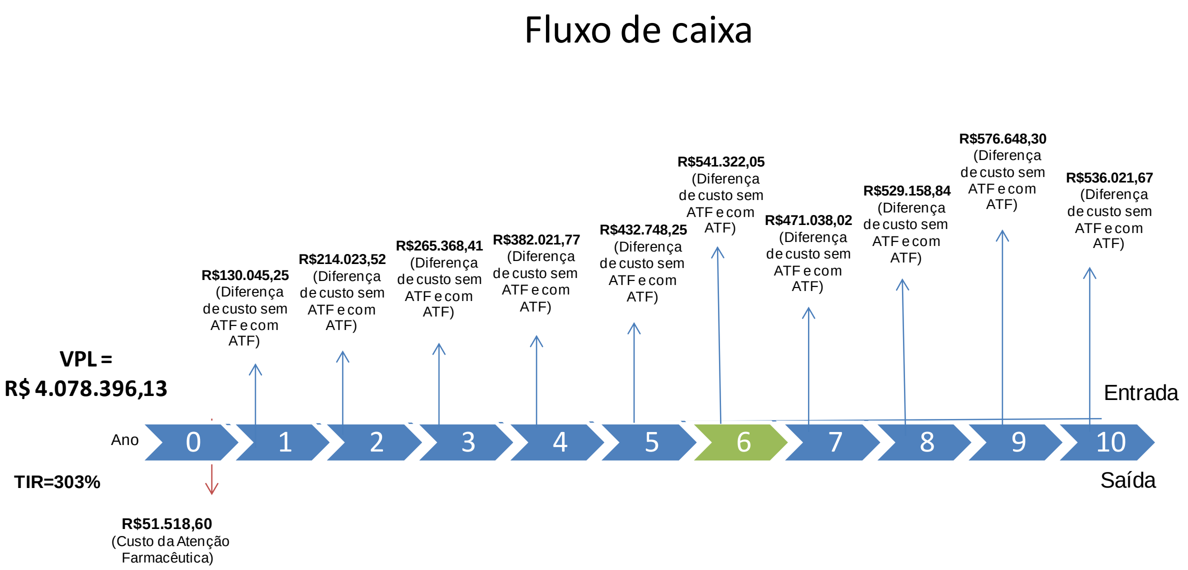

Legenda Figura 16: O ano zero representa o ano de 2009, no qual houve o programa de Atenção Farmacêutica; o ano dez representa o ano de 2019; o ano seis representa 2015, os anos seguintes a 2015 foram ajustados pela taxa de 5% de desconto para remeter ao valor presente, o respectivo *discouting*. VPL = valor presente líquido; TIR = taxa de retorno do investimento.

Figura 16 – Fluxo de caixa referente aos dez anos de projeção de custos com a hipertensão e suas complicações.

Para a análise de custo benefício foi considerando o custo de investimento na ATF de R$ 51.518,60 e o benefício calculado pelo VPL, desta forma, a RBC obtida foi de 79,18, RBC > 1. Isso significa que o investimento na ATF foi custo-benéfico para a saúde. Adicionalmente, de acordo com o VPL, a taxa de retorno do investimento (TIR) foi de 303%, essa taxa representa a taxa de desconto que o fluxo de caixa teria para igualar o VPL à zero. E o *Payback*, tempo que o investimento retorna, foi de um ano, ou seja, no primeiro ano os benefícios já superam os custos, o que permitiu o retorno deste investimento. Um programa de vacinação na Espanha teve a RBC igual a 1,78, suficiente para a implantação ao combate a varicela (DOMINGO et al., 1999). A análise desses dados possibilita considerar a ATF como um investimento rentável em saúde, pois há poucos investimentos que retornam logo no primeiro ano e a TIR pode ser considerada em uma alta taxa neste caso. Para os investimentos do governo, por exemplo, em obras de concessões, é esperada uma TIR em média de 10,6% no ano. O setor imobiliário, um dos setores que mais cresceram no Brasil apresentou uma

TIR, até o ano em que iniciou a recessão econômica no Brasil (2014), igual a 9,7% (BRASIL, 2015q; BRASIL, 2015r).

5.9. Análise de sensibilidade dos custos para o valor presente líquido (VPL)

As diferentes categorias de custos que incorporaram as análises deste estudo podem ser entendidas como variáveis estocásticas para a análise de sensibilidade do VPL. Elas variam de acordo com as probabilidades de uma distribuição a qual sua natureza está sujeita. O custo indireto relativo à invalidez foi sensível às variações referentes ao salário base da população, ou seja, a taxa de trabalhadores e a taxa de invalidez, somados aos custos com encargos sociais que o paciente deixaria de contribuir, variados pela taxa de trabalhadores e taxa de invalidez. O custo indireto relativo à morte teve as variações respectivas ao salário base da população, aos encargos sociais e as taxas de trabalhadores. Os custos da incidência + tratamento sofreram a variação dos custos de internação relativos a cada complicação, do tratamento respectivo a cada complicação da HAS e, da variação da pensão por invalidez. O custo do tratamento foi sensível à variação do custo para tratar cada complicação da HAS pós-evento e aos custos da pensão por invalidez. O custo de incidência + morte foi sensível ao custo de internação relativo a cada complicação somado a variação dos custos com morte.

As distribuições das categorias de variáveis foram definidas de acordo com a natureza de cada variável e com a distribuição dos dados relativo a elas, sendo aplicadas para gerar valores dentro de um intervalo. Sendo assim, a distribuição Pert foi preferida por representar uma variação de probabilidades com maior frequência em torno da média e, menor decaimento da frequência nas extremidades quando comparada à normal padrão. A distribuição de La Place foi escolhida por apresentar um decaimento das probabilidades mais acentuado tanto para o mínimo quanto para o máximo ao redor da média e, a distribuição triangular por ser representativa para a variação de taxas, com valores mínimo, médio e máximo (Tabela 16) (McCULLAGH & NELDER, 1989; FLEURENCE & HOLLENBEAK, 2007).

Tabela 16 – Tabela de descrição das variáveis de custo para a análise de sensibilidade

		Média		minimo		máximo	Distribuição
**Custos complicações da HAS e custos indiretos**							
Salário base população	R$	29.276,26	R$	10.244,00	R$	52.663,52	Pert
Encargos sociais	R$	10.620,87	R$	3.744,00	R$	17.142,84	Pert
Taxa trabalhadores HAS ativos		44,0%		25,0%		60,0%	Triangular
Taxa de Invalidez		56,85%		30%		70%	Triangular
Custo internação AVE	R$	3.591,86		1505,54		7092,89	Laplace
Custo internação DIC	R$	8.937,14		2842,10		21706,68	Laplace
Custo internação DAP	R$	3.262,51		1200,00		3975,49	Laplace
Custo internação IC	R$	7.222,44		5202,54		17346,65	Laplace
Custo internação IRC	R$	2.803,45		1800,00		3373,57	Laplace
Custo tratamento AVE	R$	3.350,10		1675,05		5025,15	Pert
Custo tratamento DIC	R$	4.282,07		2141,03		6423,10	Pert
Custo tratamento DAP	R$	2.365,00		1182,50		3547,05	Pert
Custo tratamento IC	R$	3.365,00		1200,00		5047,50	Pert
Custo tratamento DRC	R$	27.928,68		26532,25		31950,15	Pert
**Custos diretos**							
Exames		0,95%		0,80%		2,50%	Triangular
Consulta basica		34,78%		28,19%		51,29%	Triangular
Consulta especializada		24,72%		22,02%		27,80%	Triangular
Consulta emergência		21,98%		14,91%		31,60%	Triangular
Medicamentos		11,10%		4,24%		30,72%	Triangular
Transporte		1,03%		0,49%		1,16%	Triangular
Absenteísmo		5,45%		3,35%		14,02%	Triangular
**Custos com a ATF**							
Salário Farmacêutico		37680,83		28709,2		71773	Pert
Custos materiais		1963,05		1500		6000	Pert
Unidade para atendimento		11874,72		4355,52		29827,02	Pert

Legenda Tabela 16: ATF = Atenção Farmacêutica; sem ATF = assistência à saúde pelo SUS sem a ATF, dados relativos ao período pré ATF; com ATF = assistência à saúde com o programa de ATF, dados relativos aos períodos ATF e pós ATF; HAS = hipertensão arterial sistêmica; AVE = acidente vascular encefálico; DIC = doença isquêmica do coração; IC = insuficiência cardíaca; DRC = doença renal crônica. O tratamento para as complicações referem-se aos custos anuais com cada complicação para tratar o paciente pós-evento. Os custos diretos variaram de acordo com a sua proporção dentro do cálculo dos custos direto para o tratamento da hipertensão. Os custos referem-se a custos anuais.

De acordo com as variações dos custos foi realizada a simulação de Monte Carlo para 10.000 iterações. Cada ponto do gráfico de dispersão representa uma iteração, ou um valor atribuível ao beneficio de cada ano, o VPL. Nenhum dos valores referentes à simulação de Monte Carlo para dez mil iterações foi negativo nos dez anos, isso mostra que há retorno do investimento na ATF em todos os anos da análise mesmo com as variações de custo. A dispersão nos dez anos concentrou-se em maior proporção entre o segundo e o terceiro quadrante, o que representa a linearidade entre os possíveis valores do benefício obtido com a ATF, ou seja, os dados convergiram na simulação não havendo interferentes. Nota-se que os valores foram mais propensos em todos os anos no quadrante três, o que deslocou a média do

benefício adquirido com a ATF para a esquerda, repercutindo em um valor menor do que a maioria das iterações. Em adição, a sensibilidade do benefício adquirido com a ATF foi sempre a maior que R$100.000,00 de acordo com as 10.000 iterações (Figura 17).

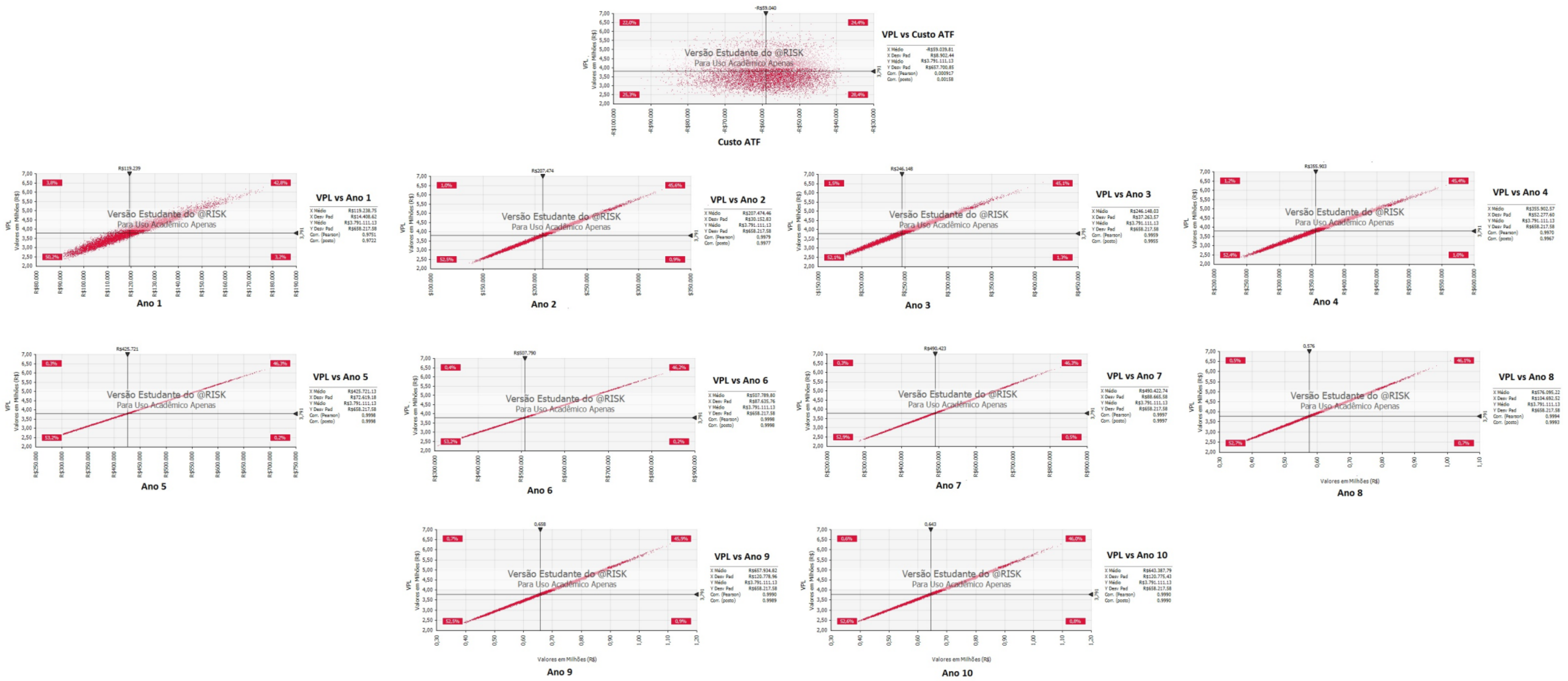

Legenda Figura 17: VPL = valor presente líquido; ATF = Atenção Farmacêutica. Cada plano representa a dispersão dos possíveis valores respectivos a 10.000 iterações segundo a simulação de Monte Carlo.

Figura 17 – Gráfico de dispersão do custo com a Atenção Farmacêutica e o valor presente líquido nos dez anos.

De acordo com a sensibilidade para o VPL na projeção dos dez anos, a média do VPL foi de R$ 3.791.111,13, havendo 95% de probabilidade do VPL estar entre R$ 2.693.000,00 e R$ 5.207.000,00, ou seja, sempre positivo. Considerando o custo de investimento na ATF de R$ 51.518,60 e o benefício calculado pelo VPL, a RBC obtida foi 30,03 (26,74 – 34,28), RBC > 1. Adicionalmente, há a probabilidade de 100% para o menor valor do VPL em todas as iterações ser maior que R$ 2.268.869,48. Ressalta-se que o limiar de sensibilidade de 97% de probabilidades favoráveis à economia de recursos financeiros, utilizando 10.000 iterações de Monte Carlo, foi suficiente para a inserção de uma TS na Espanha (CALVET et al., 2012). Mantendo o intervalo de 95% de probabilidade para os valores de VPL e iniciando a probabilidade do seu menor valor, é possível verificar que o VPL reduz de R$ 5.207.000,00 para R$ 4.966.000,00. O que mostra a ATF como um investimento seguro quanto ao benefício esperado, mesmo havendo variações nos custos (Figura 18).

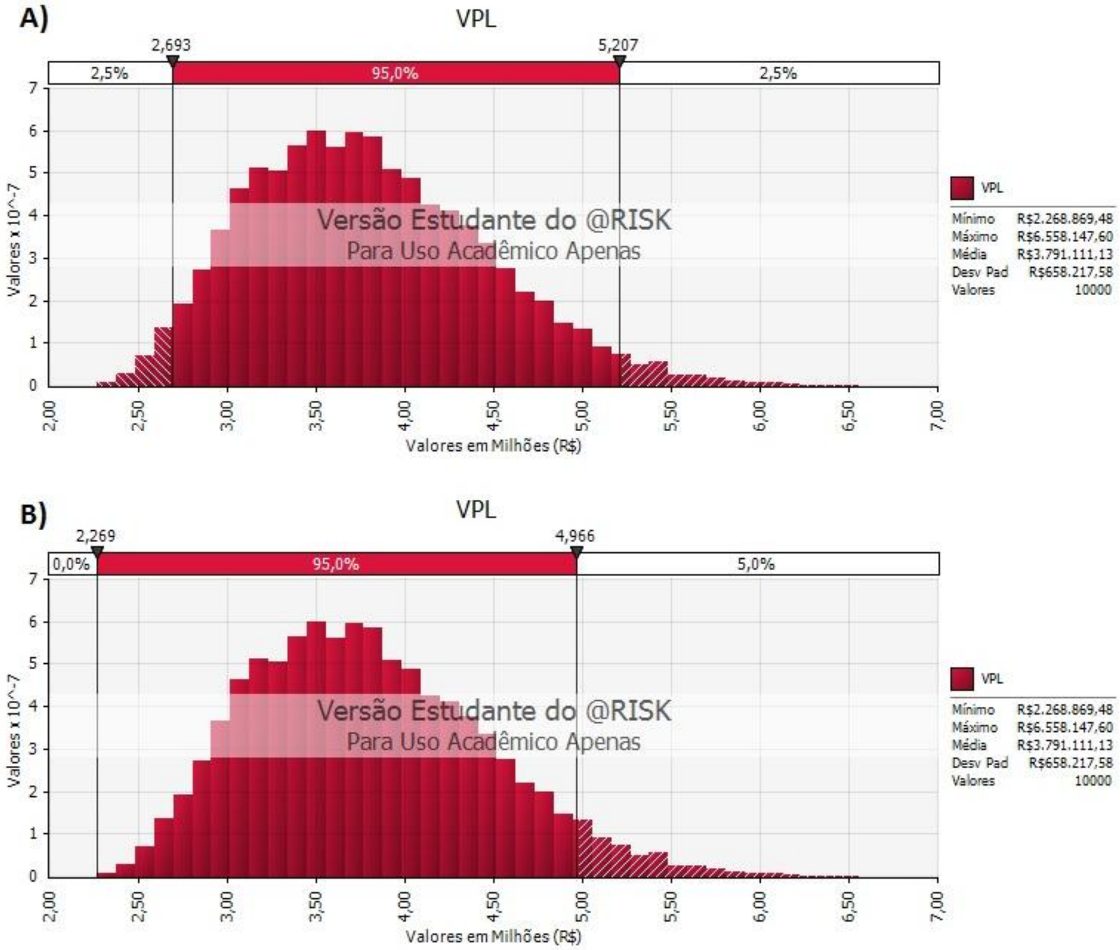

Legenda Figura 18: VPL = valor presente líquido. A parte **A)** desta figura representa a densidade de probabilidades para o valor presente líquido em dez anos para um intervalo de confiança de 95% bicaudal e a parte **B)** para um intervalo de confiança de 95%, considerando o menor valor de VPL possível.

Figura 18 – Gráfico de densidade de probabilidades para o valor presente líquido.

Das categorias de custo mais influentes na variação do VPL estão o salário base da população e a taxa de trabalhadores ativos com HAS. Essas duas categorias são capazes de alterar o VPL de R$ 3.035.252,35 – R$ 4.586.718,76 e R$ 3.145.962,50 – R$ 4.415.010,83, respectivamente. Essas categorias de custo, juntamente com a taxa de invalidez e os encargos sociais formam as quatro categorias mais influentes no valor do VPL. Elas compõem o custo indireto com a saúde, pensão por invalidez e pensão por morte. Os custos com a perda de produtividade são considerados por Ferreira, Costa e Souza (2011) como aqueles de maior impacto na economia (Figura 19).

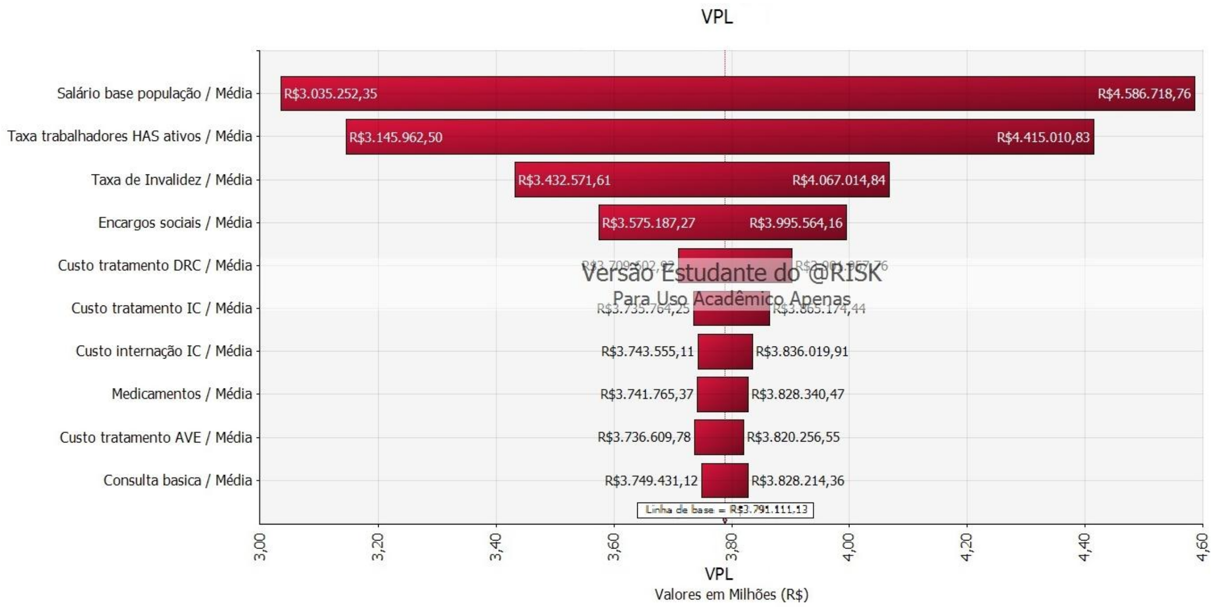

Legenda Figura 19: VPL = valor presente líquido. A figura descreve cada variável de custo e sua respectiva influência no VPL, dimensionada pelas barras do gráfico.

Figura 19 – Diagrama de Tornado para a influência das variáveis de custo no valor presente líquido.

Ao analisar a sensibilidade para o VPL ao longo dos dez anos é possível notar que sempre houve benefício com o investimento na ATF e esse sempre foi maior que R$ 100.000,00, o que é visto ao analisar o limite inferior para o intervalo de confiança de 95%. A variação aumentou com o passar dos anos, o que ocorreu, provavelmente pelo aumento do retorno do investimento (Figura 20).

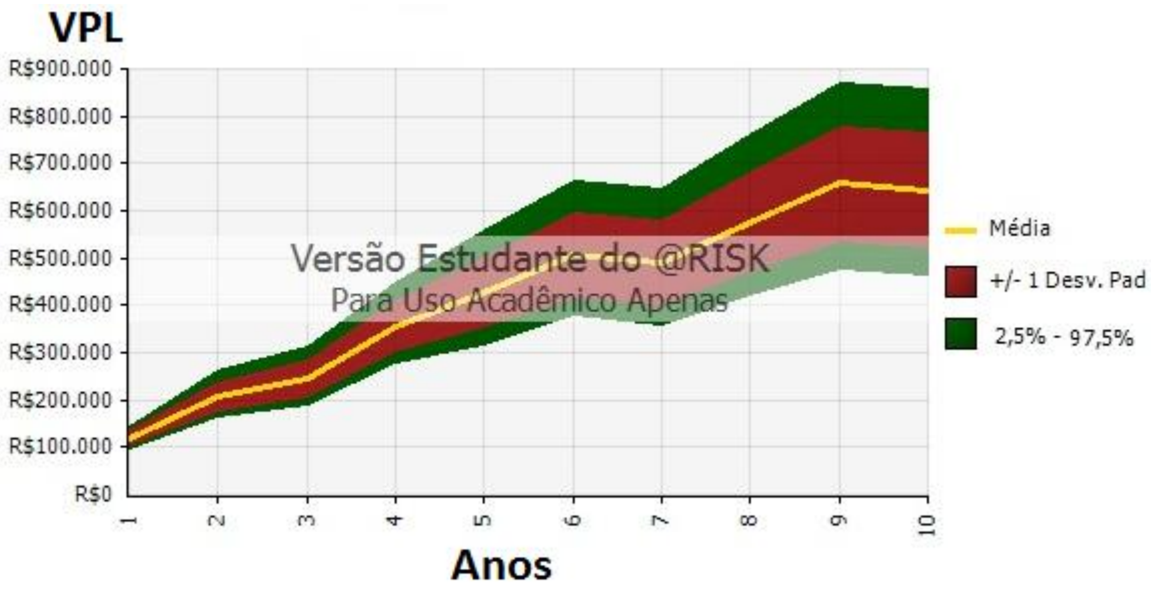

Legenda Figura 20: VPL = valor presente líquido. A figura descreve a variação da média do VPL ao longo de dez anos, com os respectivos desvios padrão e intervalo de confiança de 95%.

Figura 20 – Gráfico de tendência do valor presente líquido ao longo de dez anos.

Considerando apenas os custos diretos à saúde, retirando os custos mais influentes na análise do VPL (custos indiretos), obtém-se a média do VPL menor que a calculada com todas as categorias de custos. Entretanto, a média e o valor mínimo foram positivos R$ 1.547.486,77 e R$ 1.377.734,68, respectivamente. Considerando o custo de investimento na ATF de R$ 51.518,60 e o benefício calculado pelo VPL, a RBC obtida foi de 30,04 (26,74 – 34,28), RBC > 1 (Figura 21).

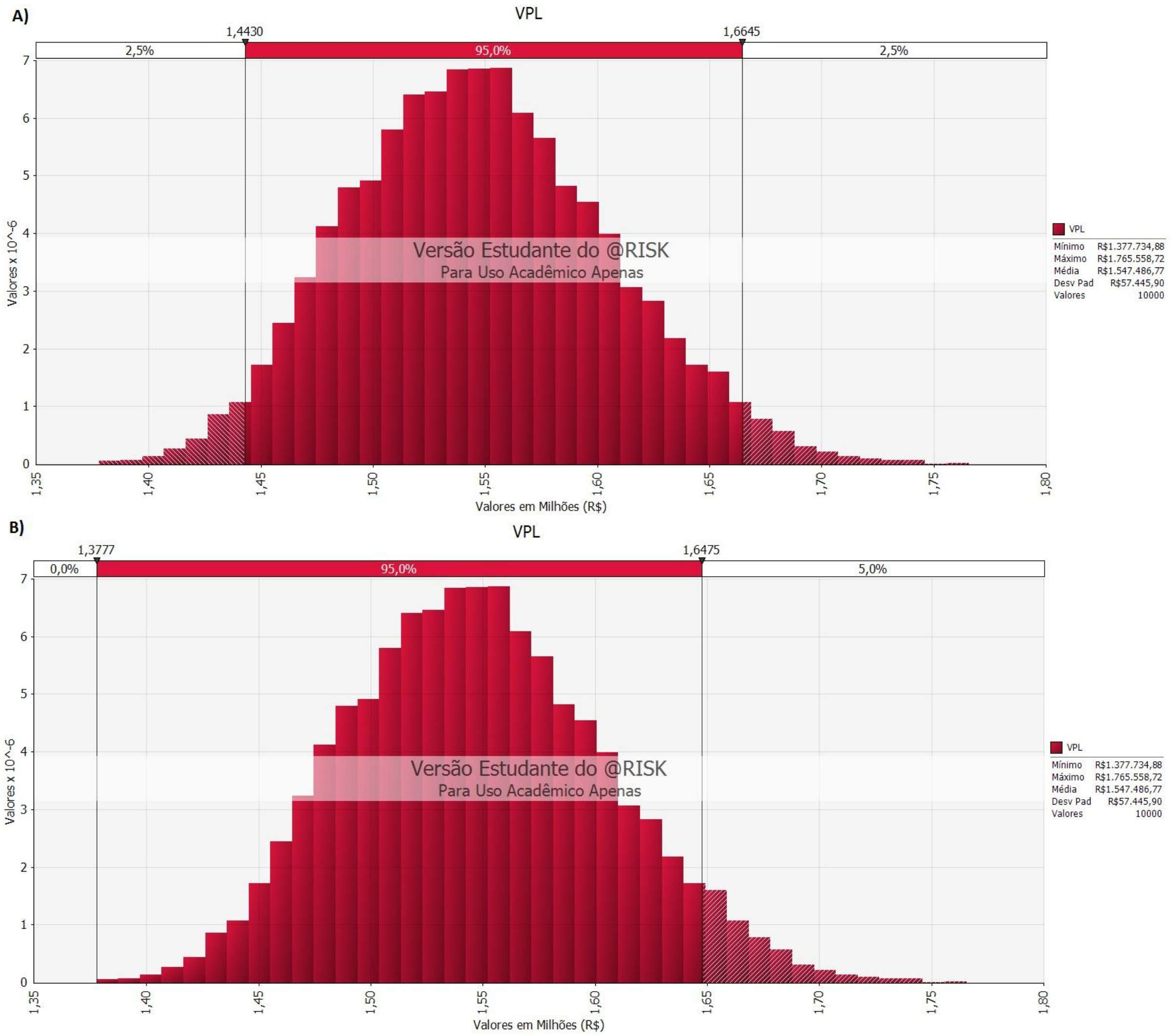

Legenda Figura 21: VPL = Valor Presente Líquido. A parte **A)** desta figura representa a densidade de probabilidades para o valor presente líquido em dez anos para um intervalo de confiança de 95% bicaudal e a parte **B)** para um intervalo de confiança de 95%, considerando o menor valor de VPL possível. Ambos remetem ao VPL sem os custos indiretos.

Figura 21 – Gráfico de densidade de probabilidades para o valor presente líquido sem os custos indiretos.

Dentre as categorias de custos diretos à saúde mais influentes na variação do VPL, o tratamento da DRC e da IC provocaram a maior variação, R$ 1.479.644,42 – R$ 1.632.132,66 e R$ 1.495.208,87 – R$1.597.169,38, respectivamente. Nenhuma categoria de custo provocou variação no VPL para valores negativos, o que representa que sempre houve benefício com o investimento na ATF (Figura 22).

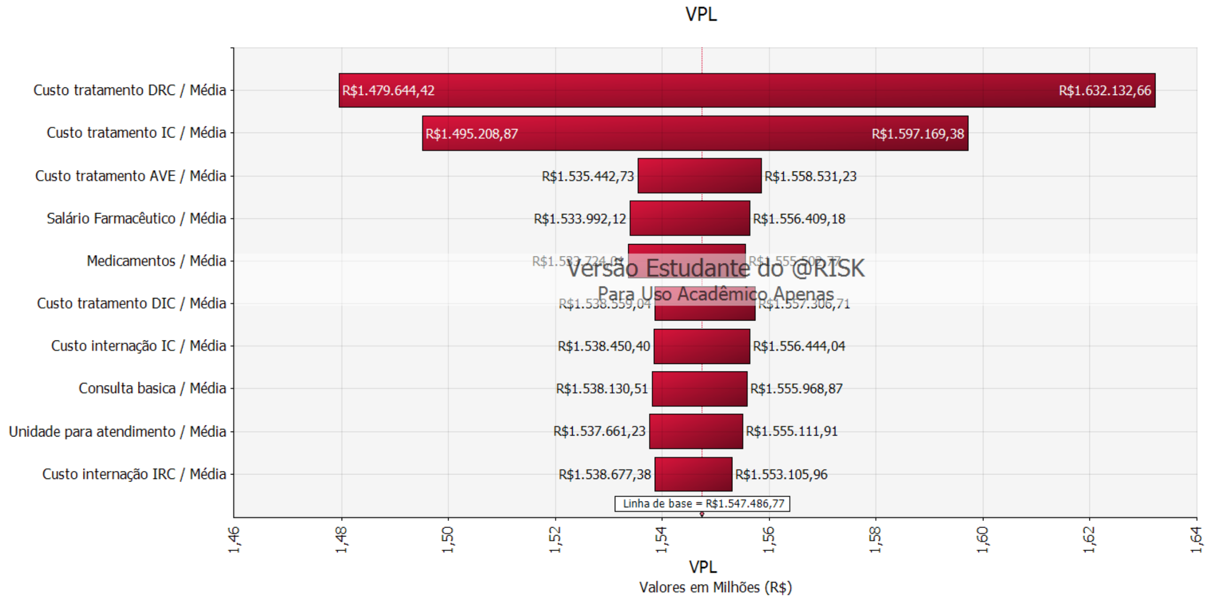

Legenda Figura 22: VPL = valor presente líquido. A figura descreve cada variável de custo e sua respectiva influência no VPL, dimensionada pelas barras do gráfico. A variável unidade para atendimento refere-se a unidade para atendimento Farmacêutico na ATF, o que destina aos custos da consulta farmacêutica.

Figura 22 – Diagrama de Tornado para a influência das variáveis de custo no valor presente líquido na ausência dos custos indiretos.

É possível notar que o intervalo de confiança para o VPL diminui ao retirar os custos indiretos da análise. Mesmo diminuindo os valores do VPL, todos os anos apresentaram benefício e ele sempre foi progressivo e maior que R$ 60.000,00, ou seja, maior que o investimento inicial na ATF, que foi de R$ 51.518,60 (Figura 23).

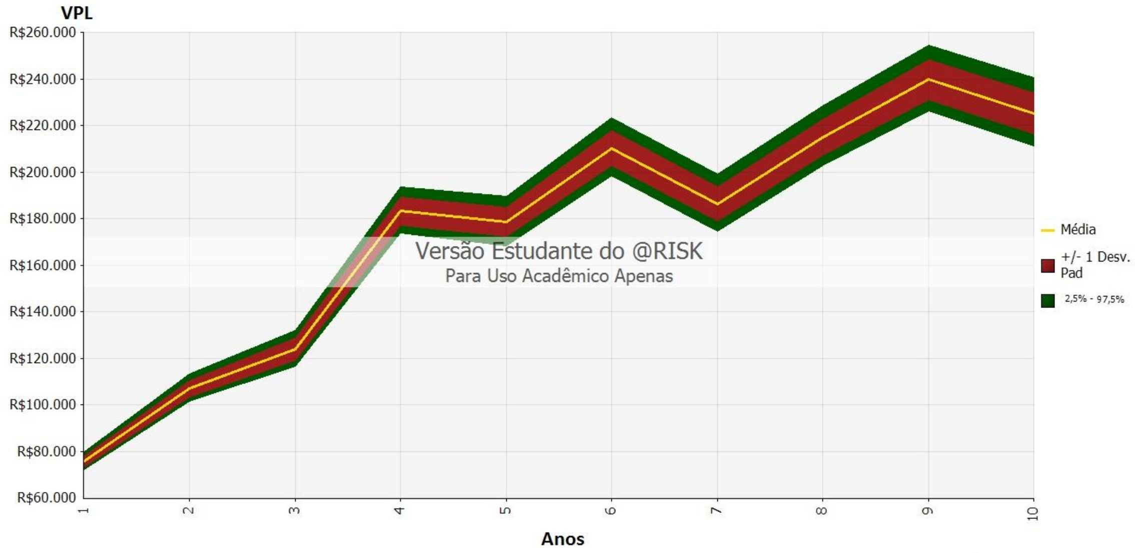

Legenda Figura 23: VPL = valor presente líquido. A figura descreve a variação da média do VPL sem a influência dos custos indiretos ao longo de dez anos, com os respectivos desvios padrão e intervalo de confiança de 95%.

Figura 23 – Gráfico de tendência do valor presente líquido sem influência dos custos indiretos ao longo de dez anos.

Ressalta-se que os custos indiretos representam em média 58% dos custos em saúde (KULASINGAM & MYERS, 2003), como a DRC e a IC representaram as variáveis de custo de maior influência no VPL, simulando as probabilidades sem essas variáveis e sem os custos indiretos verifica-se que há possibilidade do VPL ser negativo, ou seja, pode não haver o retorno do investimento. Contudo, é possível afirmar que há 12,4% de probabilidade do VPL ser positivo, e o investimento na ATF apresentar benefício monetário. Ocorrendo isso, o benefício pode ser de até R$ 40.000,00. De toda forma, mesmo retirando os custos mais influentes ao VPL para o tratamento da HAS, nota-se que a ATF foi capaz de reduzir em R$31.380,98, remetendo a um investimento na ATF de R$ 20.137,62 (Figura 24). Isso representa um custo real da ATF de R$ 656,85 / paciente no período da ATF, R$ 190,68 a mais que a assistência convencional e R$ 301,00 a menos do que o custo / paciente no período ATF calculado na análise direta. O que significa uma RCE de R$ 668,89 / controle pressórico e uma RCEI de R$ 436,02 por paciente com controle pressórico a mais, quase um terço do valor da $RCEI_{ATF}$ calculada na análise direta de custos.

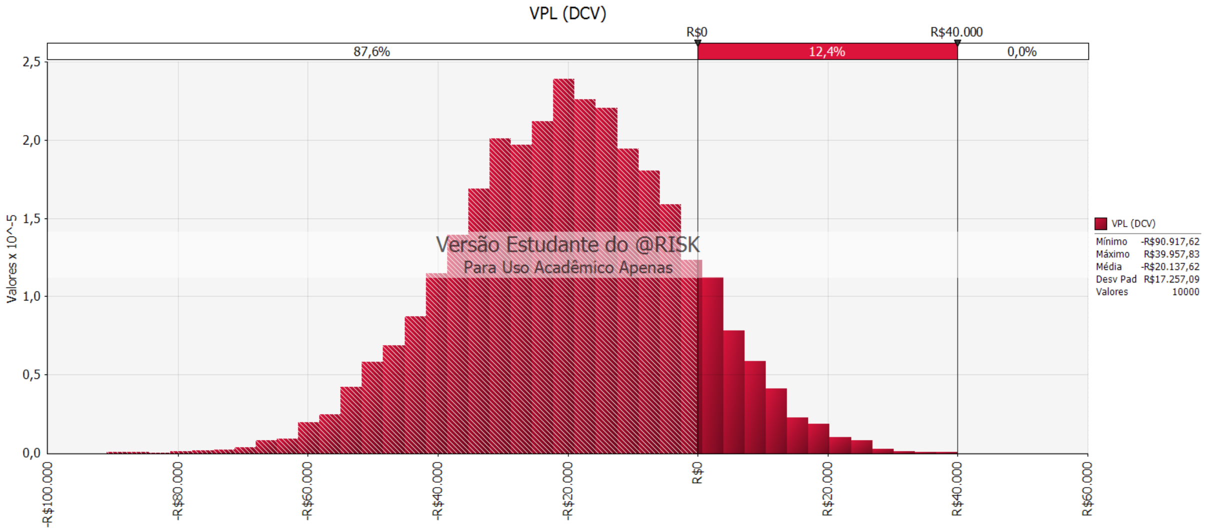

Legenda Figura 24: VPL = valor presente líquido; DCV = doença cardiovascular. Esta figura representa a densidade de probabilidades para o valor presente líquido em dez anos sem os custos indiretos e somente com a projeção para as DCVs.

Figura 24 – Gráfico de densidade de probabilidades para o valor presente líquido sem os custos indiretos.

5.10. Impacto epidemiológico da Atenção Farmacêutica quanto ao controle pressórico e as complicações da hipertensão

O risco relativo foi igual a 6,5 (2,43-17,44) com *p* valor < 0,0001, o que mostra que os pacientes quando não recebem a assistência com ATF apresentam maior probabilidade de não controlar a PA. O paciente com a assistência convencional teve a probabilidade 6,5 vezes

maior de não controlar a PA. Ainda, com 95% de certeza é possível afirmar, considerando o limiar mínimo, que o paciente quando não foi assistido pelo programa de ATF possuiu 2,43 vezes maior a probabilidade de não ter a pressão arterial controlada. O OR calculado foi 11,1 (3,5-34,8) com *p* valor < 0,0001, o que significa que o paciente recebendo o tratamento convencional do SUS teve 11,1 vezes mais chance de não controlar a PA que o indivíduo que foi assistido pela ATF, e com uma sensibilidade de, no mínimo, 3,5 vezes mais chance de não controlar a PA para o intervalo de confiança de 95%.

Quanto às complicações cardiovasculares, o risco relativo foi igual a 3,1 (1,83-5,13) com *p* valor < 0,0001, o que representa que o paciente sem receber a assistência com ATF tem maior probabilidade de apresentar as complicações da HAS. Ainda, com 95% de certeza é possível afirmar para o mínimo provável é 1,8 vezes maior que a probabilidade de o paciente apresentar complicações da HAS quando não é assistido pelo programa de ATF. O OR foi 4,7 (2,40-9,19) com *p* valor < 0,0001, o que significa que o paciente recebendo o tratamento convencional do SUS teve 4,7 vezes mais chance de apresentar complicações da HAS que o indivíduo que é assistido pelo tratamento que incorpore a ATF. Analisando a sensibilidade para o intervalo de confiança de 95%, os pacientes não assistidos pela ATF apresentam no mínimo 2,4 vezes mais chance de apresentar complicações da HAS.

É importante notar que intervalo de confiança amplo, tanto na análise do controle pressórico quanto das complicações da doença, remetem ao número amostral baixo, porém pode ser observado que o valor mínimo em 95% de confiança para o OR e RR é maior que 1 com *p* valor < 0,0001, o que mostra evidência da associação da ATF tanto para o controle pressórico, quanto para evitar as complicações da HAS. O percentual de RRR para o controle pressórico foi 84,6% e para as complicações da HAS foi 67,74%, o que representa que ao aplicar a ATF para toda uma população é possível reduzir em 84,6% o número de indivíduos com a PA em nível pressórico insatisfatório, e em 67,74% a incidência de complicações da HAS. Ressalta-se que para as complicações da HAS esses indicadores epidemiológicos referem aos dados acumulados na projeção de dez anos e para o controle da PA anual. O impacto epidemiológico mensurado pela RRR é atribuído como indicador de eficácia da intervenção em ensaios clínicos. Para estes tipos de estudos são considerados valores acima de 30% para a recomendação da intervenção quanto sua eficácia (COUTINHO & CUNHA, 2005).

5.11. Ferramenta farmacoeconômica

Com a análise de sensibilidade para a projeção de custos sem os custos indiretos e sem os custos da DRC e IC, tem-se uma situação na qual a projeção para o tratamento da HAS não possui todas as variáveis influentes no custo deste tratamento. A ferramenta elaborada incorporou todas as variáveis de custo deste trabalho. Ressalta-se que foi utilizado o valor do VPL de cada ano referente ao limite inferior do intervalo de confiança de 95%. Todavia, para o custo com a ATF o limite superior foi considerado. Dessa forma, a ferramenta elaborada é representativa do retorno mínimo que se tem com o investimento no programa de ATF para pacientes com HAS.

Para estruturar a ferramenta ainda foi necessário utilizar a RRR e o NNT calculados na etapa 3 e 4 deste trabalho. O NNT representou na ferramenta o número de indivíduos que tiveram complicações da HAS evitadas e a RRR o número de indivíduos com a pressão arterial controlada após receberem a ATF. Com isso, a ferramenta de análise econômica é capaz de auxiliar na tomada de decisão do gestor quanto aos indicadores epidemiológicos da sua região / município e quanto ao valor que o mesmo possui para o investimento na ATF, projetando a economia a ser gerada em dez anos, pelo investimento na ATF e, assim, possibilitar o planejamento para a alocação de recursos e para a repercussão da melhora à assistência a saúde para outras morbidades.

Nesse sentido, a ferramenta possui uma interface onde inicialmente há três campos a ser preenchido, o campo “inflação acumulada (base 2015)”, no qual o usuário colocará o valor da taxa de inflação, caso a ferramenta seja utilizada em anos posteriores a 2015, por exemplo, dois anos após 2015, deve-se colocar a taxa acumulada de inflação destes dois anos. Outro campo é o “investimento inicial”, no qual o gestor colocará o valor que se tem a investir na estruturação da ATF em sua região ou município. E o terceiro campo, de preenchimento automático, é o campo “perda de recurso”, que mostra o quanto do valor que se pretende investir na ATF é obsoleto, ou seja, está sendo a mais para a estruturação do serviço. Assim, o gestor pode economizar ou realocar este recurso, ou ainda, tomar a decisão de complementá-lo e ampliar a abrangência da ATF em seu município ou região.

Ao preencher os campos iniciais aparecerá o valor da perda, se houver. Depois, a ferramenta mostra o cálculo do número de farmacêuticos necessários a contratar para desenvolver a ATF. Por conseguinte, apresenta os resultados anuais dos pacientes que serão

assistidos pela ATF, do número de complicações da HAS que serão evitadas e do retorno do investimento. No final, a ferramenta mostra os resultados dos pacientes atendidos, as complicações evitadas e os pacientes com a PA controlada resumidos em dez anos, assim como o VPL em dez anos é apresentado como o retorno do investimento no valor presente. Um gráfico de economia de recursos financeiros é gerado mostrando a repercussão no decorrer dos anos (Figura 25).

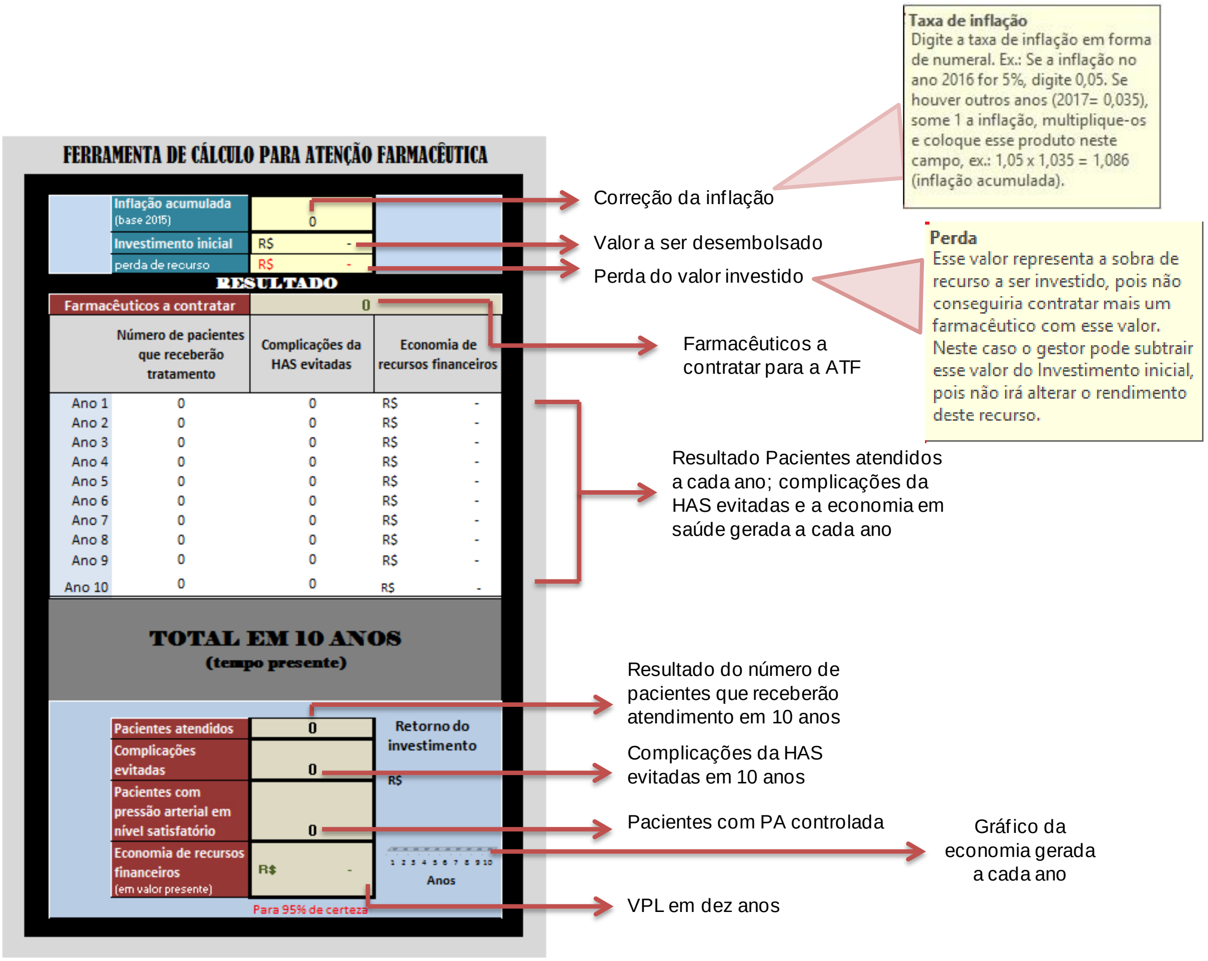

Legenda Figura 25: VPL = valor presente líquido; ATF = Atenção Farmacêutica; HAS = hipertensão arterial sistêmica; PA = pressão arterial. A calculadora é dinâmica, ao digitar nos três campos de entrada os resultados automaticamente aparecem. É uma ferramenta que funciona no Microsoft Excel ®, pacote *office*.

Figura 25 – Ferramenta farmacoeconômica para auxiliar a tomada de decisão aos gestores de saúde para a implantação da Atenção Farmacêutica.

Utilizando um exemplo de um investimento de R$ 220.000,00 anuais em Atenção Farmacêutica a ferramenta farmacoeconômica calcula que se deve contratar três farmacêuticos e que sobrará R$ 16.116,94 em recursos do investimento. Com esses três farmacêuticos a calculadora mostra que no primeiro ano 312 pacientes serão atendidos e haverá uma economia de R$ 110.876,34 em recursos de saúde e assim por diante até dez anos.

No resumo total, em dez anos, a ferramenta mostra o número de pacientes atendidos, no caso 3.120, o número de complicações evitadas, 567, e mostra ainda, que dos 3.120 pacientes 2.640 estarão com a PA controlada e haverá com 95% de certeza um VPL de R$ 6.186.175,05 em dez anos, o que representa no valor presente o lucro dos R$ 220.000,00 investidos (Figura 26).

FERRAMENTA DE CÁLCULO PARA ATENÇÃO FARMACÊUTICA

Inflação acumulada (base 2015)	0
Investimento inicial	R$ 220.000,00
perda de recurso	R$ 16.116,94

RESULTADO

Farmacêuticos a contratar		3	
	Número de pacientes que receberão tratamento	Complicações da HAS evitadas	Economia de recursos financeiros
Ano 1	312	12	R$ 110.876,34
Ano 2	624	36	R$ 643.012,65
Ano 3	936	66	R$ 1.175.086,50
Ano 4	1248	108	R$ 2.086.005,93
Ano 5	1560	162	R$ 3.146.784,12
Ano 6	1872	231	R$ 4.406.673,33
Ano 7	2184	300	R$ 5.611.479,39
Ano 8	2496	381	R$ 7.026.523,62
Ano 9	2808	474	R$ 8.637.832,62
Ano 10	3120	567	R$ 10.204.848,24

TOTAL EM 10 ANOS
(tempo presente)

Pacientes atendidos	3120
Complicações evitadas	567
Pacientes com pressão arterial em nível satisfatório	2640
Economia de recursos financeiros (em valor presente)	R$ 6.186.175,05

Para 95% de certeza

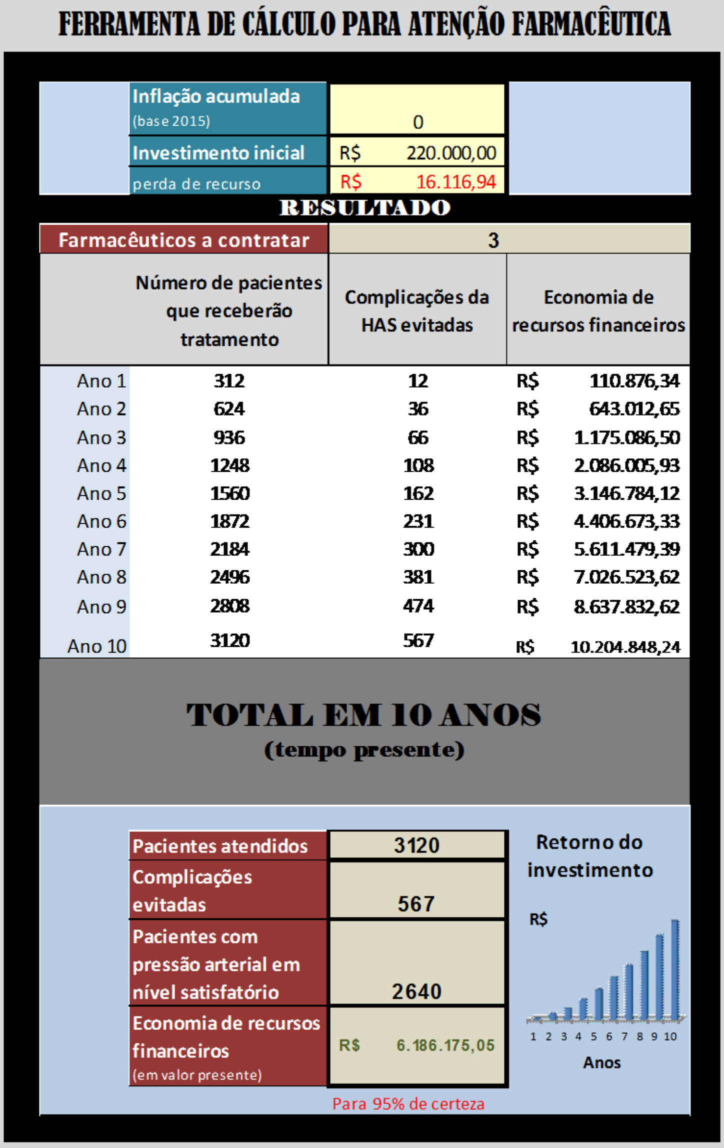

Legenda Figura 26: VPL = valor presente líquido; HAS = hipertensão arterial sistêmica. A calculadora é dinâmica, ao digitar nos três campos de entrada os resultados automaticamente aparecem. É uma ferramenta que funciona no Microsoft Excel ®, software *office*.

Figura 26 – Exemplo do funcionamento da Ferramenta farmacoeconômica para auxilio da tomada de decisão.

Esses resultados permitem refletir sobre a situação econômica do Brasil. A recessão econômica que o país tem passado, iniciada em 2014, agrava ainda mais o quadro da saúde brasileira, uma vez que os custos tendem a aumentar com a correção da inflação e os recursos

para a aplicação financeira diminuir, em decorrência da retração do PIB, no último ano (2015), por exemplo, conforme divulgado pelo Banco Central do Brasil o PIB foi de -4,5% (BRASIL, 2013b; BRASIL, 2015p; BRASIL, 2015r). Nesse sentido, otimizar os recursos torna-se a opção mais assertiva para não diminuir a qualidade da assistência à saúde e manter o funcionamento saudável do SUS. Para isso, uma TS como a ATF torna-se uma opção segura, pois como mostrado nas análises deste estudo, é possível economizar nos gastos com a saúde e fazer com que essa economia seja aplicada na própria saúde ou em outros setores do SUS, bem como, na assistência às outras morbidades.

Em adição, os resultados deste estudo mostraram que a ferramenta desenvolvida é capaz de auxiliar os gestores de saúde no processo de tomada de decisão. Ela possibilita o planejamento para o investimento em programas de ATF e, com isso, melhorar o índice de controle pressórico satisfatório na população hipertensa, reduzindo a morbi-mortalidade ocasionada pela HAS. Adicionalmente, é possível reduzir os gastos futuros em saúde relacionados à esta morbidade e as suas complicações.

É preciso destacar que devido o SUS ser organizado de forma descentralizada e hierarquizada, o financiamento para a incorporação da ATF pode vir por repasse relativo ao bloco da Assistência Farmacêutica. Entretanto, a aplicação será proveniente dos recursos municipais, a esfera municipal terá que planejar a aplicação dos recursos em toda a Assistência Farmacêutica (OLIVEIRA, BERMUDEZ, CASTRO, 2007; BRASIL, 2011). Com o planejamento adequado para a implementação desta TS é possível melhorar os indicadores de saúde do município e economizar recursos. Porém, grande parte dos recursos economizados será da União, mesmo sendo essa esfera a provedora de recursos financeiros, já que os maiores gastos economizados em saúde neste estudo foram de atribuições da União, como: pensão por morte, pensão por invalidez e redução da população economicamente ativa. Assim, ressalta-se que esta ferramenta farmacoeconômica tende a auxiliar na aplicação de recursos pela esfera municipal e também no cálculo para o repasse da União.

Contudo, esse estudo possui algumas limitações. A amostra não otimizou o percentual de pacientes com nível pressórico insatisfatório, conforme mostrado pela literatura. Seria importante no desenvolvimento da ATF no país utilizar instrumentos para a análise de risco do paciente, e assim, criar os critérios para o recrutamento e para a alta do mesmo no determinado programa (GIROLINETO, 2015). O que seria capaz de tornar o investimento na ATF ainda mais rentável e seguro. Neste estudo, este fato pôde acarretar em menor diferença

entre os desfechos de controle pressórico e o risco cardiovascular, sendo possível revelar uma análise de custo-efetividade menos pronunciada ao analisar a ATF. Ainda, os indicadores epidemiológicos, bem como o retorno de investimento, podem ter sido mensurados em valores abaixo do valor real que poderiam ser alcançados pela ATF. Contudo, mesmo com essas limitações, a ATF foi custo efetiva e foi obtido o retorno de investimento acima do valor investido nesta TS, sendo possível destacar que, para os baixos limiares de retorno, foi possível obter uma TIR considerada alta.

Outro ponto importante é que na coleta dos dados não houve o contato com o paciente, o que implicou na ausência de dados para 49,0% dos pacientes, fato atribuído à falta de registros em prontuários e no sistema informatizado. Entretanto, o número de pacientes com dados analisados no estudo ficou dentro do planejamento amostral. Adicionalmente, a amostra do estudo remeteu a população de hipertensos do país permitindo a extrapolação dos resultados. Isso dá subsídios à ferramenta desenvolvida neste estudo para ser aplicável à implementação da ATF no SUS, pois é capaz de auxiliar os gestores de saúde, calculando os indicadores epidemiológicos e o retorno financeiro relativo ao valor que se tem para investir na ATF. Todavia, deve ser considerada uma ferramenta norteadora e não precisa, pois os benefícios calculados foram relativos ao limiar mínimo da análise de sensibilidade, podendo haver um retorno de investimento maior. Em adição, ressalta-se que o planejamento da saúde pode ser fundamental para os resultados desta ferramenta, pois existem falhas no sistema de saúde que podem permitir um baixo rendimento do farmacêutico e uma assistência inadequada para o perfeito funcionamento do programa de ATF.

Há limitações que podem ser consideradas para a aplicação desta ferramenta. Atualmente, os farmacêuticos capacitados, seguros e aptos a desempenhar a ATF são escassos no país. O ensino superior de Farmácia tem sido considerado insuficiente na preparação desse profissional com foco na formação clínica, o que releva a necessidade da reforma nos currículos e o investimento na educação farmacêutica (REIS et al., 2015). Esse é um dos pontos que torna a ATF incipiente no Brasil e que precisa ser melhorado, para que, junto aos resultados apresentados neste trabalho, o gestor possa ter a confiança, não apenas no modelo de prática farmacêutica, ou nos cálculos e nas análises econômicas, mas principalmente, no recurso humano que está se investindo.

Entretanto, a profissão farmacêutica vem sendo reestruturada para a formação de um profissional mais preparado ao cuidado integral da saúde no país. Recentemente, em 2015, foi

proposto pelo Ministério da Saúde, um novo modelo de Assistência Farmacêutica que substitui, entre outras medidas, o termo "Atenção Farmacêutica" por "Cuidado Farmacêutico". Essa modificação não se restringe apenas a nomenclatura, mas principalmente, amplia as ações clínicas do farmacêutico, individualmente ou em conjunto com a equipe de saúde, com foco no uso racional de medicamentos e na educação em saúde. O Cuidado Farmacêutico ao paciente visa promover a utilização adequada dos medicamentos e a melhora dos resultados em saúde, vislumbrando a promoção, proteção e recuperação da saúde e a prevenção de agravos. Tal fato tende a repercutir na formação acadêmica, tornado o farmacêutico um profissional mais apto à prática clínica e ao desenvolvimento da ATF. O que poderá fazer da ferramenta elaborada neste estudo um instrumento fundamental para a promoção da ATF no Brasil (BRASIL, 2014c).

Para isso é preciso considerar que a elaboração desta ferramenta é uma etapa inicial para sua aplicação, pois antes da sua aplicação ela precisa ser desenvolvida e validada. O que se justifica por ela ser baseada em dados clínicos de somente um programa de ATF para hipertensos e por haver a necessidade de aprovação do seu uso na prática profissional por gestores de saúde. Dessa forma, a continuidade deste estudo será fundamental para sua aplicabilidade, pois com a realização de uma revisão sistemática sobre a ATF para pacientes hipertensos e a utilização de técnicas de validação, será possível reestruturar a ferramenta farmacoeconômica com a metodologia desenvolvida neste estudo e conferir robustez para sua utilização.

Espera-se que este estudo encoraje a implementação da ATF no SUS e consiga com isso, reduzir a morbi-mortalidade associada à HAS. Ainda, fomente o aprimoramento e a capacitação do farmacêutico para a prática clínica e seja subsídio para a reestruturação curricular da profissão. Almeja-se que este trabalho repercuta em análises econômicas de outros programas de ATF, a fim de conferir maior robustez para a análise dos resultados desta prática e colaborar para o desenvolvimento de revisões sistemáticas e meta-análises em auxílio ao meio científico e da temática da Medicina Baseada em Evidências. Outrossim, este estudo desenvolveu um novo método de análise farmacoeconômica e espera-se com isso, o fortalecimento de pesquisas na área e também a contribuição com o meio científico na prospecção de novos trabalhos e na ampliação e divulgação do conhecimento nesta área, já que existem poucos estudos farmacoeconômicos de análises em longo prazo e capazes de avaliar o retorno de investimento em programas de saúde (ZUCCHI & FERRAZ, 2010; ISPOR, 2015).

6. CONCLUSÃO

Com a análise dos resultados deste estudo é possível concluir que:

- A ATF foi capaz de promover o empoderamento do paciente hipertenso para o cuidado da sua condição de saúde e do seu tratamento propiciando a melhora dos resultados clínicos, da adesão ao tratamento e da qualidade de vida. A ATF foi capaz de aumentar o controle pressórico, havendo evidência para a melhora da PAS, PAD e CT, e isso fez com que reduzisse o risco cardiovascular. A melhora dos resultados clínicos propiciou uma assistência à saúde mais adequada aos cuidados do paciente hipertenso, diminuindo as crises hipertensivas e promovendo o cuidado preventivo.
- Cerca de um terço dos recursos empregados em saúde foram destinados ao tratamento da HAS em Ribeirão Preto-SP. Ressalta-se que a melhora dos resultados clínicos repercutiu nos custos do paciente hipertenso ao SUS. Foi possível evidenciar na análise direta dos custos que a ATF promoveu a redução dos custos com o paciente hipertenso, mesmo que, após a alta, os custos para o tratamento do paciente hipertenso tenham aumentado ele foi acompanhado da melhora dos resultados clínicos.
- Ao analisar a melhora dos resultados clínicos em associação ao aumento dos custos foi verificado que os custos empenhados a mais para a assistência à saúde com a ATF foram compensados pelos desfechos. Isso mostrou que a ATF foi custo efetiva para o controle da PA no cuidado dos pacientes hipertensos. Ainda, foi possível evidenciar com a análise de sensibilidade dos custos diretos que a ATF foi custo efetiva no período após a alta dos pacientes.
- O maior custo com complicações da HAS foi atribuído à DRC. Foi verificado que o custo da assistência com a ATF foi menor que o custo da assistência convencional no SUS quando projetada a ocorrência das complicações da HAS em dez anos. A ATF reduziu o número de complicações cardiovasculares e por DRC, reduziu ainda o número de óbitos.
- A assistência à saúde com a ATF foi capaz de salvar anos de vida e aumentar os anos de vida ganhos com qualidade para os pacientes hipertensos diante a mortalidade ocasionada pela HAS e as suas complicações. A ATF foi custo efetiva para os anos de vida ajustados pela qualidade, sendo capaz de melhorar a qualidade de vida do paciente hipertenso ao longo de dez anos.

- Houve diferença positiva de custos entre a assistência convencional e a assistência com ATF, o que foi considerado como benefício. Na análise dos benefícios adquiridos em dez anos foi verificado um VPL positivo, mostrando que o investimento na ATF é capaz de trazer retorno monetário, pois economiza custos futuros com as DCVs e DRC.
- A assistência à saúde com a ATF mostrou ser capaz de reduzir o risco do controle pressórico insatisfatório e das complicações da HAS, bem como de reduzir as chances do paciente hipertenso ter a PA em níveis insatisfatórios e ter complicações da HAS. O que permite associar a ATF ao cuidado do paciente hipertenso como um fator de proteção, revelando um impacto positivo epidemiológico com esta TS.
- A análise de sensibilidade mostrou que o VPL foi sensível a variação de custos e manteve-se positivo em todas as possibilidades de variação. Com isso, pode-se concluir que a ATF é capaz de trazer o retorno monetário mesmo com a variação dos custos analisados. O que permite reduzir os gastos em saúde, em longo prazo, contribuindo para a otimização da aplicação dos recursos em saúde.
- Os custos que mais atribuíram variação no VPL foram os custos indiretos à saúde. Quando esses custos foram retirados da análise o VPL manteve-se positivo para a variação dos mesmos. Adicionalmente, ao retirar da análise os custos com a DRC e IC, foi possível verificar o valor de VPL negativo. Entretanto, o valor negativo foi menor que o investimento na ATF. Isso permitiu concluir que na ausência das categorias de custo mais influentes no VPL houve retorno de investimento, ainda que esse valor não tenha ultrapassado o valor de investimento na ATF, foi possível melhorar a relação custo-efetividade.
- As análises desenvolvidas neste estudo foram suficientes para a estruturação de uma ferramenta farmacoeconômica, que pode ser aplicável à implementação da ATF no SUS por auxiliar os gestores de saúde a planejar o investimento, calculando o impacto nos indicadores epidemiológicos e o retorno financeiro relativo ao valor que se tem para investir na ATF, assim, tal fato permitiu confirmar a hipótese deste estudo.

7. REFERÊNCIAS

AGUWA, C. N.; UKWE, C. V.; EKWUNIFE, O. I. Effect of pharmaceutical care programme on blood pressure and quality of life in a Nigerian pharmacy. **Pharmacy World & Science,** v. 30, n. 1, p. 107-110, 2008.

ALBANESI-FILHO, F. M. O que vem ocorrendo com a insuficiência cardíaca no Brasil? **Arquivos Brasileiros de Cardiologia,** v. 85, n. 3, p. 155-6, 2005.

ALBUQUERQUE, C.; PIOVESAN, M. F.; SANTOS, I. S.; MARTINS, A. C. M.; FONSECA, A. L.; SASSON, D.; SIMÕES, K. D. A. The current situation of the private health plans and insurance market in Brazil and trends for the future. **Ciência e Saúde Coletiva**, v. 13, n. 5, p. 1421-1430, 2008.

ALLEN, K.; HAZELETT, S.; JARJOURA, D.; HUA, K.; WRIGHT, K.; WEINHARDT, J.; et al. A randomized trial testing the superiority of a postdischarge care management model for stroke survivors. **Journal of Stroke and Cerebrovascular Diseases**, v. 18, n. 6, p. 443-52, 2009.

ALMEIDA, C. M. D. As reformas sanitárias dos anos 80: crise ou transição? **Cadernos de Saúde Pública**, v. 11, n. 4, p. 639-642, 1995.

AMERICAN ASSOCIATION OF CLINICAL ENDOCRINOLOGISTS – AACE. JELLINGER, P. S.; SMITH, D. A.; MEHTA, A. E.; GANDA, O.; HANDELSMAN, Y.; RODBARD, H. W.; et al. American Association of Clinical Endocrinologists' guidelines for management of dyslipidemia and prevention of atherosclerosis. **Endocrine Practice**, v. 18, Suppl. 1, p. 1-78, 2012.

ANTMAN, E. M.; SELWYN, A. P.; LOSCALZO, J. **Ischemic Heart Disease.** Chapter 243 *In: Harrison's Principles of Internal Medicine*. 18 ed. USA: The McGraw-Hill Companies, 2013.

ARAÚJO, A. L. A.; UETA, J. M.; FREITAS, O**.** Assistência Farmacêutica como um modelo tecnológico em atenção primária à saúde. **Revista de Ciências Farmacêuticas Básica e Aplicada**, v. 26, n. 2, p. 87-92, 2005.

ARAÚJO, D. V.; OLIVEIRA-JUNIOR, M. T. Análise de custo-efetividade do monitoramento da insuficiência cardíaca pelo proBNP comparado ao monitoramento clínico. **REBLAMPA Revista Brasileira Latino-americana de Marcapasso e Arritmia**, v. 20, n. 1, p. 18-22, 2007.

ARAÚJO, D. V.; TEICH, V.; PASSOS, R. B. F.; MARTINS, S. C. O. Analysis of the cost-effectiveness of thrombolysis with alteplase in stroke. **Arquivos Brasileiros de Cardiologia**, v. 95, n. 1, p. 12-20, 2010.

BARR, P. D. Hazards of modern diagnosis and therapy – the price we pay. **Journal of the American Medical Association**, v. 159, n. 15, p. 1452-6, 1955.

BARRETO, A. D. Intravenous thrombolytics for ischemic stroke. **Neurotherapeutics**, v. 8, n. 3, p. 388-99, 2011.

BASTOS, M. G.; KIRSZTAJN, G. M. Doença renal crônica: importância do diagnóstico precoce, encaminhamento imediato e abordagem interdisciplinar estruturada para melhora do desfecho em pacientes ainda não submetidos à diálise. **Jornal Brasileiro de Nefrologia**, v. 33, n. 1, p. 93-10, 2011.

BATISTA, L. K.; PINHEIRO, H. S. P.; FUCHS, R.; OLIVEIRA, T.; BELCHIOR, F. J.; GALIL, A. G.; et al. Manuseio da doença renal crônica em pacientes com hipertensão e diabetes. **Jornal Brasileiro de Nefrologia**, v. 27, n. 1, p. 8-13, 2005.

BOCCHI, E. A.; MARCONDES-BRAGA, F. G.; AYUB-FERREIRA, S. M.; ROHDE, L. E.; OLIVEIRA, W. A.; ALMEIDA, D. R.; et al. Sociedade Brasileira de Cardiologia. III diretriz Brasileira de Insuficiência Cardíaca Crônica. **Arquivos Brasileiros de Cardiologia**, v. 93, nº 1, supl. 1, p. 1-71, 2009.

BOCCHI, E. A.; VILAS-BOAS, F.; PERRONE, S.; CAAMAÑO, A. G.; CLAUSELL, N.; MOREIRA, M. C. V.; et al. I Diretriz Latino-Americana para avaliação e conduta na insuficiência cardíaca descompensada. **Arquivos Brasileiros de Cardiologia**, v. 85, supl. 3, p. 1-48, 2005.

BORGES, A. P. S. **Avaliação do impacto clínico e da viabilidade econômica da implantação de um modelo de atenção farmacêutica em uma UBDS de Ribeirão Preto-SP**. 2008. 119f. Dissertação (Mestrado em Medicamentos e Cosméticos). Faculdade de Ciências Farmacêuticas de Ribeirão Preto, Universidade de São Paulo, Ribeirão Preto, 2008.

BORGES, A. P. S.; GUIDONI, C. M.; FREITAS, O.; PEREIRA, L. R. L. Economic evaluation of outpatients with type 2 diabetes mellitus assisted by a pharmaceutical care service. **Arquivos Brasileiros de Endocrinologia & Metabologia,** v. 55, n. 9, p. 689-91, 2011.

BORTOLOTTO, L. A. Hipertensão arterial e insuficiência renal crônica. **Revista Brasileira de Hipertensão**, v. 15, n. 3, p. 152-5, 2008.

BRASIL. (1971). **Ministério da Saúde. Decreto n. 68.806, de 25 de junho de 1971. Institui a Central de Medicamentos (Ceme)**. Diário Oficial da União. Brasília, DF. 1971. Disponível em: < http://www.planalto.gov.br/ccivil_03/decreto/1970-1979/D68806.htm>. Acesso em: 25 mai 2015.

BRASIL. (1988). **Constituição da República Federativa do Brasil de 05 de outubro de 1988**. Brasília, DF. 1988. Disponível em: <http://www.senado.gov.br/legislacao/const/con1988/CON1988_05.10.1988/CON1988.shtm>. Acesso em: 26 mai 2015.

BRASIL. (1990). **Lei Complementar da Saúde. Lei nº 8142 de 28 de dezembro de 1990.** Dispõe sobre a participação da comunidade na gestão do Sistema Único de Saúde (SUS) e sobre as transferências intergovernamentais de recursos financeiros na área da saúde e dá outras providências. Brasília, DF. 1990. Disponível em: <http://www.planalto.gov.br/ccivil_03/leis/L8142.htm>. Acesso em: 22 mai 2015.

BRASIL. (1990). **Lei Orgânica da Saúde**. **Lei nº 8080, de 19 de setembro de 1990**. Dispõe sobre as condições para a promoção, proteção e recuperação da saúde, a organização e o funcionamento dos serviços correspondentes e dá outras providências. Diário Oficial da União, Brasília, DF. 1990. Disponível em: < http://www.planalto.gov.br/ccivil_03/Leis/L8080.htm>. Acesso em: 18 set 2015.

BRASIL. (1991). **Lei Orgânica da Seguridade Social. Lei nº 8.212 de 24 de julho de 1991.** Dispõe sobre a organização da seguridade social, institui o plano de custeio, e dá outras providências. Brasília, DF. 1991. Disponível em: < http://www.planalto.gov.br/ccivil_03/leis/l8213cons.htm>. Acesso em: 07 out 2015.

BRASIL. (1993). **Lei Orgânica da Assistência Social. Lei nº 8742 de 07 de dezembro de 1993.** Dispõe sobre a organização da Assistência Social e dá outras providências. Brasília, DF. 1993. Disponível em: <http://www3.dataprev.gov.br/ sislex/paginas/42/1993/8742.htm>. Acesso em: 15 mai 2015.

BRASIL. (1998). Ministério da Saúde. **Portaria GM nº 3.916, de 30 de outubro de 1998. Aprova a Política Nacional de Medicamentos** [Internet]. Brasília, DF. 1998. Disponível em: <http://www.anvisa.gov.br/legis/consolidada/portaria_3916_98.pdf>. Acesso em: 21 jun 2015.

BRASIL. (2000). **Emenda Constitucional nº 29 de 13 de setembro de 2000.** Dispõe sobre recursos mínimos para o financiamento das ações e serviços públicos de saúde. Brasília, DF. 2000. Disponível em: <http://www.planalto.gov.br/ccivil_03/constituicao/emendas/emc/emc29.htm>. Acesso em: 14 set 2015.

BRASIL. (2002). Ministério da Saúde. Secretaria de Políticas de Saúde. Departamento de Atenção Básica. **Política Nacional de Medicamentos**. Brasília, DF. 2002. Disponível em: <http://bvsms.saude.gov.br/bvs/publicacoes/politica_medicamentos.pdf>. Acesso em: 20 junho 2015.

BRASIL. (2004). Ministério da Saúde. Conselho Nacional de Saúde. **Resolução n° 338 de 06 de maio de 2004. Aprova a Política Nacional de Assistência Farmacêutica**. Brasília, DF. 2004. Disponível em: <http://bvsms.saude.gov.br/bvs/saudelegis/cns/2004/res0338_06_05_2004.html>. Acesso em: 20 junho 2015.

BRASIL**.** (2006). Ministério do Trabalho e Emprego. **Sistema de informações para acompanhamento das negociações coletivas no Brasil. Encargos sociais no Brasil: conceito, magnitude e reflexos no emprego**. *[online]*. Brasília, DF. 2006. Disponível em: <http://portal.mte.gov.br/data/files/FF8080812BA5F4B7012BAB0ABAFF6414/Prod04_2006.pdf>. Acesso em: 12 fev 2015.

BRASIL. (2007). **Conselho Nacional de Secretários de Saúde. Assistência Farmacêutica no SUS / Conselho Nacional de Secretários de Saúde**. Brasília, DF: CONASS, 2007. 186p.

BRASIL. (2008). IBGE - Instituto Brasileiro de Geografia e Estatística. **Pesquisa mensal de emprego. Trabalhadores por conta própria: perfil e destaques**. Disponível em: <http://www.ibge.gov.br/home/estatistica/indicadores/trabalhoerendimento/pme_nova/parte1.pdf >. Acesso em: 02 set 2015.

BRASIL. (2008). Ministério da Saúde. Secretaria de Vigilância em Saúde. Secretaria de Atenção à Saúde. **Diretrizes e recomendações para o cuidado integral de doenças crônicas não-transmissíveis: promoção da saúde, vigilância, prevenção e assistência.** Brasília, DF. 2008. 72p.

BRASIL. (2009). Ministério da Saúde. Secretaria-Executiva. Subsecretaria de Planejamento e Orçamento. **Sistema de planejamento do SUS: uma construção coletiva: instrumentos básicos / Ministério da Saúde, Secretaria-Executiva, Subsecretaria de Planejamento e Orçamento**. 2 ed. Brasília, DF. 2009. 56p.

BRASIL**.** (2009). Ministério da Saúde. Rede Brasileira de Avaliação de Tecnologia em Saúde (REBRATS). **Diretrizes metodológicas, estudos de avaliação econômica de tecnologias em saúde**. Série A. Normas e Manuais Técnicos. *[online]*. 1 ed. Brasília, DF. 2009. 145p.

BRASIL. (2010). Ministério da Saúde. Secretaria Executiva. **DATASUS: Informações de Saúde. Estatísticas vitais.** 2010. Disponível em: < http://www2.datasus.gov.br/DATASUS/index.php?area=0205>. Acesso em: 27 nov 2015.

BRASIL. (2011). **Conselho Nacional de Secretários de Saúde. Sistema Único de Saúde / Conselho Nacional de Secretários de Saúde**. Brasília, DF. 2011. 291 p.

BRASIL**.** (2012a). Ministério da Saúde. Rede Intergerencial de Informações para a Saúde. **Indicadores e Dados Básicos - Brasil – 2012**. *[online]*. Disponível em:< http://tabnet.datasus.gov.br/cgi/idb2012/matriz.htm>. Acesso em: 06 abr 2015.

BRASIL. (2012b). IBGE - Instituto Brasileiro de Geografia e Estatística. **Cidades. Ribeirão Preto**. Disponível em: <http://www.cidades.ibge.gov.br/xtras/temas.php?lang=&codmun=354340&idtema=16&search=%7C%7Cs%EDntese-das-informa%E7%F5es>. Acesso em: 02 mar 2015.

BRASIL. (2013a). IBGE - Instituto Brasileiro de Geografia e Estatística. **Pesquisa nacional de saúde. Percepção do estado de saúde, estilos de vida e doença crônica**. IBGE, 2013. Disponível em: <ftp://ftp.ibge.gov.br/PNS/2013/pns2013.pdf>. Acesso em: 02 jul 2014.

BRASIL**.** (2013b). BCB - Banco Central do Brasil. **Indicadores econômicos consolidados** *[online]*. Disponível em: <http://www.bcb.gov.br/?INDECO>. Acesso em: 10 mai 2013.

BRASIL. (2014). Ministério da Saúde. Secretaria de Atenção à Saúde. Departamento de Atenção Especializada e Temática. **Diretrizes Clínicas para o cuidado ao paciente com Doença Renal Crônica – DRC no Sistema Único de Saúde/Ministério da Saúde**. Brasília, DF. 2014.

BRASIL**.** (2014a). **Medida Provisória nº 664 de 30 de dezembro de 2014**. Brasília, DF. 2014. Disponível em: <http://www.planalto.gov.br/ccivil_03/_Ato2011-2014/2014/Mpv/mpv664.htm>. Acesso em: 15 fev 2015.

BRASIL**.** (2014b). **IBGE - Instituto Brasileiro de Geografia e Estatística.** *[online]*. Disponível em: <http://www.ibge.gov.br/home/>. Acesso em: 25 nov 2014.

BRASIL. (2014c). Ministério da Saúde. Secretaria de Ciência, Tecnologia e Insumos Estratégicos. Departamento de Assistência Farmacêutica e Insumos Estratégicos. **Cuidado Farmacêutico na Atenção Básica. Caderno 1: Serviços Farmacêuticos na Atenção Básica**. Brasília, DF. 2014.

BRASIL**.** (2015a). PMRP - Prefeitura Municipal de Ribeirão Preto. TRANSERP - Empresa de Transporte Urbano de Ribeirão Preto. **Tarifas no transporte público de Ribeirão Preto.** *[online]*. Disponível em: <https://www.ribeiraopreto.sp.gov.br/transerp/i07tarifas-trans.php>. Acesso em: 21 mar 2015.

BRASIL. (2015b). Ministério da Saúde. **Sistema de gerenciamento da tabela de procedimentos, medicamentos e OPM do SUS**. *[online]*. Disponível em: <http://sigtap.datasus.gov.br/tabela-unificada/app/sec/inicio.jsp>. Acesso em: 21 fev 2015.

BRASIL. (2015c). Prefeitura Municipal de Ribeirão Preto. **Secretaria de Saúde do município de Ribeirão Preto**. 2015. Disponível em: < http://www.ribeiraopreto.sp.gov.br/ssaude/i16saude.php>. Acesso em: 23 mar 2014.

BRASIL. (2015d). IBGE - Instituto Brasileiro de Geografia e Estatística. **Pesquisa Mensal de Emprego**. Disponível em: <http://www.ibge.gov.br/home/estatistica/indicadores/trabalhoerendimento/pme_nova/defaulttab_hist.shtm.>. Acesso em: 15 mar 2015.

BRASIL. (2015e). Ministério da Previdência Social. **Seguridade Social**. *[online]*. Disponível em: < http://www4.seg-social.pt/>. Acesso em: 13 fev 2015.

BRASIL. (2015f). Ministério da Fazenda. Receita Federal do Brasil. **Formas de contribuição.** *[online]*. Disponível em: <http://www.receita.fazenda.gov.br/previdencia/formascontrib.htm#Alíquotas>. Acesso em: 15 mar 2015.

BRASIL. (2015g). Ministério da Saúde. DATASUS. **Departamento de Informática do DATASUS.** *[online]*. Disponível em: <http://www2.datasus.gov.br/DATASUS/index.php?area=01> Acesso em: 11 mai 2015.

BRASIL. (2015h). Ministério da Previdência Social. **Pensão por morte**. Disponível em: <http://www.previdencia.gov.br/servicos-ao-cidadao/todos-os-servicos/pensao-por-morte/>. Acesso em: 15 mar 2015.

BRASIL. (2015i). IBGE - Instituto Brasileiro de Geografia e Estatística. **Pesquisa mensal de emprego. Rendimento médio real**. Disponível em: <http://www.ibge.gov.br/home/estatistica/indicadores/trabalhoerendimento/pme_nova/pme_201509sp_02.shtm>. Acesso em: 16 out 2015.

BRASIL. (2015j). Ministério do Trabalho e Emprego. **Trabalhador. Salário mínimo**. Disponível em: <http://www.mte.gov.br/index.php/salario-minimo>. Acesso em: 16 mar 2015.

BRASIL. (2015l). Prefeitura Municipal de Ribeirão Preto**. Prestação de contas de 2013**. Disponível em: <http://www.ribeiraopreto.sp.gov.br/ssaude/principal/prestacao/prest-3-quadrimestre-2013.pdf.>. Acesso em: 15 out 2015.

BRASIL. (2015m). Prefeitura Municipal de Ribeirão Preto**.** Secretaria de saúde de Ribeirão Preto. **Relatório de gestão de 2013.** Disponível em: <https://www.ribeiraopreto.sp.gov.br/ssaude/vigilancia/planeja/2013relatorio_gestao.pdf.>. Acesso em: 15 out 2015.

BRASIL. (2015n). Prefeitura Municipal de Ribeirão Preto**.** Secretaria de saúde de Ribeirão Preto. **Plano municipal de saúde 2014-2017.** Disponível em: <https://www.ribeiraopreto.sp.gov.br/ssaude/vigilancia/planeja/pms-rp-2014-2017.pdf.>. Acesso em: 15 out 2015.

BRASIL. (2015o). Prefeitura Municipal de Ribeirão Preto**.** Secretaria de saúde de Ribeirão Preto. **Programas/serviços.** Disponível em: <http://www.ribeiraopreto.sp.gov.br/ssaude/programas/cronico/chd-epidemiologia-desafios.pdf.>. Acesso em: 15 out 2015.

BRASIL. (2015p). IBGE - Instituto Brasileiro de Geografia e Estatística. **Brasil em síntese. PIB per capta nacional**. Disponível em: <http://brasilemsintese.ibge.gov.br/contas-nacionais/pib-per-capita.html>. Acesso em: 16 mar 2015.

BRASIL. (2015q). Portal Brasil. **Economia e emprego**. Disponível em: < http://www.brasil.gov.br/economia-e-emprego/2015/11/governo-eleva-taxa-de-retorno-para-projetos-em-ferrovias>. Acesso em: 12 nov 2015.

BRASIL. (2015r). Agência Brasil. **Economia: cai a taxa de retorno dos investimentos em imóveis comerciais**. Disponível em: <http://agenciabrasil.ebc.com.br/economia/noticia/2015-11/cai-taxa-de-retorno-dos-investimentos-em-imoveis-comerciais>. Acesso em: 12 nov 2015.

CALVET, X.; RUÍZ, M. A.; DOSAL, A.; MORENO, L.; LÓPEZ, M.; FIGUEROLA, A.; et al. Cost-minimization analysis favours intravenous ferric carboxymaltose over ferric sucrose for the ambulatory treatment of severe iron deficiency. **PLOS ONE**, v. 7, n. 9, p. e45604, 2012.

CARTER, B. L.; ARDERY, G.; DAWSON, J. D.; JAMES, P. A.; BERGUS, G. R.; DOUCETTE, W. R.; et al. Physician and pharmacist collaboration to improve blood pressure control. **Archives of Internal Medicine,** v. 169, n. 21, p. 1996-2002, 2009a.

CARTER, B. L.; ROGERS, M.; DALY, J.; ZHENG, S.; JAMES, P. A. The potency of team-based care interventions for hypertension: a meta-analysis. **Archives of Internal Medicine,** v. 169, n.19, p. 1748-55, 2009b.

CICONELLI, R. M.; FERRAZ, M. B.; SANTOS, W.; MEINÃO, I.; QUARESMA, M. R.. Tradução para a língua portuguesa e validação do questionário genérico de avaliação da

qualidade de vida SF-36. (Brasil SF-36). **Revista Brasielira de Reumatologia**, v. 39, n. 3, p. 143-150, 2004.

CHEN, S. C. C.; TOY, M.; YEH, J. M.; WANG, J. D.; RESCH, S. Cost-effectiveness of Augmenting Universal Hepatitis B Vaccination With Immunoglobin Treatment. **Pediatrics**, v. 131, n. 4, p. 1135-1143, 2013.

CHIEVINATO, I. **Recursos humanos: o capital humano das organizações**. 8. ed. São Paulo: Atlas, 2004. 522p.

CIPOLLE, R. J; STRAND, L. M; MORLEY, P. C. **Pharmaceutical Care Practice**. New York: McGraw-Hill, 1998. 359p.

CIPOLLE, R.J.; STRAND, L.M.; MORLEY, P.C. **O exercício do cuidado farmacêutico**. Trad. Denise Borges Bittar, Brasília: Conselho Federal de Farmácia, 2006. 396p.

CIPULLO, J. P.; MARTIN, J. F.; CIORLIA, L. A.; GODOY, M. R.; CAÇÃO, J. C.; LOUREIRO, A. A. et al. Hypertension prevalence and risk factors in a Brazilian urban population. **Arquivos Brasileiros de Cardiologia,** v. 94, n. 4, p. 519-26, 2010.

COUTINHO, E. S. F.; CUNHA, G. M. Basic concepts in epidemiology and statistics for reading controlled clinical trials. **Revista Brasileira de Psiquiatria,** v. 27, n. 2, p. 146-51, 2005.

DAWBER, T. R.; MOORE-Jr., F. E.; MANN, G. V. Coronary Heart Disease in the Framingham Study. **American Journal of Public Health and the Nation's Health**, v.47, p. 4–24, 1957.

DE CASTRO, M. S.; CORRER, C. J. Pharmaceutical care in community pharmacies: practice and research in Brazil. **The Annals of Pharmacotherapy,** v. 41, n. 9, p. 1486-1493, 2007.

DIB, M. W.; RIERA, R.; FERRAZ, M. B. Estimated annual cost of arterial hypertension treatment in Brazil. **Pan American Journal of Public Health,** v. 27, n. 2, p. 125-31, 2010.

DOMINGO, J. D.; RIDAO, M.; LATOUR, J.; BALLESTER, A.; MORANT, A. A cost benefit analysis of routine varicella vaccination in Spain. **Vaccine**. v. 17, n. 11, p. 1306-11. 1999.

DRUMMOND, F. M.; SCULPHER, M. J.; TORRANCE, G. W.; O'BRIEN, B. J.; STODDART, G. L. **Methods for the economic evaluation of health care programmes**. 3. ed. Oxford: Oxford University press, 2005. 379p.

EIZERIK, D. P.; MANFROI, W. C. Efficacy of Pharmaceutical Care in dyslipidemia: systematic review. **Revista do Hospital das Clínicas de Porto Alegre,** v. 28, n. 1, p. 37-40, 2008.

ETEMAD, L. R.; HAY, J. W. Cost-Effectiveness Analysis of Pharmaceutical Care in a Medicare Drug Benefit Program. **Value in Health**, v. 6, n. 4, p. 425-435, 2003.

FERREIRA, L. N.; COSTA, M. C.; SOUSA, J. T. Perfil sociodemográfico e variáveis clínicas dos Hipertensos atendidos na clinica escola de fisioterapia da UESB. **The Journal of Health.com.**, v. 7, n.1, p. 23-32, 2011.

FINKELMAN, J. **Caminhos da saúde pública no Brasil** *[online]*. Rio de Janeiro: FIOCRUZ, 2002. Disponível em: < http://www.arca.fiocruz.br/bitstream/icict/2705/1/Finkelman_Jacobo(Org.).pdf >. Acesso em: 15 nov 2014.

FLEURENCE, R. L.; HOLLENBEAK, C. S. Rates and probabilities in economic modelling. **PharmacoEconomics,** v. 25, n. 1, p. 3-6, 2007.

FLEURY S.; OUVERNEY A. M. Política de Saúde: uma política social. **Políticas e Sistema de Saúde no Brasil**. Rio de Janeiro: Editora Fiocruz. 2008. p. 23-64.

FRANCO, L. J.; PASSOS, A. D. C. **Fundamentos de epidemiologia**. 1 ed. Barueri, SP: Manole, 2005. 380p.

GARCIA, L. A. Epidemiology and pathophysiology of lower extremity peripheral arterial disease. **Journal of Endovascular Therapy**, v. 13, supl. 2, p. II3-9, 2006.

GARRITANO, C. R.; LUZ, P. M.; PIRES, M. L. E.; BARBOSA, M. T. S.; BATISTA, K. M. Análise da tendência da mortalidade por acidente vascular cerebral no Brasil no século XXI. **Arquivos Brasileiros de Cardiologia**, v. 98, n. 6, p. 519-27, 2012.

GIROLINETO, B. M. P. **Desenvolvimento e avaliação do INSAF-HAS: um formulário de seleção de pacientes para inserção em um serviço de atenção farmacêutica**. 2015. 134f. Tese (doutorado em medicamentos e cosméticos). Faculdade de Ciências Farmacêuticas de Ribeirão Preto – Universidade de São Paulo, Ribeirão Preto, 2015.

GISMONDI, R. A. O. C.; MARTINO, H.; SOARES, R. C.; GAZE, M. S. V.; CUNHA, A. B.; ROCHA, A. S. C. Doença Arterial Coronariana e Mixoma Cardíaco: prevalência e associação

com os fatores de risco convencionais. **Revista da Sociedade de Cardiologia do Estado do Rio de Janeiro,** v. 20, n. 1, p. 16-19, 2007.

GROVER, S.; COUPAL, L.; LOWENSTEYN, I. Preventing cardiovascular disease among Canadians: is the treatment of hypertension or dyslipidemia cost-effective?. **Canadian Journal of Cardiology**, v. 24, n.12, p. 891-898, 2008.

HANKEY, G. J.; NORMAN, P. E.; EIKELBOOM, J. W. Medical treatment of peripheral arterial disease. **The Journal of the American Medical Association - JAMA**, v. 295, n. 5, p. 547-53, 2006.

HANRATTY, R.; CHONCHOL, M.; HAVRANEK, E. P.; POWERS, J. D.; DICKINSON, L. M.; HO, P. M., et al. Relationship between blood pressure and incident chronic kidney disease in hypertensive patients. **Clinical Journal of the American Society of Nephrology**, v. 6, n. 11, p. 2605-2611, 2011.

HEPLER, C. D.; STRAND, L. M. Opportunities and responsibilities in pharmaceutical care. **The American Journal of Pharmacy Education**, v. 53, p.75-155, 1990.

HERNANDO, F. J. S.; CONEJERO, A. M. Peripheral artery disease: pathophysiology, diagnosis and treatment. **Revista Espanola de Cardiologia**, v. 60, n. 09, p. 969-82, 2007.

INTERNATIONAL SOCIETY FOR PHARMACOECONOMICS AND OUTCOMES RESEARCH – ISPOR. 2015. **Publications data base**. *[on line]*. Disponível em: <http://www.ispor.org/publications/index.asp>. Acesso em: 02 dez 2015.

JARDIM, P. C. V.; GONDIM, M. R. P.; MONEGO, E. T.; MOREIRA, H. G.; VITORINO, P. V. O.; SOUZA, W. K. S.; SCALA, L. C. N. Hipertensão arterial e alguns fatores de risco em uma capital brasileira. **Arquivos Brasileiros de Cardiologia,** v. 88, n. 4, p. 452-7, 2007.

KLAHR, S.; LEVEY, A. S.; BECK, G. J.; CAGGIULA, A. W.; HUNSICKER, L.; KUSEK, J. W.; STRIKER, G. The effects of dietary protein restriction and blood pressure control on the progression of chronic renal disease. **The New England Journal of Medicine**, v. 330, n. 13, p. 877-84, 1994.

KULASINGAM, S. L.; EVAN, R. M. "Potential health and economic impact of adding a human papillomavirus vaccine to screening programs." **JAMA.** v. 290, n. 6, p. 781-789, 2003.

LAURENTI, R.; BUCHALLA, C. M.; CARATIN, V. S. Ischemic heart disease. Hospitalization, length of stay and expenses in Brazil from 1993 to 1997. **Arquivos Brasileiros de Cardiologia,** v.74, n.6, p.483-92, 2000.

LENFANT, C.; CHOBANIAN, A.V.; JONES, D.W.; ROCCELLA, E.J. Seventh report of the Joint National Committee on the prevention, detection, evaluation, and treatment of high blood pressure (JNC 7): resetting the hypertension sails. **Hypertension**, v. 41, n. 6, p. 1178-79, 2003.

LENFANT, C.; ROCCELLA, E. J. A call to action for more aggressive treatment of hypertension. **Journal of Hypertension.**, v. 17, n. 1, p. S3-S17, 1999.

LEVY, D.; LARSON, M. G.; VASAN, R. S.; KANNEL, W. B.; HO, K. K. The progression from hypertension to congestive heart failure. **The Journal of the American Medical Association - JAMA,** v. 275, n. 20, p. 1557- 62, 1996.

LOTUFO, P. A. O escore de risco Framingham para doenças cardiovasculares. **Revista Médica**, v. 87, n. 4, p. 232-7, 2008.

LOWEY, A.; MOORE, S.; NORRIS, C.; WRIGHT, D.; SILCOCK, J.; HAMMOND, P. The cost-effectiveness of pharmacist-led treatment of cardiac risk in patients with type 2 diabetes. **Pharmacy World & Science**, v. 29, n. 5, p. 541–545, 2007.

LUNA, R. L. **Hipertensão arterial: diagnóstico e tratamento**. 2. ed. Rio de Janeiro: Revinter, 2009. 252p.

LYRA-JUNIOR, D. P.; MARCELLINI, P. S.; PELÁ, I. R. Effect of pharmaceutical care intervention on blood pressure of elderly outpatients with hypertension. **Brazilian Journal of Pharmaceutical Sciences**, v.44, n. 3, p. 451-457, 2008.

MACHADO, M; BAJCAR, J; GUZZO, G. C.; EINARSON, T. R. Sensitivity of patient outcomes to pharmacist interventions. Part II: Systematic review and meta-analysis in hypertension management. **The Annals of Pharmacotherapy,** v. 41, n. 11, p. 1170-1181, 2007.

MAGNANINI, M. M. F.; NOGUEIRA, A. R.; CARVALH, M. S.; BLOCH, K. V. Monitorização ambulatorial da pressão arterial e risco cardiovascular em mulheres com hipertensão resistente. **Arquivos Brasileiros de Cardiologia**, v. 92, n. 6, p. 484-489, 2009.

MANSUR, A. D. P.; FAVARATO, D. Mortalidade por doenças cardiovasculares no Brasil e na região metropolitana de São Paulo: atualização 2011. **Arquivos Brasileiros de Cardiologia**, v. 99, n. 2, p. 755-761, 2012.

McCULLAGH, P; NELDER, J. A. **Monographs on Statistics & Applied Probability: Generalized Linear Models**. 2 ed. Florida-USA: CRC PRESS, 1989. 532p.

MESQUITA, E. T.; SOCRATES, J.; RASSI, S.; VILLACORTA, H.; MADY, C. Insuficiência cardíaca com função sistólica preservada. **Arquivos Brasileiros de Cardiologia,** v. 82, n. 5, p. 494-500, 2004.

MILLER, D. K.; HOMAN, S. M. Determining Transition Probabilities Confusion and Suggestions. **Medical Decision Making.**, v. 14, n. 1, p. 52-58, 1994.

MORAES, S. A.; FREITAS, I. C. M. Doença isquêmica do coração e fatores associados em adultos de Ribeirão Preto, SP. **Revista de Saúde Pública**, v. 46, n.4, p.591-601, 2012.

MOTA, D. M. Avaliação Farmacoeconômica: Instrumentos de medida dos benefícios na Atenção Farmacêutica. **Acta Farmacêutica Bonaerense**, v. 22, n. 1, p. 73-80, 2003.

MUNTNER, P.; SAFFORD, M. M.; CUSHMAN, M.; HOWARD, G. Comment on the reports of over-estimation of ASCVD risk using the 2013 AHA/ACC risk equation. **Circulation**, v. 129, n. 2, p. 266-267, 2014.

MURABITO, J. M. Women and cardiovascular disease: contributions from the Framingham Heart Study. **Journal of the American Medical Women's Association**, v.50, n. 2, p.35-9. 1995.

NASCIMENTO, J. S.; GOMES, B.; SARDINHA, A. H. L. Modifiable risk factors for cardiovascular diseases in women with hypertension. **Revista da Rede de Enfermagem do Nordeste - RENE**, v. 12, n. 4, p. 709-15, 2011.

NETO, A. A.; LIMA, F. G. **Curso de administração financeira: Manual do Mestre**. 1. ed. São Paulo: Atlas, 2009. 840p.

NEYELOFF, J. L. **Rastreio de doença arterial obstrutiva periférica: impacto no tratamento farmacológico de pacientes hipertensos e análise de custo-efetividade para re-estratificação de risco cardiovascular**. 2012. 126f. Tese (doutorado) - Faculdade de Medicina da Universidade Federal do Rio Grande do Sul. Porto Alegre. 2012.

NITA, M. E.; SECOLI, S. R.; NOBRE, M. R. C.; ONO-NITA, S. K.; CAMPINO, A. C. C.; SARTI, F. M.; et al. **Avaliação de tecnologias em saúde: evidência clínica, análise econômica e análise de decisão.** Porto Alegre: Artmed, 2009. 600p.

NORGREN, L.; HIATT, W. R.; DORMANDY, J. A.; NEHLER, M. R.; HARRIS, K. A.; FOWKES, F. G.; TASC II Working Group. Inter-society consensus for the management of peripheral arterial disease (TASC II). **Journal of Vascular Surgery,** v. 45, suppl. S, p. S5-67, 2007.

OLIVEIRA, C. M. X. **Modelo de intervenções educativas para o uso racional de medicamentos**. 2013. 220f. Tese (Doutorado) - Faculdade de Ciências Farmacêuticas de Ribeirão Preto – Universidade de São Paulo, Ribeirão Preto. 2013.

OLIVEIRA, M. A.; BERMUDEZ, J. A. Z.; OSÓRIO-DE-CASTRO, C. G. S. **Assistência Farmacêutica e acesso a medicamentos.** Rio de Janeiro: SciELO-Editora FIOCRUZ, 2007. 112p.

OLIVEIRA, P. A. R.; MENEZES, F. G. Atenção Farmacêutica a pacientes hipertensos. **Revista Eletrônica de Farmácia**, v. 10, n. 1, p. 51-68, 2013.

ORGANIZAÇÃO MUNDIAL DA SAÚDE - OMS. (2003) **Cuidados inovadores para as condições crônicas: componentes estruturais de ação**. Relatório mundial. Brasília, DF. 2003.

ORGANIZAÇÃO MUNDIAL DE SAÚDE – OMS. (2004). **Classificação internacional de funcionalidade, incapacidade e saúde - CIF: classificação detalhada com definições.** 2004. Disponível em: < http://www.inr.pt/uploads/docs/cif/CIF_port_%202004.pdf >. Acesso em: 25 nov 2015.

ORGANIZAÇÃO PAN-AMERICANA DE SAÚDE - OPAS. (2002). **Consenso brasileiro de Atenção Farmacêutica: proposta**. Brasília, DF: Organização Pan-Americana da Saúde, 24 p.

PAGANO, M.; GAUVREAU, M. **Princípios de Bioestatística**. 2. ed. São Paulo: Cengage Learning, 2012. 506p.

PENAFORTE, T. R. **Atenção Farmacêutica na hipertensão arterial sistêmica, impacto clínico, econômico e social.** 2011. 189f. Tese (Doutorado) – Faculdade de Ciências Farmacêuticas da Universidade de São Paulo, Ribeirão Preto –SP. 2011.

PEREIRA, L. R. L.; FREITAS, O. D. A evolução da Atenção Farmacêutica e a perspectiva para o Brasil. **Revista Brasileira de Ciências Farmacêuticas,** v. 44, n. 4, p. 601-12, 2008.

PICCINATO, C. E.; CHERRI, J.; MORIYA, T. Hipertensão e doença arterial periférica. **Revista Brasileira de Hipertensão**, v. 8, n. 3, p. 306-15, 2001.

PIEGAS, L. S.; FEITOSA, G.; MATTOS, L. A.; NICOLAU, J. C.; ROSSI-NETO, J. M.; TIMERMAN, A.; et al; Sociedade Brasileira de Cardiologia. Diretriz da Sociedade Brasileira de Cardiologia sobre tratamento do infarto agudo do miocárdio com supradesnível do segmento ST. **Arquivos Brasileiros de Cardiologia,** v. 93, n. 6, supl. 2, p. e179-e264, 2009.

PIOLA, S. F.; VIANNA, S. M.; MARINHO, A.; CARVALHO, D.; RIBEIRO, J. A.; SILVA, J. R.; et al. Saúde no Brasil: algumas questões sobre o Sistema Único de Saúde (SUS). Comissão econômica para a América Latina e o Caribe - CEPAL/IPEA. [documento técnico]. **LC/BRS/R.200**, 2009. 77p.

PONTES-NETO, O. M.; SILVA, G. S.; FEITOSA, M. R.; DE FIGUEIREDO, N. L.; FIOROT J. A. Jr., ROCHA, T. N.; et al. Stroke awareness in Brazil: alarming results in a community-based study. **Stroke,** v. 39, n. 2, p. 292-6, 2008.

RASCATI, K. L. **Introdução à Farmacoeconomia**. 1. ed. Porto Alegre: Artmed, 2010. 280p.

REDDY, K. S.; YUSUF, S. Emerging epidemic of cardiovascular diseases in developing countries. **Circulation,** v.97, p.596-601, 1998.

REIS, T. M. D.; GUIDONI, C. M.; GIROTTO, E.; RASCADO, R. R.; MASTROIANNI, P. D. C.; CRUCIOL, J. M.; & PEREIRA, L. R. L. Knowledge and conduct of pharmacists for dispensing of drugs in community pharmacies: a cross-sectional study. **Brazilian Journal of Pharmaceutical Sciences**, v. 51, n. 3, p. 733-744, 2015.

ROMÃO JUNIOR, J. E. Chronic kidney disease: definition, epidemiology, and classification. **Jornal Brasileiro de Nefrologia**, v. 26, p. 1-3, 2004.

ROSAMOND, W.; FLEGAL, K.; FURIE, K.; GO, A.; GREENLUND, K.; HAASE, N.; et al. Heart disease and stroke statistics--2008 update: a report from the American Heart Association Statistics Committee and Stroke Statistics Subcommittee. **Circulation,** v. 117, n. 4, p. e-25-e146, 2008.

ROSÁRIO, T. M.; SCALA, L. C. N.; FRANÇA, G. V. A.; PEREIRA, M. R. G.; JARDIM, P. C. B. V. Prevalência, controle e tratamento da hipertensão arterial sistêmica em Nobres - MT. **Arquivos Brasileiros de Cardiologia,** v. 93, n. 6, p. 672-8, 2009.

ROUSH, G; FAGARD, R; SALLES, G; PIERDOMENICO, S.; REBOLDI, G.; VERDECCHIA, P.; et al. Prognostic impact of clinic, daytime, and nighttime systolic blood pressure in 9 cohorts of 13,843 patients with hypertension: systematic review and meta-analysis**. Journal of the American Society of Hypertension**, v.8, n. 4, p. 45-64, 2014.

SACCO, R. L.; WOLF, P. A.; GORELICK, P. B. Risk factors and their management for stroke prevention: outlook for 1999 and beyond. **Neurology,** v. 53, n. 7, supl. 4, p. S15-24, 1999.

SESSO, R.; LOPES, A. A.; THOMÉ, A. S.; BEVILACQUA, J. L.; ROMÃO-JUNIOR, J. E.; LUGON, J. Relatório do Censo Brasileiro de Diálise. **Jornal Brasileiro de Nefrologia**, v. 36, n. 1, p. 233-8, 2008.

SILVA-CASTRO, M. M.; VALLS, L. T.; FAUS, M. J. Revision sistemática sobre la implantación y la evaluación del seguimiento farmacoterapêutico em pacientes hospitalizados. **Farmacia Hospitalaria**, v. 34, n. 3, p. 106–124, 2010.

SIVIERO, P. C. L.; MACHADO, C. J.; CHERCHIGLIA, M. L. Insuficiência renal crônica no Brasil segundo enfoque de causas múltiplas de morte. **Caderno de Saúde Coletiva,** v. 22, n. 1, p. 75-85, 2014.

SOCIEDADE BRASILEIRA DE ANGIOLOGIA E CIRURGIA VASCULAR (SBACV). **Projeto Diretrizes: Doença Arterial Periférica Obstrutiva, Diagnóstico e Tratamento. 2014**. Disponível em: <http://sbacv.com.br/diretrizes/2015/DIRETRIZES-DAPO.pdf>. Acesso em: 29 nov 2015.

SOCIEDADE BRASILEIRA DE CARDIOLOGIA - SBC. VI Diretrizes Brasileiras de Hipertensão. **Arquivos Brasileiros de Cardiologia,** v. 95, n. 1, supl.1, p. 1-51, 2010.

SOUZA, W. A.; YUGAR-TOLEDO, J. C.; BERGSTEN-MENDES, G.; SABHA, M.; MORENO-JUNIOR, H. Effect of pharmaceutical care on blood pressure control and health-related quality of life in patients with resistant hypertension. **American Journal of Health-System Pharmacy**, v. 64, n. 18, p. 1955-61, 2007.

STELL, K.; GERTMAN, P. M.; CRESCENZI, C.; ANDERSON, J. Iatrogenic illness on a general medical service at a university hospital. **The New England Journal of Medicine**, v. 304, n. 11, p. 638-42, 1981.

STEVANOVIC, J.; POSTMA, M. J.; PECHLIVANOGLOU, P. A systematic review on the application of cardiovascular risk prediction models in pharmacoeconomics, with a focus on primary prevention. **European Journal of Preventive Cardiology,** v. 19, suppl. 2, p. 42–53, 2012.

STOKES, J. 3rd.; KANNEL, W. B.; WOLF, P. A.; D'AGOSTINO, R. B.; CUPPLES, L. A. Blood pressure and the risk for cardiovascular disease.The Framingham study – 30 years of follow up. **Hypertension**, v. 13, supl. 5, p. I13-8, 1989.

STONE, N. J.; ROBINSON, J.; LICHTENSTEIN, A. H.; BAIREY-MERZ, C. N.; BLUM, C. B.; ECKEL, R. H.; et al. 2013 ACC/AHA guideline on the treatment of blood cholesterol to reduce atherosclerotic cardiovascular risk in adults: a report of the American College of Cardiology/American Heart Association Task Force on Practice Guidelines. **Circulation,** v. 0, p. 000-000, 2013.

TEIXEIRA, H. V.; TEIXEIRA, M. G. Financiamento da saúde pública no Brasil: a experiência do Siops. **Revista Ciências e Saúde Coletiva.**, v. 8, n. 2, p. 379-391, 2003.

UNITED STATES RENAL DATA SYSTEM (USRDS). **Atlas of Chronic Kidney Disease and End-Stage Renal Disease in the United States**. National Institutes of Health, National Institute of Diabetes and Digestive and Kidney Diseases, Bethesda MD, 2009.

VALE, L.; STEFFENS, H.; DONALDSON, C. The costs and benefits of community thrombolysis for acute myocardial infarction: a decision-analytic model. **Pharmacoeconomics**, v. 22, n. 14, p. 943-954, 2004.

VON MUENSTER, S. J.; CARTER, B. L.; WEBER, C. A.; ERNST, M. E.; MILCHAK, J. L.; STEFFENSMEIER, J. J. G.; XU, Y. Description of pharmacist interventions during physician-pharmacist co-management of hypertension. **Pharmacy World & Science**, v. 30, n. 1, p. 128-35, 2008.

VONKEMAN, H. E.; BRAAKMAN-JANSEN, L. M.; KLOK, R. M.; POSTMA, M. J.; BROUWERS, J. R.; VAN DE LAAR, M. A. F. J. Incremental cost effectiveness of proton pump inhibitors for the prevention of non-steroidal anti-inflammatory drug ulcers: a pharmacoeconomic analysis linked to a case-control study. **Arthritis Research & Therapy**, v. 10, n. 6, p. 1-7, 2008.

WORLD HEALTH ORGANIZATION - WHO. (2011). **Global status report on noncommunicable diseases, 2010.** *[online]*. Disponível em: <http://www.who.int/nmh/publications/ncd_report_full_en.pdf>. Acesso em: 12 dez 2014.

WORLD HEALTH ORGANIZATION - WHO**.** (2013). **Media centre. Cardiovascular Diseases (CVDs).** *[online]*. Disponível em: <http://www.who.int/mediacentre/factsheets/fs317/en/>. Acesso em: 11 nov 2014.

WORLD HEALTH ORGANIZATION - WHO. (2014a). **Global health observatory data repository. Cardiovascular diseases, deaths per 100.000**. *[online]*. Disponível em: <http://apps.who.int/gho/data/node.main.A865CARDIOVASC ULAR?lang=en>. Acesso em: 06 out 2014.

WORLD HEALTH ORGANIZATION - WHO. (2014b). **Global status report on noncommunicable diseases 2014: attaining the nine global noncommunicable diseases targets, a shared responsibility.** *[online]*. Disponível em:

<http://apps.who.int/iris/bitstream/10665/148114/1/9789241564854_eng.pdf>. Acesso em: 08 dez 2014.

XAVIER, H. T.; IZAR, M. C.; FARIA NETO, J. R.; ASSAD, M. H.; ROCHA, V. Z.; SPOSITO, A. C.; et al. V diretriz brasileira de dislipidemias e prevenção da aterosclerose. **Arquivos Brasileiros de Cardiologia**, v. 101, n. 4, p. 1-20, 2013.

ZHANG, Y.; REILLY, K. H.; TONG, W.; XU, T.; CHEN, J.; BAZZANO, L. A.; et al. Blood pressure and clinical outcome among patients with acute stroke in Inner Mongolia, China. **Journal of Hypertension**, v. 26, n. 7, p. 1446-52, 2008.

ZUBIOLI, A.; SILVA, M. A. R. C. P.; TASCA, R. S.; CURI, R.; BAZOTTE, R. B. Pharmaceutical consultation as a tool to improve health outcomes for patients with type 2 diabetes. **Brazilian Journal of Pharmaceutical Sciences**, v. 49, n. 1, p. 85-94, 2013.

ZUCCHI, P.; FERRAZ, M. **Economia e gestão em saúde - Avaliação econômica em saúde.** Barueri: Manole, 2010. 155-168p.

ANEXOS

ANEXO I – Piso salarial do farmacêutico em 2013 para o estado de São Paulo (pag. 1/2).

SINDICATO DOS FARMACÊUTICOS NO ESTADO DE SÃO PAULO

SINDICATO DO COMÉRCIO VAREJISTA DE PRODUTOS FARMACÊUTICOS NO ESTADO PAULO

2. ADMITIDOS APÓS JULHO DE 2011

Obedecidos aos princípios de isonomia salarial e de manutenção das condições mais benéficas preexistentes, os salários dos empregados admitidos após julho de 2011 serão reajustados mediante a aplicação dos seguintes percentuais:

Admitidos no período de	Multiplicar o salário por
Até 15 de julho/11	1,0650
de 16/07/11 a 15/08/11	1,0594
de 16/08/11 a 15/09/11	1,0539
de 16/09/11 a 15/10/11	1,0484
de 16/10/11 a 15/11/11	1,0429
de 16/11/11 a 15/12/11	1,0374
de 16/12/11 a 15/01/12	1,0320
de 16/01/12 a 15/02/12	1,0266
de 16/02/12 a 15/03/12	1,0212
de 16/03/12 a 15/04/12	1,0159
de 16/04/12 a 15/05/12	1,0106
de 16/05/12 a 15/06/12	1,0053
A partir de 16/06/12	1,0000

2.1. Considera-se mês, fração igual ou superior a 15 (quinze dias).

2.2. Na aplicação dos índices constantes desta cláusula, o salário resultante não poderá ultrapassar aquele percebido por empregado mais antigo, na mesma função.

3. PISO PROFISSIONAL

Fica estabelecido como piso profissional a importância mensal de **R$ 2.100,00** (dois mil e cem reais).

4. SALÁRIO DE ADMISSÃO

Ao(à) farmacêutico(a) admitido(a) para exercer a função de outro(a), fica assegurada a percepção do menor salário na função, sem considerar vantagens pessoais.

5. NOVA POLÍTICA SALARIAL

Ocorrendo alteração na Política Salarial vigente, que implique em desequilíbrio nas condições ora ajustadas, as partes se comprometem a realizar tratativas em torno do tema, buscando reequilibrar o pactuado.

GARANTIAS DE EMPREGO

6. COMPROVANTES DE PAGAMENTO

Serão fornecidos obrigatoriamente, comprovantes de pagamentos, com a discriminação das importâncias pagas e os descontos efetuados, contendo a identificação da empresa e o valor dos depósitos do FGTS.

SINFAR
Rua Barão de [illegible], 295, conjuntos 304/285, Centro
CEP 01042-001 - SÃO PAULO - SP
Fone: (11) 3123 [illegible] / Fax: (11) 3221 5456
E-mail: [illegible]

SINCOFARMA
Rua [illegible]
CEP [illegible] São Paulo - SP
Fone: [illegible]
E-mail: [illegible]

ANEXO I – Piso salarial do farmacêutico em 2013 para o estado de São Paulo (pag. 2/2).

SINDICATO DOS FARMACÊUTICOS NO ESTADO DE SÃO PAULO

SINDICATO DO COMÉRCIO VAREJISTA DE PRODUTOS FARMACEUTICOS NO ESTADO PAULO

CONVENÇÃO COLETIVA DE TRABALHO

2012/2013

O ***SINDICATO DOS FARMACÊUTICOS NO ESTADO DE SÃO PAULO***, entidade sindical de primeiro grau, com sede na Rua Barão de Itapetininga, 255, conjuntos 304/305, Centro, CEP 01042-001, São Paulo, Capital, CNPJ/MF nº. 62.448.543/0001-23, Carta Sindical MTIC nº. 362.322-46, com Assembléia Geral realizada no dia 12/04/2012, em São Paulo, neste ato representado por seu Presidente, Farmacêutico Dr. **PAULO JOSÉ TEIXEIRA**, inscrito no CPF/MF nº. 121.425.758-54 e assistido por seu advogado, Fábio M. Angelini, inscrito na OAB/SP sob nº. 185.761 e o ***SINDICATO DO COMÉRCIO VAREJISTA DE PRODUTOS FARMACÊUTICOS NO ESTADO DE SÃO PAULO***, entidade sindical patronal de primeiro grau, com sede na Rua Santa Isabel, 160 – 6º andar, Vila Buarque, CEP 01221-010, São Paulo, Capital, inscrito no CNPJ/MF sob nº. 62.235.544-0001-90, com Carta de Reconhecimento Sindical, MTIC nº 17.944/1941, com Assembléia Geral realizada no dia 31 de maio de 2012 em sua sede social, São Paulo, neste ato representado por seu Presidente, Sr. **NATANAEL AGUIAR COSTA,** inscrito no CPF/MF sob nº. 434.451.108-59 e assistido por seu advogado, André Bedran Jabr, inscrito na OAB/SP sob nº. 174.840, celebram entre si, com base nos artigos 611 e seguintes da Consolidação das Leis do Trabalho, celebram entre si, com base nos artigos 611 e seguintes da Consolidação das Leis do Trabalho, a presente **CONVENÇÃO COLETIVA DE TRABALHO**, mediante as cláusulas abaixo que, reciprocamente, estabelecem, aceitam e outorgam, a saber:

SALÁRIOS, CORREÇÕES E GARANTIAS SALARIAIS

1. ATUALIZAÇÃO SALARIAL

Os salários de julho de 2011, assim considerados aqueles resultantes da aplicação integral das disposições constantes da cláusula 01 da norma coletiva imediatamente anterior a presente, serão reajustados, na data-base, qual seja, 1º de julho de 2012, em 6,5% (seis vírgula cinco por cento) a título de atualização salarial.

1.1. Os reajustes espontâneos ou compulsórios concedidos de 1º de julho de 2011 até 30 de junho de 2012 poderão ser compensados, salvo os decorrentes de promoção, transferência, equiparação salarial, implemento de idade e término de aprendizagem.

1.2. Com a aplicação da atualização salarial prevista nesta cláusula, assim como na cláusula imediatamente posterior, consideram-se integralmente satisfeitas todas as obrigações legais constantes da Lei nº. 8.880/94, obrigando-se as partes convenentes a dar por quitadas, com a aplicação da presente Convenção, todas e quaisquer eventuais diferenças salariais.

SINFAR
Rua Barão de Itapetininga, 255, conjuntos 304/305, Centro
CEP 01042-001 - SÃO PAULO - SP
Fone: (11) [illegible] / Fax: (11) [illegible]
E-mail: [illegible]

SINCOFARMA
Rua Santa Isabel, 160, Vila Buarque
CEP 01221-010 São Paulo - SP
Fone: [illegible] Fax: [illegible]
E-mail: [illegible]

ANEXO II – Aprovação do Comitê de Ética em Pesquisa da Faculdade de Ciências Farmacêuticas de Ribeirão Preto, USP (pag.1/2).

UNIVERSIDADE DE SÃO PAULO
Faculdade de Ciências Farmacêuticas de Ribeirão Preto
Comitê de Ética em Pesquisa

Of. CEP/FCFRP nº. 004/2014
kms

Ribeirão Preto, 10 de março de 2014.

Ao Pós-graduando
Maurílio de Souza Cazarim
Orientador: Prof. Dr. Leonardo Regis Leira Pereira
Departamento de Ciências Farmacêuticas
FCFRP/USP

Prezado Pesquisador,

Informamos que o Comitê de Ética em Pesquisa da FCFRP aprovou, em sua 125ª reunião ordinária, realizada em 10/02/2014, o projeto de pesquisa intitulado "AVALIAÇÃO ECONÔMICA EM LONGO PRAZO DA ATENÇÃO FARMACÊUTICA PARA PACIENTES COM HIPERTENSÃO ARTERIAL SISTÊMICA", apresentado por Vossa Senhoria a este Comitê, Protocolo **CEP/FCFRP nº. 324.**

Lembramos que, de acordo com a Resolução 466/2012, item IV.5, letra d, o TCLE deverá "ser elaborado em duas vias, rubricadas em todas as suas páginas e assinadas, ao seu término, pelo convidado a participar da pesquisa, ou por seu representante legal, assim como pelo pesquisador responsável, ou pela(s) pessoa(s) por ele delegada(s), devendo as páginas de assinaturas estar na mesma folha. Em ambas as vias deverão constar o endereço e contato telefônico ou outro, dos responsáveis pela pesquisa e do CEP local".

Informamos que deverá ser encaminhado ao CEP o relatório final da pesquisa em formulário próprio deste Comitê, bem como comunicada qualquer alteração, intercorrência ou interrupção do mesmo, tais como eventos adversos e eventuais modificações no protocolo ou nos membros da equipe, através da interposição de **emenda** na Plataforma Brasil.

Atenciosamente,

CMMachado

PROFª. DRª. CLENI MARA MARZOCCHI MACHADO
Coordenadora do CEP/FCFRP

Avenida do Café S/Nº - Monte Alegre – CEP 14040-903 – Ribeirão Preto – SP
Comitê de Ética em Pesquisa – cep@fcfrp.usp.br
Fone: (16) 3602-4213 ou 3602-4216 – Fax: (16) 3602-4892

ANEXO II – Aprovação do Comitê de Ética em Pesquisa do Centro de Saúde Escola da Faculdade de Medicina de Ribeirão Preto, USP (pag.2/2).

FACULDADE DE MEDICINA DE RIBEIRÃO PRETO
UNIVERSIDADE DE SÃO PAULO

CENTRO DE SAÚDE ESCOLA

Rua Terezina, 690 – CEP: 14055-380.
Ribeirão Preto – SP

Telefone PABX (0**16) 633-2331/4480
FAX (0**16) 6332331

COMITÊ DE ÉTICA EM PESQUISA DO CENTRO DE SAÚDE ESCOLA DA FACULDADE DE MEDICINA DE RIBEIRÃO PRETO DA UNIVERSIDADE DE SÃO PAULO-CEP/CSE-FMRP-USP.

Of.nº. 664/07/COORD. CEP/CSE-FMRP-USP- 12/12/2007.

Senhor Professor,

Temos a grata satisfação de comunicar que o Comitê de Ética em Pesquisa do Centro de Saúde Escola da Faculdade de Medicina de Ribeirão Preto da Universidade de São Paulo, em sua 66ª reunião ordinária, realizada em 11 de dezembro de 2007, analisou e apreciou o parecer do Senhor Relator, referente ao **projeto de pesquisa** intitulado: **"Atenção Farmacêutica na Hipertensão Arterial Sistêmica: impacto clínico, econômico e social".** Protocolo nº.256/CEP-CSE-FMRP-USP, que será orientado por V. Sª., a ser desenvolvido pela pós-graduanda Thais Rodrigues Penaforte, **foi aprovado.**

Lembramos que em atendimento à Resolução 196/96, deverá ser encaminhado a este CEP o relatório final da pesquisa e a publicação de seus resultados.

No ensejo, renovamos os votos de estima e consideração, despedimo-nos.

Atenciosamente,

Prof. Dr. Laércio Joel Franco
Coordenador do CEP/CSE-FMRP-USP

Ilmo. Sr.
Prof. Doutor Osvaldo de Freitas
Docente da Faculdade de Ciências Farmacêutica de Ribeirão Preto-USP.
