# Supplementary material for: Cost-effectiveness analysis of pharmaceutical care for hypertensive patients from the perspective of the public health system in Brazil
Source: PLoS One. 2018 Mar 6;13(3):e0193567. doi: 10.1371/journal.pone.0193567 (PMC5839560; doi:10.1371/journal.pone.0193567)
Supplement: S2 Appendix — Cazarim MS, Nunes AA, Pereira LRL. Cost-consequence analysis of Pharmaceutical Care program for systemic arterial hypertension in the public health system in Brazil. Braz. J. Pharm. Sci. 2017;53(3):e00217. doi: 10.1590/s2175-97902017000300217. (PDF) [file pone.0193567.s002.pdf]

S2 Appendix

Chart 1 - Description for calculating the cost of pharmaceutical care. Cazarim MS, Nunes AA, Pereira LRL. Cost-consequence analysis of Pharmaceutical Care program for systemic arterial hypertension in the public health system in Brazil. Braz. J. Pharm. Sci. 2017;53(3):e00217. doi: 10.1590/s2175-97902017000300217

Chart 1. Description for calculating the cost of pharmaceutical care

**Salary Description US$**	**Taxes**	**Amount**	**Annual (with 13th salary)**
Base salary (Sao Paulo) 40h		893,62	11.617,02
G.E.A. - Article 1 LC 391/94	0,25	223,40	2.904,26
Hazardous Activity 20%	0,01675	14,97	194,59
Additional Incentive Award	0,0835	74,62	970,02
Attendance criterion - LC 406/94	0,03	26,81	348,51
**Total**		**US$ 1.233,41**	**US$ 16.034,39**
**PC COST DESCRIPTION**			**COST**
Annual salary			16.034,39
Materials			835,34
Consultation Room			5.053,07
**Total**			**US$ 21.922,81**
Per patient			**US$ 210,80**
Per consultation			**US$ 17,57**

The materials considered were for hypertension clinical care (scale, tape measure, unit for measuring blood pressure), furniture (desk, chair, closet), general materials (computer and printer), and office supplies (clipboards, pens, plain paper, record folders, stapler, hole punch, staples, paper clips, trash basket, ruler, highlighter pen). The salary description is according to what is performed in the municipality. The base salary was obtained by the value set by the Regional Pharmacy Council as minimum wage for Pharmacists for the state of São Paulo in 2013.
